# MicroRNA expression patterns in post-natal mouse skeletal muscle development

**DOI:** 10.1186/s12864-016-3399-2

**Published:** 2017-01-07

**Authors:** Séverine Lamon, Evelyn Zacharewicz, Lauren C. Butchart, Liliana Orellana, Jasmine Mikovic, Miranda D. Grounds, Aaron P. Russell

**Affiliations:** 1 Deakin University, School of Exercise and Nutrition Sciences, Institute for Physical Activity and Nutrition (I-PAN), Geelong, Australia; 2 The University of Western Australia, School of Anatomy, Physiology and Human Biology, Perth, WA Australia; 3 Deakin University, Biostatistics Unit, Faculty of Health, Geelong, Australia

**Keywords:** MiRNAs, Skeletal muscle, Myogenesis, Growth, Cell differentiation, Cell proliferation

## Abstract

**Background:**

MiRNAs are essential regulators of skeletal muscle development and homeostasis. To date, the role and regulation of miRNAs in myogenesis have been mostly studied in tissue culture and during embryogenesis. However, little information relating to miRNA regulation during early post-natal skeletal muscle growth in mammals is available. Using a high-throughput miRNA qPCR-based array, followed by stringent statistical and bioinformatics analysis, we describe the expression pattern and putative role of 768 miRNAs in the quadriceps muscle of mice aged 2 days, 2 weeks, 4 weeks and 12 weeks.

**Results:**

Forty-six percent of all measured miRNAs were expressed in mouse quadriceps muscle during the first 12 weeks of life. We report unprecedented changes in miRNA expression levels over time. The expression of a majority of miRNAs significantly decreased with post-natal muscle maturation in vivo. MiRNA clustering identified 2 subsets of miRNAs that are potentially involved in cell proliferation and differentiation, mainly via the regulation of non-muscle specific targets.

**Conclusion:**

Collective miRNA expression in mouse quadriceps muscle is subjected to substantial levels of regulation during the first 12 weeks of age. This study identified a new suite of highly conserved miRNAs that are predicted to influence early muscle development. As such it provides novel knowledge pertaining to post-natal myogenesis and muscle regeneration in mammals.

**Electronic supplementary material:**

The online version of this article (doi:10.1186/s12864-016-3399-2) contains supplementary material, which is available to authorized users.

## Background

Skeletal muscle development (myogenesis) and homeostasis are regulated by a continuum of external and internal cell signals that activate or repress gene expression. These processes are fine-tuned by the joint action of transcription factors [1, 2], DNA methylation [3, 4], histone modification [5] and non-coding RNAs that include micro (miRNAs) and long (lncRNAs) forms [6, 7]. The role of lncRNAs has been scarcely described when compared to miRNAs; the latter playing an essential part in the control of cellular processes via transcriptional regulation [8–12].

Our study is focussed on miRNAs, which are small 20–24 nucleotide non-coding RNA molecules. In miRNA nomenclature, the species is denominated by the first 3 letters of the miRNA; where *hsa* defines a human miRNA, *mmu* defines a mouse miRNA and *dre* defines a zebrafish miRNA. MiRNAs bind to specific sites on the 3’UTR of their target transcripts and repress their translation into a functional protein [13–17]. This repression predominantly occurs by degradation of the target mRNA [15] but also by directly inhibiting protein translation. In some cases, it has been reported that miRNAs also stabilize their target mRNAs [18]. The current estimation is that more than a third of the mRNA pool possesses at least one miRNA target [19]. MiRNAs bind to mRNA 3’UTR regions on the basis of partial or full sequence homology [11, 13] and putative miRNA target sequences can be identified using freely available bioinformatics tools [20–22]. MiRNAs play a major role in the maintenance of skeletal muscle homeostasis in health and disease conditions [23]. Over the last decade, studies in tissue culture and embryogenic models have established that miRNAs are essential regulators of myogenesis. However, the role and regulation of miRNAs during post-natal skeletal muscle development in mammals has not been comprehensively described.

**Fig. 1:**
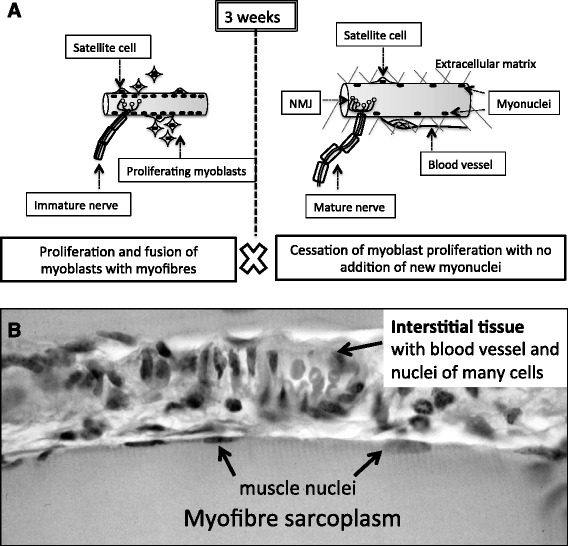
Graphical representation of key events during postnatal skeletal muscle growth (**a**) and indication of different types of cell nuclei within mature muscle tissue (**b**) **a** In mice, skeletal muscle post-natal development is comprised of 2 main growth periods. Until 3 weeks of age (left panel), satellite cells proliferate, followed by the incorporation of newly generated myonuclei into myofibres, resulting in hyperplasia. The rate of proliferation and fusion gradually declines during this period. After 3 weeks of age (right panel), the proliferation of satellite cells ceases and there is no additional incorporation of myonuclei into the myofibres. Past 3 weeks of age, muscle growth therefore results from hypertrophy only. By 3 weeks of postnatal age, the neuromuscular junction (NMJ) and innervation have matured and the vascular system and the extra-cellular matrix (ECM) are considered developed. **b** Longitudinal section of adult mouse limb muscle stained with haematoxylin and eosin. Histology shows that the bulk of the mature myofibre is occupied by sarcoplasm filled with contractile proteins, with muscle nuclei located at the surface of the myofibre. The interface with the interstitial connective tissue shows a blood vessel and various cell types within the extracellular matrix

In mice, a rapid 7–8 fold increase in body mass occurs during the first 3 weeks of post-natal life. About half of this increase is due to accretion in skeletal muscle mass [24]. Murine post-natal muscle growth almost exclusively relies on an increase in the size of muscle fibres (hypertrophy) rather than an increase in fibre number, which ceases around birth [25, 26]. Post -natal murine skeletal development consists of 2 main growth phases with a transition around 3 weeks of age, as represented in Fig. 1. In the first 3 weeks, muscle precursor cells (also known as myoblasts or satellite cells) proliferate and fuse with the rapidly elongating myofibres to provide new myonuclei (hyperplasia) (Fig. 1a) [26, 27]. From 3 to 8 weeks of age, rapid hypertrophy results in a 3-fold increase in the myofibre size [26]. This increase in sarcoplasmic volume continues until at least 14 to 28 weeks of age [27]. Early autoradiography studies tracking the in vivo kinetics of myoblast proliferation and fusion in healthy muscles of mice aged 6–8 weeks confirmed that most satellite cells were quiescent at this age [28]. The reason for this striking transition from hyperplasia to hypertrophy during muscle growth is not known and has not been widely investigated in other species (discussed in [29]).

At the molecular level, mammalian myogenesis is a finely regulated process controlled by a series of muscle specific transcription factors known as myogenic regulatory factors (MRFs). The early regulators myogenic differentiation 1 (*Myod1)* and myogenic factor-5 (*Myf5)* facilitate the commitment of satellite cells to the myogenic fate, while myogenin *(Myog/Myf4)* and myogenic factor-6 (*Myf6)* are essential for muscle cell differentiation and muscle fibre formation [30]. In addition, the paired box transcription factors (*Pax3/Pax7*) act upstream of *Myod1* to regulate the entry of satellite cells into the myogenic programme [31, 32]. MiRNAs play a role in multiple aspects muscle growth [33], including development, metabolism and repair. MRFs are involved in the transcriptional regulation of muscle enriched miRNAs (myomiRs), including miR-1, miR-133 and miR-206, and regulatory feedback loops have been identified between miRNAs and MRFs in muscle cells [26, 34–36]. Recently, our group reported the expression levels of these 3 myomiRs during post-natal muscle growth [12]. Gene expression profiling of post-natal myogenesis recently identified new genes involved in the regulation of satellite cell activation, proliferation and fusion including ephrin-related molecules. These findings suggest that myomiRs and other miRNAs may regulate more targets in skeletal muscle [37].

To date, the expression levels of a large range of miRNAs in post-natal skeletal muscle development in vivo has not been investigated. This study is the first to examine the expression pattern of 768 miRNAs and their putative gene targets and function during post-natal development of the mouse quadriceps muscle, specifically at 2 days as well as 2, 4 and 12 weeks of age.

## Methods

### Mouse muscle samples

All animals (male C57BL/6J normal mice) were obtained from the Animal Resource Centre, Murdoch, Western Australia. Mice were housed at the University of Western Australia pre-clinical facility under standard conditions, with *ad libitum* access to food and drinking water. Experiments were conducted in strict accordance with guidelines outlined in the National Health and Medical Research Council Code of practice for the care and use of animals for scientific purposes (2004), and the Animal Welfare act of Western Australia (2002). All animal experiments were approved by the Animal Ethics committee at the University of Western Australia (RA/3/100/1436). Mice aged 2, 4 and 12 weeks (n = 6 per age group) were sacrificed by cervical dislocation under terminal anaesthesia (2%v/v Attane isoflurane, Bomac, Australia). Eight 2-day old mice were sacrificed by decapitation, and due to the small muscle size the hind limb muscles from 2 animals were pooled, resulting in n = 4. All muscles were dissected out and snap frozen in liquid nitrogen before being stored at −80 °C.

### RNA extraction and reverse transcription

Total RNA was extracted from the quadriceps muscle using the miRNeasy miRNA and total RNA purification kit (Qiagen Inc., Chadstone, VIC, Australia) according to the manufacturer’s protocol. Total RNA concentration was assessed using the Nanodrop1000 Spectrophotometer (Thermo Fisher Scientific, Waltham, MA). For miRNA analysis, RNA (350 ng) was reverse transcribed using the Taqman microRNA Reverse Transcription (RT) kit and Megaplex RT Primers, Rodent Pool A and Pool B v3.0 (Life Technologies, Mulgrave, VIC, Australia). The RT reaction consisted of 2.7 mM dNTP, 0.3 U/μL RNase inhibitor, 3 mM MgCl_2_, 10 U/μL MultiScribe enzyme, 1x buffer and 1x primers. The RT conditions consisted of 40 cycles of 16 °C for two min, 42 °C for one min and 50 °C for two min, followed by 5 min at 85 °C to stop the reaction then cooled to 4 °C. For gene analysis, first-strand cDNA was generated from 500 ng RNA in 20 uL reaction buffer using the High Capacity cDNA Reverse Transcription Kit (Life Technologies); 1x RT buffer and random primers, 8 mmol/L dNTP and 2.5 U/μL MultiScribe™ RT enzyme. The RT protocol consisted of 10 min at 25 °C, 120 min at 37 °C, 5 min at 85 °C then cooled to 4 °C.

### Single-strand DNA quantification

RNA was treated with DNase I Amplification Grade (Life Technologies) and first-strand cDNA was generated as described above. cDNA was then treated with RNase H (Life Technologies) according to the manufacturer’s protocol. Single-strand DNA was quantified using the Quant it OliGreen ssDNA Assay Kit (Life Technologies) according to the manufacturer’s instructions and used for mRNA PCR normalization.

### Real-time PCR

Real-time PCR was carried out using a Stratagene MX3000 thermal cycler (Agilent Technologies, Santa Clara, CA). mRNA levels were measured using 1x SYBR® Green PCR Master Mix (Life Technologies) and 5 ng of cDNA. The PCR conditions were 1 cycle of 10 min at 95 °C; 40 cycles of 30 s at 95 °C, 60 s at 60 °C, 60 s at 72 °C; 1 cycle (melting curve) 60 s at 90 °C, 30 s at 55 °C, 30 s at 95 °C. mRNA levels were normalized to cDNA input. All primers were used at a final concentration of 300 nM. Primer sequences are presented in Table 1.

**Table 1:** Details of mouse primers for PCR analysis

Gene	GenBank accession #	Forward primer (5’-3’)	Reverse primer (5’-3’)
*Myf5*	NM_008656.5	CACCACAACCAACCCTAACCA	ACTCTCAATGTAGCGGATTGC
*Myf6*	NM_008657.2	GGTACCCTATCCCCTTGCCA	GGGAGTTTGCGTTCCTCTGA
*Myod1*	NM_010866.2	CTGCTTCTTCACGCCCAAA	CTGGAAGAACGGCTTCGAAAG
*MyoG*	NM_031189.2	TCCATCGTGGACAGCATCAC	CAATCTCAGTTGGGCATGGTTT
*Pax3*	NM_008781.4	AAACCCAAGCAGGTGACAAC	CTAGATCCTCCTCCTCT
*Pax7*	NM_011039.2	GAGTTCGATTAGCCGAGTGC	GTCGGGTTCTGATTCCACAT

### MiRNA screening

MiRNA expression in the samples was assessed using the TaqMan Array Rodent MicroRNA A + B Cards v3.0 (Applied Biosystems, Mulgrave, VIC, Australia). Collectively, these cards allow for the accurate quantitation of 768 mouse and rat miRNAs. The cards also contain 3 candidate endogenous controls that are specific to mouse and can be used for normalisation and an irrelevant miRNA that serves as a negative control and ensures that there is no non-specific amplification of targets. The results from the Megaplex were then analysed using ExpressionSuite Software v1.0 (Applied Biosystems) and the data were normalized using the global normalization function included in the software, a technique that more accurately represents biological variation than a selected number of endogenous controls [38, 39]. Global normalisation applies a constant scaling factor to every measurement in a qPCR well, so that they all have the same median intensity. Because a constant scaling factor is applied, the global normalisation does not change the relative expression of individual miRNAs in each well, while accounting for biological and methodological variations. For individual miRNA expression levels, Ct values were then transformed into arbitrary units (AU) using the following equation: *AU*=(1/2)^*Ct*^10^10^ and expressed relative to the mean value of the latest time point each miRNA was expressed at.

### Statistical analysis

A first exploratory analysis of the data revealed a large number of miRNAs with outlier Ct values. Therefore, robust regression (M-estimation with scale parameter estimated using the median method) was used to estimate the linear trend in Ct values with time. For miRNAs with no significant trend on time, Kruskal-Wallis’ test was used to compare Ct values between times. Given the exploratory nature of the study, we chose not to correct for multiple comparisons to minimize the risk of false negative results.

With the aim of identifying groups of expression patterns versus time, quadratic models including time and time^2^ were adjusted using the same robust approach. A hierarchical clustering algorithm based on the Ward’s minimum variance criterion was used to group the predicted Ct profiles into clusters. All regression analyses were performed with SAS software, version 9.3 (SAS Institute, Cary, NC).

In addition to the regression models, the individual expression levels of the MRFs, a subset of miRNAs including some myomiRs as well as the top-ranked miRNAs of each cluster (see below) were calculated. Arbitrary unit values were analyzed using a one-way analysis of variance (ANOVA) using GenStat v16 [40]. Diagnostic plots of residuals and fitted values were checked to ensure homogeneity of variance (a key assumption for ANOVA). The significance level was set at *p* < 0.05.

### Bioinformatics

For each selected cluster, the top cellular functions and miRNA-mRNA target interactions were determined using Ingenuity System Interactive Pathway Systems (version 18488943). Stringency was set at ‘highly predicted’ and ‘experimentally validated’. The software uses its own internal algorithm and other databases, including TarBase, TargetScan and miRecords, as well as findings published in the literature. Ingenuity pathway analysis (IPA) was used to generate figures depicting the relationship between miRNAs and predicted target mRNAs involved in cell cycle and proliferation, cell differentiation and organismal development. Individual target predictions were made using miRwalk [22].

## Results

### MiRNA expression levels during skeletal muscle development

The cut-off for the relevant level of expression of each miRNA was set at Mean (Ct) < 32 for each specific time point, as recommended by the manufacturer. Out of the 768 miRNAs measured, 415 (54%) were considered not expressed in mouse quadriceps muscle and were therefore excluded from further analysis. 310 miRNAs (40%) were expressed at all time points, 42 miRNAs (5%) were expressed at 2 or 3 time points, while one miRNA (0.1%) was expressed at the 2-day time point only (Additional file 1).

**Fig. 2:**
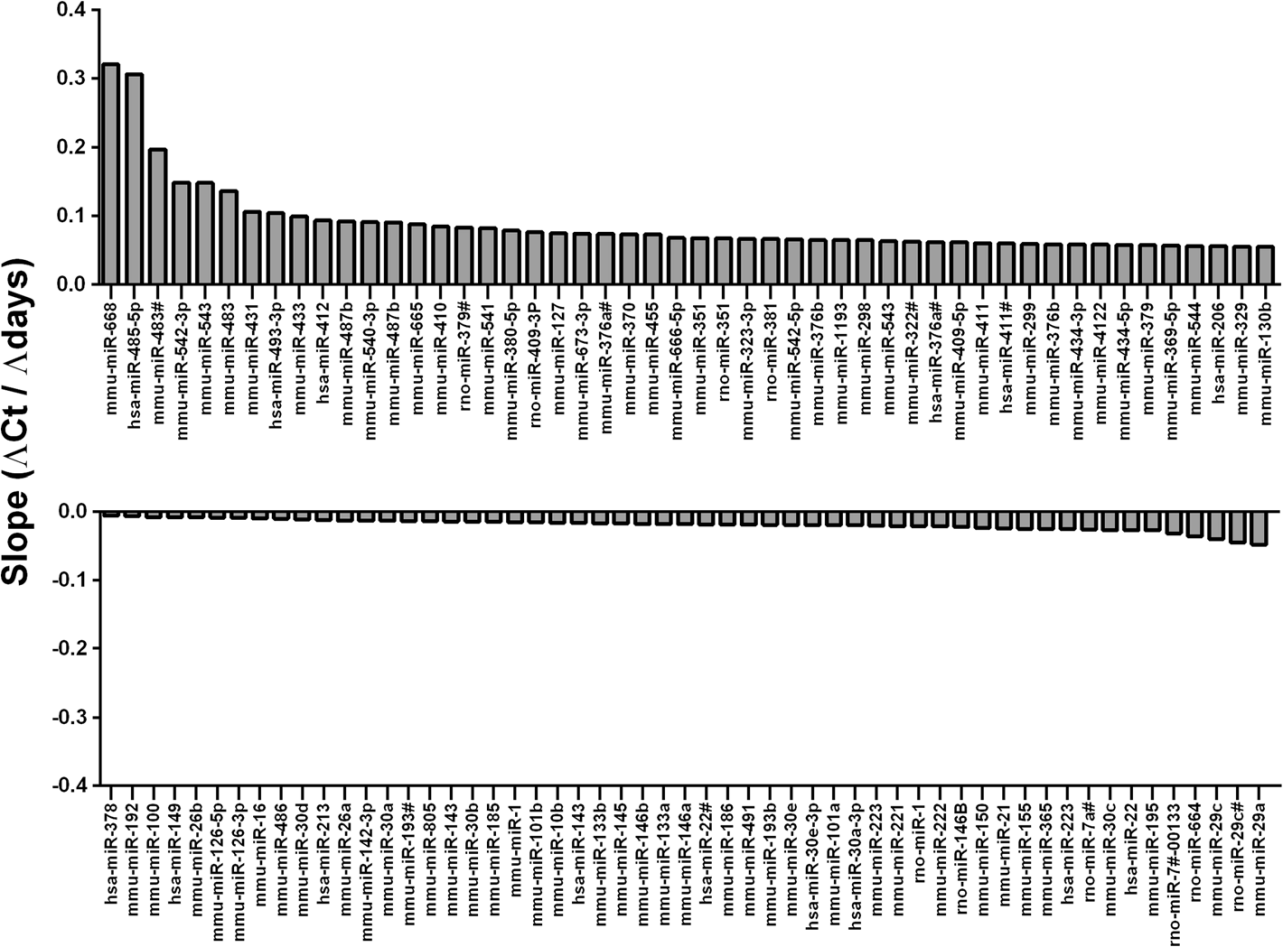
Statistically significant slopes (ΔCt/ΔDays) estimated using a robust regression model. The top-50 significant positive slopes (upper panel) and the top-50 significant negative slopes (lower panel) are represented. Positive slopes indicate that the expression of miRNAs decreases over time. Negative slopes indicate that the expression of miRNAs increases over time

Linear trends in Ct values were assessed using a robust regression model. Out of the 310 miRNAs expressed at all time points, 205 (66%) presented a significant linear trend in their Ct values over time. For 150 miRNAs the Ct values increased over time (i.e. their expression levels logarithmically decreased), while for 55 miRNAs the Ct values decreased over time (i.e. their expression levels logarithmically increased). The top-50 positive slopes and the 50 negative slopes are individually reported in Fig. 2. A comprehensive list of all significant slopes is displayed in Additional file 2.

The expression pattern of 37 miRNAs that did not present a linear trend showed significant changes between times when applying a non-parametric test (Kruskal-Wallis’ test; Additional file 3). Overall, 238 out of 310 miRNAs (77%) displayed a significant change over time.

### Expression levels of myomiRs with time

**Fig. 3:**
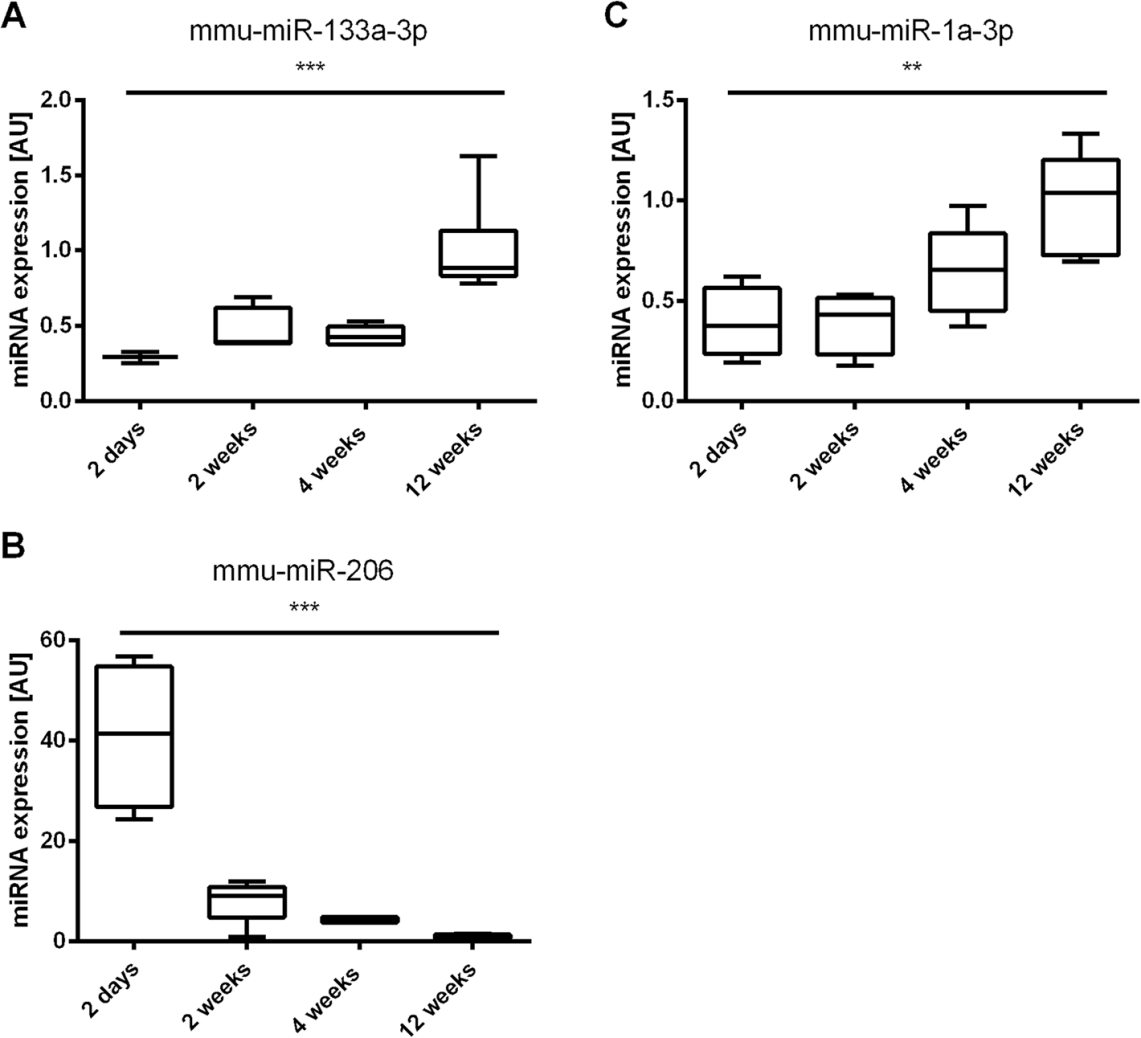
MyomiRs expression levels over time. Box-plots of miRNA expression levels of the myomiRs mmu-miR-1-3p (**a**), mmu-miR-133a-3p (**b**) and hsa-miR-206 (**c**) in mouse quadriceps muscle at 2 days, 2 weeks, 4 weeks and 12 weeks after birth. **, main effect of time, *p* < 0.01. ***, main effect of time, *p* < 0.001. The data are reported as mean ± SEM

MyomiRs are expressed in muscle cells (skeletal and cardiac) [33, 41]. The individual expression levels of the myomiRs mmu-miR-1-3p, mmu-miR-133a-3p and hsa-miR-206 are reported in Fig. 3. MiR-133a and miR-1 both significantly increased with time (main effect of time, *p* < 0.001 and *p* < 0.01, respectively), while miR-206 expression significantly decreased with time (main effect of time, *p* < 0.001).

### MiRNA clustering

**Fig. 4:**
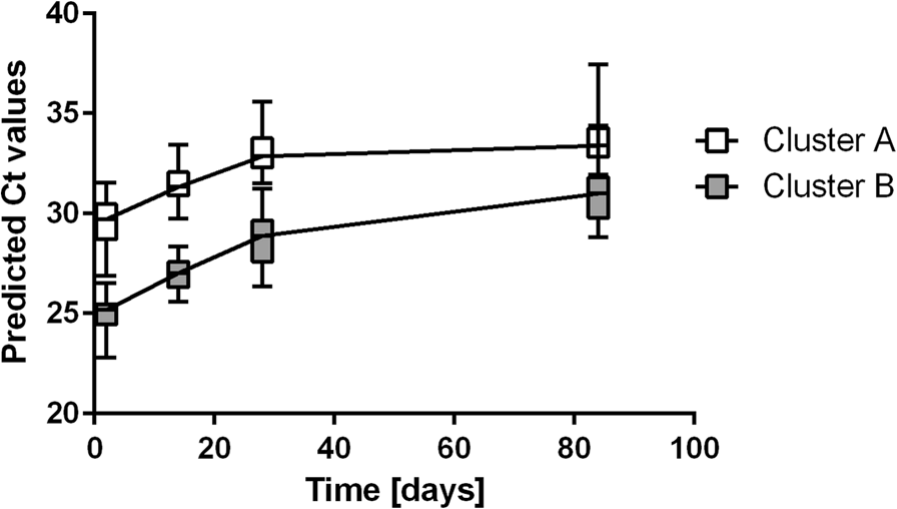
MiRNA clusters. X-axis: time [days], Y-axis: average predicted Ct value of all miRNAs of cluster A and B under a quadratic model in time (robust regression). The data are reported as mean ± standard deviation (SD)

MiRNA expression profiles were classified in 10 clusters using the predicted Ct values from a robust quadratic model (Additional file 4). The 2 clusters that presented the largest changes in predicted Ct values over time (referred to as cluster A and cluster B) were considered to have the highest biological relevance and were selected for bioinformatics analysis (Fig. 4). Cluster A included 44 miRNAs that were moderately expressed, with Ct values between 27 and 32 PCR cycles for the 2-day age group. The typical Ct profile for this cluster corresponds to expression levels that decrease during the first 4 weeks of life and remain mostly stable from 4–12 weeks. In contrast, cluster B included 28 miRNAs with higher initial expression levels that were detected between 23 and 26 PCR cycles for the 2-day age group. Their predicted expression levels generally displayed a gradual decrease over the whole period studied. The predicted Ct values for all miRNAs from cluster A and B are provided in Additional file 5.

### Ingenuity system interactive pathway systems analysis

Core analysis was performed on all miRNAs comprised in a specific cluster. “Cellular Development” and “Cellular Growth and Proliferation” were the 2 highest ranked Molecular and Cellular Functions returned for cluster A, with *p*-values respectively comprised between 3.49E-02 – 1.8E-03 and 3.49E-02 – 6.3E-05. In contrast, the miRNAs included in cluster B were significantly associated with the regulation of Organismal Development (*p* = 3.62E-02 – 3.28E-07) and Skeletal and Muscular Disorders (*p* = 7.5E-03 – 1.7E-03).

Target analysis was completed on all miRNAs included in cluster A and cluster B (Additional file 6). A list of predicted and validated mRNA targets for the 44 miRNAs from cluster A and the 28 miRNAs from cluster B was generated using Ingenuity software prediction algorithms. Putative gene targets were considered for further analysis if they were returned as either “highly predicted” and/or “experimentally observed”. For each cluster, the list of genes was then sorted on the basis of the molecular pathways that were associated with each gene, and genes were categorized using the following key words: “cell cycle and proliferation”, “cell differentiation”, “organismal development” and “skeletal muscle”. Cell cycle and organismal development were the most highly ranked cellular processes predicted to be targeted by the miRNAs in clusters A and B. For cluster A, 7% and 5% of all predicted gene targets were associated with cell cycle and organismal development, respectively, while these categories both represented 3% of the genes predicted to be targeted by the members of cluster B. Muscle specific genes only represented a very minor proportion of the predicted targets for both clusters (Table 2).

**Table 2:** Biological classification of predicted mRNA targets

Biological classification of predicted mRNA targets	Cluster A [number of targets, %]	Cluster B [number of targets, %]
Cell cycle	56, 6.5%	15, 3.1%
Development	39, 4.5%	14, 2.9%
Differentiation	14, 1.6%	9, 1.9%
Skeletal muscle	2, < 0.5%	1, < 0.5%
Others	751, 87.1%	440, 91.8%

mRNA transcripts predicted to be targeted by the miRNAs members of cluster A and cluster B were grouped on the basis of the cellular functions they are associated with (key words used: “cell cycle and proliferation”, “cell differentiation”, “organismal development” and “skeletal muscle”)

**Fig. 5:**
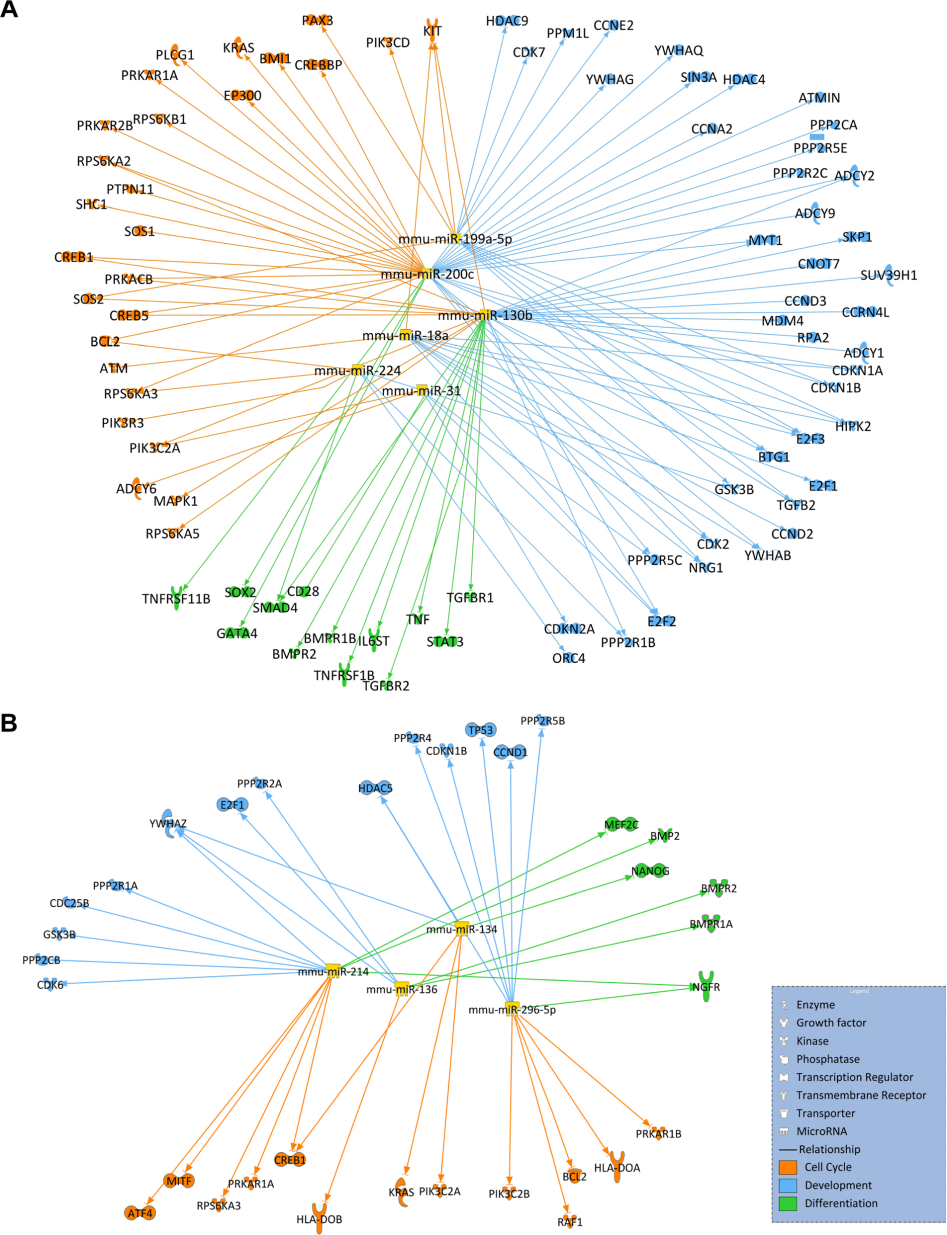
Graphical representation of the relationships existing between the top ranked miRNAs and their target genes. Top-ranked miRNAs for cluster A (upper panel) and B (lower panel) and their predicted gene targets within the proliferation, cell cycle and organismal development pathways. Cell cycle regulators are shown in orange. Cell development regulators are shown in blue. Cell differentiation regulators are shown in green

Following this, the genes known to regulate cell cycle and proliferation, cell differentiation and organismal development were selected to generate the figures illustrating the predicted role of the top-ranked miRNAs of each cluster in the proliferation, differentiation and development signalling cascades during the first 12 weeks of age (Fig. 5). The top-ranked miRNAs for clusters A were mmu-miR-18a-5p, mmu–miR-31–5p, mmu-miR-130b–5p, mmu-miR-199a–5p, mmu-miR-200c–5p and mmu-miR-224–5p. The top-ranked miRNAs for cluster B were mmu-miR-134–5p, mmu-miR-136–5p, mmu-miR-214–3p and mmu-miR-295–5p.

### Individual expression levels of the top-ranked miRNAs for each cluster and muscle specific predicted targets

**Fig. 6:**
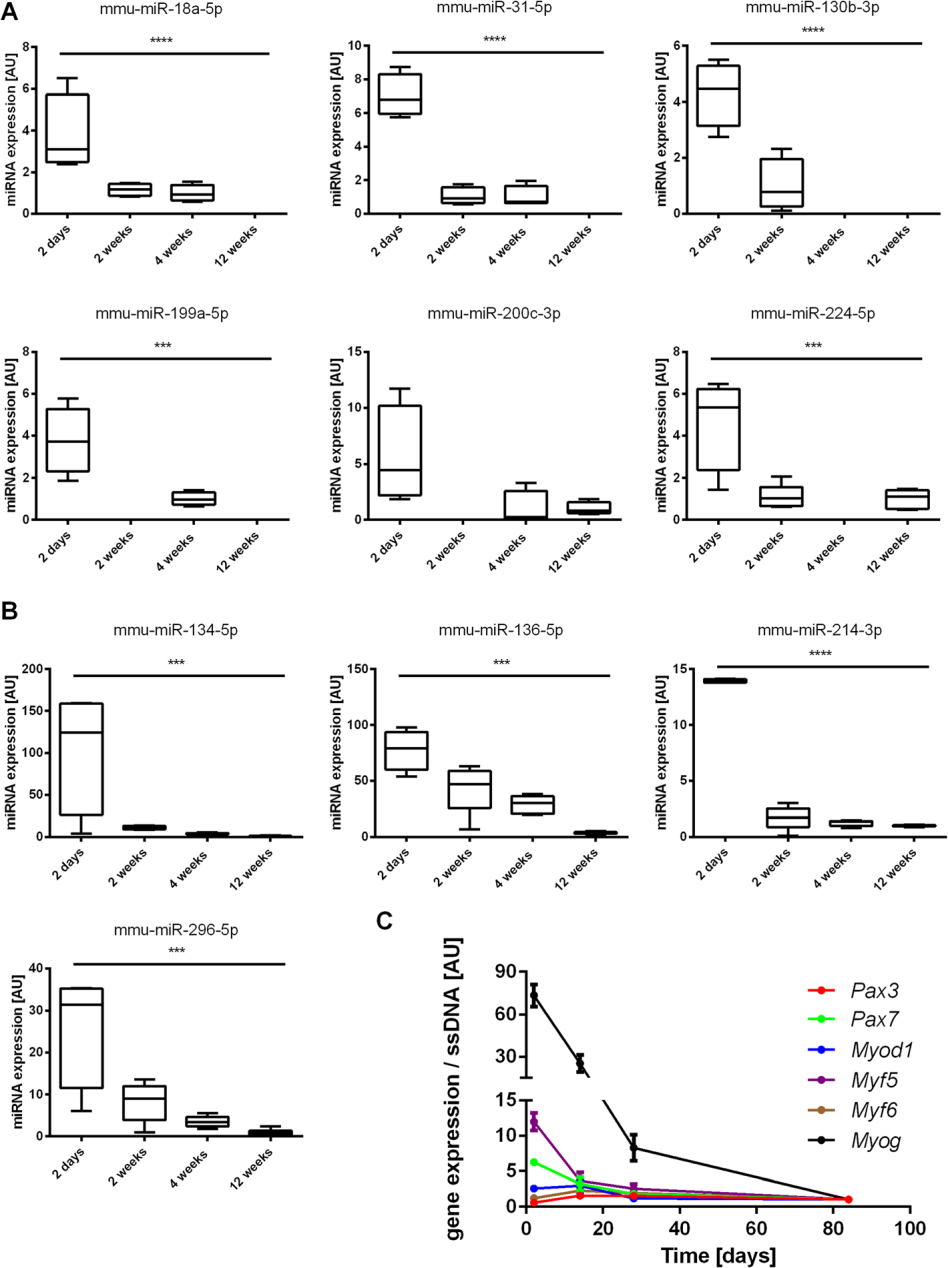
Expression levels of the top-ranked miRNAs of cluster A (**b**) and B (**b**) and MRFs and Pax family members over time (**c**). **a** Box-plots of miRNA expression levels of mmu-miR-18a-5p, mmu-miR-31-5p, mmu-miR-130b-3p, mmu-miR-199a-5p, mmu-miR-200c-3p, mmu-miR-224-5p in mouse quadriceps muscle at 2 days, 2 weeks, 4 weeks and 12 weeks after birth. ***, main effect of time, *p* < 0.001. ****, main effect of time, *p* < 0.0001. The data are reported as mean ± SEM. **b** Box-plots of miRNA expression levels of mmu-miR-134-5p, mmu-miR-136-5p, mmu-miR-214-3p, mmu-miR-296-5p in mouse quadriceps muscle at 2 days, 2 weeks, 4 weeks and 12 weeks after birth. ***, main effect of time, *p* < 0.001. ****, main effect of time, p < 0.0001. The data are reported as mean ± SEM. **c** Gene expression levels of *Pax3, Pax7, Myod1, Myf5, Myf6* and *Myog* at 2 days, 2 weeks, 4 weeks and 12 weeks after birth were measured by qPCR. Gene expression data were normalized to single-strand DNA content. A main effect of time was reported for *Pax7, Myod1*, *Myf5*, and *Myog* (all *p* < 0.05). The data are reported as mean ± SEM

The individual expression levels of the top-ranked miRNAs of each cluster are depicted in Fig. 6 (a and b). For each miRNA, individual predictions were conducted to investigate putative muscle-specific gene targets including *Pax3, Pax7, Myog, Myod1, Myf5* and *Myf6* (Table 3). Of the 10 miRNAs of interest, 9 had at least one highly predicted muscle-specific target.

**Table 3:** Predicted muscle-specific gene targets for the top-ranked miRNAs of cluster A and B

miR ID	Cluster	Highly predicted target	Moderately predicted target	Homology with human miRNA
mmu-miR-18a-5p	A	*Pax7*		hsa-miR-18a-5p
mmu-miR-31-5p	A	*Pax3, Myf5, Pax7*		hsa-miR-31-5p
mmu-miR-130b-3p	A	*Myod1*		hsa-miR-130b-3p
mmu-miR-199a-5p	A	*Pax3*	*Pax7, Myod1*	hsa-miR-199a-5p
mmu-miR-200c-3p	A		*Myf5*	hsa-miR-200c-3p
mmu-miR-224-5p	A	*Myod1*	*Pax7*	*First nt different from* hsa-miR-224-5p
mmu-miR-134-5p	B	*Pax7*	*Myf5*	hsa-miR-134-5p
mmu-miR-136-5p	B	*Pax3*	*Myog*	*Last nt different* from hsa-miR-136-5p
mmu-miR-214-3p	B	*Pax7*	*Pax3*	hsa-miR-214-3p
mmu-miR-296-5p	B	*Pax7*		hsa-miR-296-5p

Muscle-specific gene targets for the top-ranked miRNAs of cluster A and B were predicted using the miRNA/gene prediction tool in miRwalk v.2.0 [22]. A target was considered as “highly predicted” when returned positive by at least 3/4 prediction software and as “moderately predicted” when returned positive by 2/4 prediction software. The last column displays the name of the corresponding human miRNA. All miRNAs identified in this study were perfectly or highly homologous between mouse and human

### Gene expression levels of myogenic regulatory factors (MRFs) during skeletal muscle growth

The MRF genes, including *Myod1, Myf5, Myf6 and Myog*, and Pax genes, including *Pax3* and *Pax7,* are the 2 major families of genes orchestrating the myogenesis process. All the miRNAs identified in our screening were predicted to target at least one of these genes. The gene expression levels of *Myod1, Myf5, Myf6, Myog*,* Pax3* and *Pax7* were assessed in the quadriceps muscle of male C57BL/6J mice during post-natal muscle development (2 days to 12 weeks). *Myog* (73-fold), *Myf5* (12-fold), *Pax7* (6.3-fold) and *Myod1* (2.6-fold) displayed significant decreases between 2 days and 12 weeks after birth (main effect of time, all p < 0.05). There was no effect of time on *Myf6* and *Pax3* expression levels (Fig. 6c).

## Discussion

In this study, we describe for the first time the collective and individual expression patterns of a subset of 768 miRNAs in mouse quadriceps muscle tissue during the first 12 weeks of post-natal life. We report unprecedented changes in the miRNA expression levels over time, supporting the hypothesis that miRNAs play an essential role in the regulation of cellular processes underlying skeletal muscle formation and maturation. Stringent statistical and bioinformatics analysis confirmed the putative role of these miRNAs in the regulation of the proliferation, differentiation and development pathways, mainly via the regulation of gene targets that were not specifically linked to skeletal muscle, but to the development of a variety of tissues. The additional genetic regulation exerted by lncRNAs [12] falls outside the scope of this study.

Over the last 2 decades, miRNAs have emerged as important regulators of molecular processes in cells in general [8–11] and in skeletal muscle in particular [12, 23, 33]. MiRNA detection is commonly achieved using real-time quantitative PCR, with commercially available PCR-based miRNA arrays allowing the simultaneous quantification of hundreds of miRNAs [42, 43]. While a lack of consensus exists around the most suitable normalization strategy for single miRNA PCR analysis [44], the use of PCR-based miRNA arrays allows for the normalization of the individual cycle threshold (Ct) values to the geometric mean of the whole miRNA pool, a process referred to as global normalization [45]. Global normalization rules out the occurrence of false-positive results due to a lack of stability of a single normalization miRNA over different times and conditions. This advantage is particularly relevant in a growth model as extreme as the one used in this study.

MiRNAs can be specifically enriched in certain tissues [46] and myomiR is the term referring to the miRNA species that are highly expressed in skeletal and cardiac muscle [41, 47]. These miRNAs are involved in the regulation of all fundamental biological processes in the muscle, including growth, development, metabolism and repair [33]. MiR-1, miR-133a and miR-206 were amongst the 3 first identified myomiRs, with miR-1 and miR-133a being part of a bicistronic cluster on the same chromosome [36]. Their role in myogenesis has been well described in vitro using gain— and loss-of-function studies. MiR-1, miR-133a and miR-206 are highly conserved between species. Our study respectively reported a 4-fold and 3-fold increase in mmu-miR-1a-3p and mmu-miR-133a-3p expression levels over the first 12 weeks of age. MiR-133a and miR-1 respectively promote myoblast proliferation and differentiation via the repression of *Srf* and *Hdac4* [36]. In contrast, the expression of hsa-miR-206, an indirect activator of *Myod1* [48], decreased by 40-fold between birth and 12 weeks of age. Similar to miR-1, miR-206 promotes satellite cell differentiation and suppresses cell proliferation by directly targeting the *Pax7* 3’UTR [48], suggesting a reduced need to engage stem cells into the myogenic fate during the later stages of muscle growth. This is in line with the in vivo pattern of cessation of satellite cell proliferation and fusion by 3 weeks of age in mice [26].

Beyond the highly expressed myomiRs, we report that 46% of all measured miRNAs were expressed in mouse quadriceps muscle over the first 12 weeks of life. The expression levels of a majority of miRNAs decreased with age, as the incidence and amplitude of many regulative processes become less important. Statistical analysis followed by miRNA clustering allowed the isolation of 2 subsets of miRNAs displaying common expression patterns that were selected for bioinformatics analysis. The predicted expression levels of the members of cluster A were below or close to the 32 Ct limit of detection between 4 and 12 weeks of age. These miRNAs may therefore be involved in the regulation of very early cell processes, and conceivably play an even more important role in pre-natal (foetal and embryonic) muscle formation and growth. Accordingly, cellular development, growth and proliferation were the highest ranked molecular functions returned for cluster A. In contrast, the members of cluster B were consistently expressed from week 4 to week 12 and were strongly linked to organismal development. This suggests that, in addition to the development of the muscle fibre itself, these miRNAs may regulate the development of other cell components in the growing muscle tissue (see Fig. 1). Indeed, it is important to acknowledge that other cell types outside the myofibre contribute to the dynamics of muscle growth. These are associated with neuromuscular function (nerves and Schwann cells), vascularization (endothelium, smooth muscle and bone-marrow derived cells) and formation of the extracellular matrix (ECM), whose components are largely produced by fibroblasts [49]. While skeletal muscle fibres represent about 95% of the volume of muscle tissue, much of this consists of sarcoplasm containing large contractile proteins with relatively sparse myonuclei (see Fig. 1b). Furthermore, the number of nuclei associated with adult myofibres only represent about half of all nuclei present in muscle tissue [50, 51]. The other 50% of nuclei outside the myofibre are within different types of mononucleated cells associated with the nervous and the vascular systems ﻿(see Fig. 1b), as well as the interstitial connective tissue containing various cells, including fibroblasts. Although in adult muscle tissue, myofibres are patently the most transcriptionally active cell type [52], the transcriptome activity of muscle tissue includes the combined expression of the myogenic nuclei, plus the various non-myogenic nuclei, in a ratio that may vary with development. Thus, these aspects must be considered when interpreting patterns of gene expression, especially related to miRNAs. Finally, other miRNAs from cluster B have also been significantly associated with muscular disorders in adults, indicating that these miRNAs are presumably more active at the level of the post-natal than the pre-natal developing muscle.

Sparse literature exists around the top-ranked miRNAs identified in this study and their role in the cell proliferation and differentiation processes. Although not selected for bioinformatics analysis in this study, the top-3 miRNAs displaying an increase in expression with age were members of the miR-29 family of miRNAs (mmu-miR-29a, rno-miR-29c# and mmu-miR-29c), supporting the important role played by this miRNA cluster in the myogenesis process. MiR-29a inhibition downregulates *MyoD *and upregulates cyclin-dependent kinase 6 (*Cdk6*) expression in C_2_C_12_ myoblasts, therefore delaying cell differentiation [53]. In contrast, miR-29 is upregulated in the muscle of aged when compared to young rodents. Corroborating our findings, miR-29 electroporation into the muscle of young mice suppressed cell proliferation and accelerated aging [54]. Several additional gene targets have been identified for the miR-29 family of miRNAs, including Akt3 [55] and Ring1 and YY1 binding protein (Rybp) [55], a negative regulator of myogenesis. Amongst the top-ranked miRNAs of cluster A, mmu-miR-199a– 5p (homologous to hsa-miR-199a–5p and dre-miR-199a–5p) regulates myogenic differentiation acting downstream of *Srf;* the latter targets multiple differentiation and proliferation factors within the Wnt signalling pathway. Hsa-miR-199a-5p expression is increased in human dystrophic muscle and overexpressing dre-miR-199a-5p in zebrafish muscle leads to major and lethal disruption of the myofibers [56]. In line with our observations, hsa-miR-31-5p (homologous to mmu-miR-31-5p) expression is increased in regenerating [57] and dystrophic muscle [57, 58] and positively regulates the proliferation of vascular smooth muscle cells [59]. Hsa-miR-31-5p directly targets the 3’UTR of human Hdac4 and Nrf1 [60], as well an evolutionary conserved sequence of dystrophin 3’UTR [61]. Therefore, it likely plays a multifaceted role in the regulation of skeletal muscle development. The other members of miRNA cluster A have received little attention, especially in skeletal muscle. Hsa-miR-18a-5p (homologous to mmu-miR-18a-5p) enhances cell apoptosis in human keratinocytes in vitro [62] and stimulates the protein expression of the vascular smooth muscle cell differentiation markers *Acta2* and *Tagln* [63] and mmu-miR-224-5p negatively regulates mouse adipocyte differentiation [64]. This suggests that mmu-miR-18a-5p and mmu-miR-224-5p may play roles in the early stages of the skeletal muscle tissue development, including vascular and adipocyte differentiation.

In contrast, the top-ranked miRNAs of cluster B have been relatively well described in skeletal muscle. Next-generation sequencing (NGS) technology revealed that mmu-miR-136-5p is decreased in gastrocnemius muscle from mice aged 6 and 24 months [65] and targets *Rybp*. This corroborates our results where mmu-miR-136-5p expression levels decrease by close to 80-fold between the age of 2 days and 12 weeks in mouse quadriceps muscle. In porcine skeletal muscle, the homologous ssc-miR-214-3p is highly expressed in the foetal stages (embryonic day 90) when compared with post-natal levels (post-partum day 120) [66]. This is in line with our findings in mouse muscle, where expression levels at 12 weeks of age were 14-fold greater than at 2 days. Mmu-miR-214-3p inhibits proliferation and promotes differentiation of the immortalized C2C12 mouse myogenic cell line in vitro [67]. Supressing mmu-miR-214-3p expression maintains C2C12 myoblasts in an active cell cycle and inhibits myogenic differentiation [68]. Our data also show that mmu-miR-214-3p is highly predicted to target *Pax7* and we propose that it may act as a negative regulator of myoblast proliferation in vivo by directly regulating the *Pax7/Myod1* pathway. Of interest, all 4 top-ranked miRNAs in cluster B were predicted to target one of the Pax gene family members with a high degree of certainty, potentially indicating a similar role for mmu-miR-134-5p, mmu-miR-136-5p and mmu-miR-296-5p. Mmu-miR-296-5p overexpression almost totally suppresses the utrophin-A protein in C2C12 myoblasts [69]. Utrophin is a cytoskeletal protein with similarities to dystrophin. Utrophin is initially expressed by all myonuclei and is located around the entire sarcolemma during foetal and early post-natal myofibre growth. However, by 3 weeks of age, utrophin protein expression is restricted to beneath the NMJ [70]. The expression pattern observed for mmu-miR-296-5p (down regulated by 25-fold between 2 days and 12 weeks of age) supports a potential role as an utrophin suppressor during myofibre maturation. Finally, mmu-miR-134-5p (down regulated by close to 100-fold between 2 days and 12 weeks of age) has not been described in skeletal muscle. It enhances differentiation in mouse embryonic stem cells [71] and also promotes cell proliferation while preventing the induction of differentiation and apoptosis in neuronal progenitor cells [72]. Whether mmu-miR-134–5p has a similar role in the muscle remains to be elucidated.

This study is the first to assess the expression levels of a large pool of miRNAs in an in vivo model of muscle development. A similar array has been previously conducted in a mouse model of muscle regeneration [73] and several miRNAs, including mmu-miR-18a-5p, mmu-miR-136-5p, mmu-miR-31-5p and mmu-miR-199a-5p, were similarly regulated as observed in our study. Muscle regeneration may display similarities with post-natal muscle development, although adult skeletal muscle tissue possesses quiescent stem cells that differ from developing tissues in aetiology and properties [74].

## Conclusion

In conclusion, collective miRNA expression is subjected to substantial levels of regulation in mouse skeletal muscle tissue over the first 12 weeks of age. This study identified a suite of highly conserved miRNAs that are predicted to control muscle cell proliferation and differentiation pathways with a high degree of certainty. The specific role of these miRNAs in skeletal muscle development, maturation, function and disturbed homeostasis now requires expansion to a range of muscles in a range of species and experimental validation in vivo.

## Additional files

Additional file 1: Descriptive analysis of all Ct values over time. (PDF 349 kb)
Additional file 2: List of the 205 miRNAs presenting a significant linear trend in their Ct values over time. 150 miRNAs had their Ct values increasing over time, while 55 had their Ct values decreasing over time. (PDF 102 kb)
Additional file 3: Kruskal-Wallis’ tests were used to compare time Ct values for miRNAs with no significant trend. Data are presented as median and 25^th^ and 75^th^ percentile. (PDF 45 kb)
Additional file 4: MiRNAs expression profiles were classified in 10 clusters using the predicted Ct values from a robust quadratic model. (PDF 112 kb)
Additional file 5: Predicted Ct values for all miRNAs from cluster A and B. (PDF 103 kb)
Additional file 6: Target analysis of all miRNAs included in cluster A and cluster B. (XLSX 370 kb)

## References

[1] Keller C, Steensberg A, Pilegaard H, Osada T, Saltin B, Pedersen BK, Neufer PD (2001). Transcriptional activation of the IL-6 gene in human contracting skeletal muscle: influence of muscle glycogen content. FASEB J Official Pub Fed Am Soc Exp Biol.

[2] McGee SL, Sparling D, Olson AL, Hargreaves M (2006). Exercise increases MEF2- and GEF DNA-binding activity in human skeletal muscle. FASEB J Official Pub Fed Am Soc Exp Biol.

[3] Barres R, Yan J, Egan B, Treebak JT, Rasmussen M, Fritz T, Caidahl K, Krook A, O’Gorman DJ, Zierath JR (2012). Acute exercise remodels promoter methylation in human skeletal muscle. Cell Metab.

[4] Nakajima K, Takeoka M, Mori M, Hashimoto S, Sakurai A, Nose H, Higuchi K, Itano N, Shiohara M, Oh T (2010). Exercise effects on methylation of ASC gene. Int J Sports Med.

[5] McGee SL, Fairlie E, Garnham AP, Hargreaves M (2009). Exercise-induced histone modifications in human skeletal muscle. J Physiol.

[6] Wang Kevin C, Chang Howard Y (2011). Molecular mechanisms of long noncoding RNAs. Mol Cell.

[7] Sun M, Kraus WL (2015). From discovery to function: the expanding roles of long NonCoding RNAs in physiology and disease. Endocr Rev.

[8] Lee RC, Feinbaum RL, Ambros V (1993). The C. elegans heterochronic gene lin-4 encodes small RNAs with antisense complementarity to lin-14. Cell.

[9] Reinhart BJ, Slack FJ, Basson M, Pasquinelli AE, Bettinger JC, Rougvie AE, Horvitz HR, Ruvkun G (2000). The 21-nucleotide let-7 RNA regulates developmental timing in caenorhabditis elegans. Nature.

[10] Bartel DP (2004). MicroRNAs: genomics, biogenesis, mechanism, and function. Cell.

[11] Brodersen P, Voinnet O (2009). Revisiting the principles of microRNA target recognition and mode of action. Nat Rev Mol Cell Biol.

[12] Butchart LC, Fox A, Shavlakadze T, Grounds MD: The long and short of non-coding RNAs during post-natal growth and differentiation of skeletal muscles: Focus on lncRNA and miRNAs. Differentiation; research in biological diversity 2016;92(5):237–48. 10.1016/j.diff.2016.05.003 27292314

[13] Brennecke J, Stark A, Russell RB, Cohen SM (2005). Principles of MicroRNA–target recognition. PLoS Biol.

[14] Hu Z, Bruno AE: The Influence of 3‘UTRs on MicroRNA Function Inferred from Human SNP Data. Comparative & Functional Genomics 2011;910769:1–9. 10.1155/2011/910769 PMC3202110 22110399

[15] Huili G, Ingolia NT, Weissman JS, Bartel DP (2010). Mammalian microRNAs predominantly act to decrease target mRNA levels. Nature.

[16] Humphreys DT, Westman BJ, Martin DIK, Preiss T (2005). MicroRNAs control translation initiation by inhibiting eukaryotic initiation factor 4E/cap and poly (A) tail function. (English). Proc Natl Acad Sci U S A.

[17] Pillai RS, Bhattacharyya SN, Artus CG, Zoller T, Cougot N, Basyuk E, Bertrand E, Filipowicz W (2005). Inhibition of translational initiation by Let-7 MicroRNA in human cells. Science.

[18] Vasudevan S, Tong Y, Steitz JA (2007). Switching from repression to activation: MicroRNAs Can Up-regulate translation. Science.

[19] Beitzinger M, Peters L, Zhu JY, Kremmer E, Meister G (2007). Identification of human microRNA targets from isolated argonaute protein complexes. RNA Biol.

[20] Lewis BP, Shih I, Jones-Rhoades MW, Bartel DP, Burge CB (2003). Prediction of mammalian MicroRNA targets. Cell.

[21] Dweep H, Sticht C, Pandey P, Gretz N (2011). MiRWalk – database: prediction of possible miRNA binding sites by “walking” the genes of three genomes. J Biomed Inform.

[22] Dweep H, Gretz N (2015). miRWalk2.0: a comprehensive atlas of microRNA-target interactions. Nat Methods.

[23] Zacharewicz E, Lamon S, Russell AP (2013). MicroRNAs in skeletal muscle and their regulation with exercise, ageing, and disease. Front Physiol.

[24] Gokhin DS, Ward SR, Bremner SN, Lieber RL (2008). Quantitative analysis of neonatal skeletal muscle functional improvement in the mouse. J Exp Biol.

[25] Ontell M, Feng KC, Klueber K, Dunn RF, Taylor F (1984). Myosatellite cells, growth, and regeneration in murine dystrophic muscle: a quantitative study. Anat Rec.

[26] White RB, Bierinx AS, Gnocchi VF, Zammit PS (2010). Dynamics of muscle fibre growth during postnatal mouse development. BMC Dev Biol.

[27] Duddy W, Duguez S, Johnston H, Cohen TV, Phadke A, Gordish-Dressman H, Nagaraju K, Gnocchi V, Low S, Partridge T (2015). Muscular dystrophy in the mdx mouse is a severe myopathy compounded by hypotrophy, hypertrophy and hyperplasia. Skelet Muscle.

[28] Grounds MD, McGeachie JK (1989). A comparison of muscle precursor replication in crush-injured skeletal muscle of Swiss and BALBc mice. Cell Tissue Res.

[29] Rai M, Nongthomba U, Grounds MD (2014). Skeletal muscle degeneration and regeneration in mice and flies. Curr Top Dev Biol.

[30] Perry RL, Rudnick MA (2000). Molecular mechanisms regulating myogenic determination and differentiation. Front Biosci.

[31] Buckingham M (2007). Skeletal muscle progenitor cells and the role of Pax genes. C R Biol.

[32] Buckingham M, Relaix F (2015). PAX3 and PAX7 as upstream regulators of myogenesis. Semin Cell Dev Biol.

[33] Güller I, Russell AP (2010). MicroRNAs in skeletal muscle: their role and regulation in development, disease and function. J Physiol.

[34] Rao PK, Kumar RM, Farkhondeh M, Baskerville S, Lodish HF (2006). Myogenic factors that regulate expression of muscle-specific microRNAs. Proc Natl Acad Sci U S A.

[35] Rosenberg MI, Georges SA, Asawachaicharn A, Analau E, Tapscott SJ (2006). MyoD inhibits Fstl1 and Utrn expression by inducing transcription of miR-206. J Cell Biol.

[36] Chen J-F, Mandel EM, Thomson JM, Wu Q, Callis TE, Hammond SM, Conlon FL, Wang D-Z (2006). The role of microRNA-1 and microRNA-133 in skeletal muscle proliferation and differentiation. Nat Genet.

[37] Alonso-Martin S, Rochat A, Mademtzoglou D, Morais J, de Reynies A, Aurade F, Chang TH, Zammit PS, Relaix F (2016). Gene expression profiling of muscle stem cells identifies novel regulators of postnatal myogenesis. Front Cell Dev Biol.

[38] Mestdagh P, Van Vlierberghe P, De Weer A, Muth D, Westermann F, Speleman F, Vandesompele J (2009). A novel and universal method for microRNA RT-qPCR data normalization. Genome Biol.

[39] D’Haene B, Mestdagh P, Hellemans J, Vandesompele J (2012). miRNA expression profiling: from reference genes to global mean normalization. Methods Mol Biol.

[40] Payne RW, Murray DA, Harding SA, Baird DB, Soutar DM. GenStat for Windows (12th Edition) Introduction. Hemel Hempstead: VSN International; 2009.

[41] Callis TE, Deng Z, Chen J-F, Wang D-Z (2008). Muscling through the microRNA world. Exp Biol Med.

[42] Mei Q, Li X, Meng Y, Wu Z, Guo M, Zhao Y, Fu X, Han W (2012). A facile and specific assay for quantifying microRNA by an optimized RT-qPCR approach. PLoS One.

[43] Mestdagh P, Hartmann N, Baeriswyl L, Andreasen D, Bernard N, Chen C, Cheo D, D’Andrade P, DeMayo M, Dennis L (2014). Evaluation of quantitative miRNA expression platforms in the microRNA quality control (miRQC) study. Nat Methods.

[44] Brattelid T, Aarnes EK, Helgeland E, Guvaag S, Eichele H, Jonassen AK (2011). Normalization strategy is critical for the outcome of miRNA expression analyses in the rat heart. Physiol Genomics.

[45] Meyer SU, Kaiser S, Wagner C, Thirion C, Pfaffl MW (2012). Profound effect of profiling platform and normalization strategy on detection of differentially expressed microRNAs--a comparative study. PLoS One.

[46] Sood P, Krek A, Zavolan M, Macino G, Rajewsky N (2006). Cell-type-specific signatures of microRNAs on target mRNA expression. Proc Natl Acad Sci U S A.

[47] McCarthy JJ, Esser KA (2007). MicroRNA-1 and microRNA-133a expression are decreased during skeletal muscle hypertrophy. J Appl Physiol.

[48] Chen J-F, Tao Y, Li J, Deng Z, Yan Z, Xiao X, Wang D-Z (2010). microRNA-1 and microRNA-206 regulate skeletal muscle satellite cell proliferation and differentiation by repressing Pax7. J Cell Biol.

[49] Chapman MA, Meza R, Lieber RL: Skeletal muscle fibroblasts in health and disease. Differentiation; research in biological diversity 2016;92(3):108–15. 10.1016/j.diff.2016.05.007 PMC5079803 27282924

[50] Bruusgaard JC, Gundersen K (2008). In vivo time-lapse microscopy reveals no loss of murine myonuclei during weeks of muscle atrophy. J Clin Invest.

[51] Schmalbruch H, Hellhammer U (1977). The number of nuclei in adult rat muscles with special reference to satellite cells. Anat Rec.

[52] Kirby TJ, Patel RM, McClintock TS, Dupont-Versteegden EE, Peterson CA, McCarthy JJ (2016). Myonuclear transcription is responsive to mechanical load and DNA content but uncoupled from cell size during hypertrophy. Mol Biol Cell.

[53] Chikenji A, Ando H, Nariyama M, Suga T, Iida R, Gomi K (2016). MyoD is regulated by the miR-29a-Tet1 pathway in C2C12 myoblast cells. J Oral Sci.

[54] Hu Z, Klein JD, Mitch WE, Zhang L, Martinez I, Wang XH (2014). MicroRNA-29 induces cellular senescence in aging muscle through multiple signaling pathways. Aging (Albany NY).

[55] Wei W, He HB, Zhang WY, Zhang HX, Bai JB, Liu HZ, Cao JH, Chang KC, Li XY, Zhao SH (2013). miR-29 targets Akt3 to reduce proliferation and facilitate differentiation of myoblasts in skeletal muscle development. Cell Death Dis.

[56] Alexander MS, Kawahara G, Motohashi N, Casar JC, Eisenberg I, Myers JA, Gasperini MJ, Estrella EA, Kho AT, Mitsuhashi S (2013). MicroRNA-199a is induced in dystrophic muscle and affects WNT signaling, cell proliferation, and myogenic differentiation. Cell Death Differ.

[57] Greco S, Simone MD, Colussi C, Zaccagnini G, Fasanaro P, Pescatori M, Cardani R, Perbellini R, Isaia E, Sale P (2009). Common micro-RNA signature in skeletal muscle damage and regeneration induced by Duchenne muscular dystrophy and acute ischemia. FASEB J.

[58] Russell AP, Wada S, Vergani L, Hock MB, Lamon S, Leger B, Ushida T, Cartoni R, Wadley GD, Hespel P (2012). Disruption of skeletal muscle mitochondrial network genes and miRNAs in amyotrophic lateral sclerosis. Neurobiol Dis.

[59] Liu X, Cheng Y, Chen X, Yang J, Xu L, Zhang C (2011). MicroRNA-31 regulated by the extracellular regulated kinase is involved in vascular smooth muscle cell growth via large tumor suppressor homolog 2. J Biol Chem.

[60] Russell AP, Lamon S, Boon H, Wada S, Guller I, Brown EL, Chibalin AV, Zierath J, Snow RJ, Stepto NK et al.: Regulation of miRNAs in human skeletal muscle following acute endurance exercise and short term endurance training. The Journal of physiology 2013;591(18):4637–53. 10.1113/jphysiol.2013.255695 PMC3784204 23798494

[61] Cacchiarelli D, Incitti T, Martone J, Cesana M, Cazzella V, Santini T, Sthandier O, Bozzoni I (2011). miR-31 modulates dystrophin expression: new implications for duchenne muscular dystrophy therapy. EMBO Rep.

[62] Ichihara A, Wang Z, Jinnin M, Izuno Y, Shimozono N, Yamane K, Fujisawa A, Moriya C, Fukushima S, Inoue Y (2014). Upregulation of miR-18a-5p contributes to epidermal necrolysis in severe drug eruptions. J Allergy Clin Immunol.

[63] Kee HJ, Kim GR, Cho S-N, Kwon J-S, Ahn Y, Kook H, Jeong MH (2014). miR-18a-5p MicroRNA increases vascular smooth muscle cell differentiation by downregulating Syndecan4. Kor Circulation J.

[64] Peng Y, Xiang H, Chen C, Zheng R, Chai J, Peng J, Jiang S (2013). MiR-224 impairs adipocyte early differentiation and regulates fatty acid metabolism. Int J Biochem Cell Biol.

[65] Kim JY, Park YK, Lee KP, Lee SM, Kang TW, Kim HJ, Dho SH, Kim SY, Kwon KS (2014). Genome-wide profiling of the microRNA-mRNA regulatory network in skeletal muscle with aging. Aging (Albany NY).

[66] Zhou B, Liu HL, Shi FX, Wang JY (2010). MicroRNA expression profiles of porcine skeletal muscle. Anim Genet.

[67] Juan AH, Kumar RM, Marx JG, Young RA, Sartorelli V (2009). Mir-214-dependent regulation of the polycomb protein Ezh2 in skeletal muscle and embryonic stem cells. Mol Cell.

[68] Liu J, Luo XJ, Xiong AW, Zhang ZD, Yue S, Zhu MS, Cheng SY (2010). MicroRNA-214 promotes myogenic differentiation by facilitating exit from mitosis via down-regulation of proto-oncogene N-ras. J Biol Chem.

[69] Basu U, Lozynska O, Moorwood C, Patel G, Wilton SD, Khurana TS (2011). Translational regulation of utrophin by miRNAs. PLoS One.

[70] Perkins KJ, Davies KE (2002). The role of utrophin in the potential therapy of duchenne muscular dystrophy. Neuromuscular Dis NMD.

[71] Tay YM, Tam WL, Ang YS, Gaughwin PM, Yang H, Wang W, Liu R, George J, Ng HH, Perera RJ (2008). MicroRNA-134 modulates the differentiation of mouse embryonic stem cells, where it causes post-transcriptional attenuation of Nanog and LRH1. Stem Cells.

[72] Gaughwin P, Ciesla M, Yang H, Lim B, Brundin P (2011). Stage-specific modulation of cortical neuronal development by Mmu-miR-134. Cereb Cortex.

[73] Chen Y, Melton DW, Gelfond JA, McManus LM, Shireman PK (2012). MiR-351 transiently increases during muscle regeneration and promotes progenitor cell proliferation and survival upon differentiation. Physiol Genomics.

[74] Wang J, Conboy I (2010). Embryonic vs. adult myogenesis: challenging the ‘regeneration recapitulates development’ paradigm. J Mol Cell Biol.

