# Supplementary material for: MicroRNA expression patterns in post-natal mouse skeletal muscle development
Source: BMC Genomics. 2017 Jan 7;18:52. doi: 10.1186/s12864-016-3399-2 (PMC5219731; doi:10.1186/s12864-016-3399-2)
Supplement: Additional file 1: — Descriptive analysis of all Ct values over time. (PDF 349 kb) [file 12864_2016_3399_MOESM1_ESM.pdf]

Analysis Variable : ct				
micro_rna	time_group	N Obs	Mean	Median
ath-miR159a-4373390	2 days	4	30.76	30.76
	2 weeks	5	.	.
	4 weeks	5	.	.
	12 weeks	6	.	.
hsa-let-7f-1#-002417	2 days	4	21.91	21.91
	2 weeks	5	35.26	35.26
	4 weeks	5	36.00	35.89
	12 weeks	6	33.04	33.04
hsa-miR-106b#-002380	2 days	4	29.57	29.42
	2 weeks	5	30.46	30.42
	4 weeks	5	31.87	31.92
	12 weeks	6	33.53	33.59
hsa-miR-124#-002197	2 days	4	32.34	32.34
	2 weeks	5	.	.
	4 weeks	5	25.64	25.64
	12 weeks	6	.	.
hsa-miR-136#-002100	2 days	4	25.26	25.26
	2 weeks	5	26.99	26.41
	4 weeks	5	27.50	27.42
	12 weeks	6	29.99	30.08
hsa-miR-140-3p-002234	2 days	4	27.44	27.13
	2 weeks	5	28.76	27.98
	4 weeks	5	28.73	28.67
	12 weeks	6	28.97	28.87
hsa-miR-143-000466	2 days	4	32.73	32.81
	2 weeks	5	32.26	32.39
	4 weeks	5	31.55	31.28
	12 weeks	6	31.02	31.02
hsa-miR-149-002255	2 days	4	24.27	24.56
	2 weeks	5	25.35	24.93
	4 weeks	5	24.76	24.82
	12 weeks	6	24.25	24.22
hsa-miR-151-5P-002642	2 days	4	30.19	30.24
	2 weeks	5	29.52	28.98
	4 weeks	5	29.43	29.66
	12 weeks	6	29.10	29.05
hsa-miR-15b#-002173	2 days	4	29.46	29.50
	2 weeks	5	31.57	31.35
	4 weeks	5	32.19	31.76
	12 weeks	6	33.88	33.89
hsa-miR-196a-241070_mat	2 days	4	24.14	24.02
	2 weeks	5	26.17	25.69
	4 weeks	5	25.59	25.75
	12 weeks	6	24.90	24.95
hsa-miR-206-000510	2 days	4	18.77	18.71
	2 weeks	5	21.47	20.87

	4 weeks	5	21.98	22.05
	12 weeks	6	24.16	24.04
hsa-miR-213-000516	2 days	4	31.35	31.22
	2 weeks	5	31.66	31.39
	4 weeks	5	30.52	30.58
	12 weeks	6	29.91	29.97
hsa-miR-214#-002293	2 days	4	27.50	27.52
	2 weeks	5	29.87	29.50
	4 weeks	5	30.10	30.15
	12 weeks	6	31.14	31.20
hsa-miR-214-000517	2 days	4	23.99	23.95
	2 weeks	5	27.47	27.11
	4 weeks	5	27.67	27.77
	12 weeks	6	27.63	27.58
hsa-miR-22#-002301	2 days	4	31.46	31.56
	2 weeks	5	30.17	29.52
	4 weeks	5	28.89	28.92
	12 weeks	6	28.23	28.23
hsa-miR-22-000398	2 days	4	28.27	28.44
	2 weeks	5	26.35	26.44
	4 weeks	5	25.54	25.43
	12 weeks	6	25.03	25.05
hsa-miR-223-000526	2 days	4	24.79	24.77
	2 weeks	5	25.91	25.14
	4 weeks	5	24.19	24.25
	12 weeks	6	22.98	22.95
hsa-miR-27a#-002445	2 days	4	30.97	30.80
	2 weeks	5	32.83	32.85
	4 weeks	5	32.80	32.99
	12 weeks	6	32.92	32.66
hsa-miR-299-5p-000600	2 days	4	26.35	26.11
	2 weeks	5	27.15	26.96
	4 weeks	5	28.08	28.03
	12 weeks	6	30.54	30.55
hsa-miR-30a-3p-000416	2 days	4	25.13	25.02
	2 weeks	5	25.71	25.00
	4 weeks	5	24.67	24.61
	12 weeks	6	23.48	23.52
hsa-miR-30c-1#-002108	2 days	4	.	.
	2 weeks	5	33.21	33.21
	4 weeks	5	28.88	32.20
	12 weeks	6	34.88	34.88
hsa-miR-30d#-002305	2 days	4	30.61	30.37
	2 weeks	5	32.69	31.76
	4 weeks	5	30.98	31.02
	12 weeks	6	31.54	31.78
hsa-miR-30e-3p-000422	2 days	4	25.22	25.23
	2 weeks	5	25.70	25.18

	4 weeks	5	24.68	24.63
	12 weeks	6	23.67	23.52
hsa-miR-324-3p-000579	2 days	4	27.02	26.95
	2 weeks	5	29.42	28.56
	4 weeks	5	29.14	29.01
	12 weeks	6	28.70	28.57
hsa-miR-338-5P-002658	2 days	4	31.13	31.15
	2 weeks	5	32.18	31.65
	4 weeks	5	32.01	32.15
	12 weeks	6	32.43	32.24
hsa-miR-363#-001283	2 days	4	.	.
	2 weeks	5	.	.
	4 weeks	5	36.07	36.07
	12 weeks	6	21.24	21.24
hsa-miR-376a#-001287	2 days	4	28.55	28.56
	2 weeks	5	30.68	29.80
	4 weeks	5	31.13	31.14
	12 weeks	6	34.69	33.94
hsa-miR-378-000567	2 days	4	27.97	27.96
	2 weeks	5	28.08	27.65
	4 weeks	5	27.73	27.66
	12 weeks	6	27.31	27.30
hsa-miR-411#-002238	2 days	4	28.06	27.99
	2 weeks	5	31.77	31.25
	4 weeks	5	32.50	32.11
	12 weeks	6	34.84	34.24
hsa-miR-412-001023	2 days	4	28.64	28.74
	2 weeks	5	31.91	31.75
	4 weeks	5	33.82	34.03
	12 weeks	6	35.59	35.59
hsa-miR-421-002700	2 days	4	28.38	28.30
	2 weeks	5	30.75	30.10
	4 weeks	5	31.29	31.35
	12 weeks	6	31.99	31.82
hsa-miR-423-3P-002626	2 days	4	28.46	28.40
	2 weeks	5	29.33	29.56
	4 weeks	5	29.24	28.83
	12 weeks	6	29.02	28.97
hsa-miR-455-001280	2 days	4	28.71	28.80
	2 weeks	5	31.02	30.27
	4 weeks	5	30.73	30.47
	12 weeks	6	32.14	32.07
hsa-miR-485-5p-001036	2 days	4	29.54	29.03
	2 weeks	5	33.18	33.18
	4 weeks	5	.	.
	12 weeks	6	.	.
hsa-miR-493-3p-001282	2 days	4	28.73	28.69
	2 weeks	5	30.82	30.74

	**4 weeks**	**5**	32.43	32.22
	**12 weeks**	**6**	35.55	37.16
**hsa-miR-93#-002139**	**2 days**	**4**	27.46	27.18
	**2 weeks**	**5**	29.07	28.46
	**4 weeks**	**5**	28.89	28.96
	**12 weeks**	**6**	28.48	28.61
**mmu-let-7a#-002478**	**2 days**	**4**	32.01	32.24
	**2 weeks**	**5**	31.74	31.06
	**4 weeks**	**5**	31.46	31.46
	**12 weeks**	**6**	31.58	31.53
**mmu-let-7a-4373169**	**2 days**	**4**	28.10	27.70
	**2 weeks**	**5**	28.55	28.34
	**4 weeks**	**5**	28.33	27.98
	**12 weeks**	**6**	28.21	28.14
**mmu-let-7b-4373168**	**2 days**	**4**	27.54	27.68
	**2 weeks**	**5**	27.52	27.58
	**4 weeks**	**5**	27.29	27.19
	**12 weeks**	**6**	27.58	27.59
**mmu-let-7c-4373167**	**2 days**	**4**	26.06	26.12
	**2 weeks**	**5**	26.33	26.01
	**4 weeks**	**5**	26.02	25.87
	**12 weeks**	**6**	26.40	26.42
**mmu-let-7d-4395394**	**2 days**	**4**	27.00	26.23
	**2 weeks**	**5**	27.10	26.98
	**4 weeks**	**5**	26.59	26.32
	**12 weeks**	**6**	26.38	26.30
**mmu-let-7e-4395517**	**2 days**	**4**	24.31	24.89
	**2 weeks**	**5**	24.69	24.97
	**4 weeks**	**5**	25.72	25.99
	**12 weeks**	**6**	25.69	26.04
**mmu-let-7f-4373164**	**2 days**	**4**	29.88	30.21
	**2 weeks**	**5**	30.44	30.17
	**4 weeks**	**5**	29.75	29.31
	**12 weeks**	**6**	29.41	29.55
**mmu-let-7g-4395393**	**2 days**	**4**	26.61	26.59
	**2 weeks**	**5**	27.41	27.06
	**4 weeks**	**5**	26.39	26.18
	**12 weeks**	**6**	26.36	26.31
**mmu-let-7i-4395332**	**2 days**	**4**	25.79	25.81
	**2 weeks**	**5**	26.70	26.16
	**4 weeks**	**5**	26.52	26.52
	**12 weeks**	**6**	26.38	26.36
**mmu-miR-1-4395333**	**2 days**	**4**	21.59	21.56
	**2 weeks**	**5**	20.71	21.17
	**4 weeks**	**5**	20.78	20.69
	**12 weeks**	**6**	20.12	20.03
**mmu-miR-100-4373160**	**2 days**	**4**	29.39	30.44
	**2 weeks**	**5**	30.76	30.82

	**4 weeks**	**5**	28.95	28.28
	**12 weeks**	**6**	30.10	29.81
**mmu-miR-101a-4395364**	**2 days**	**4**	30.57	30.57
	**2 weeks**	**5**	30.76	29.93
	**4 weeks**	**5**	29.40	29.47
	**12 weeks**	**6**	28.70	28.70
**mmu-miR-101b-002531**	**2 days**	**4**	29.05	29.07
	**2 weeks**	**5**	28.99	28.42
	**4 weeks**	**5**	27.93	27.97
	**12 weeks**	**6**	27.75	27.65
**mmu-miR-103-4373158**	**2 days**	**4**	29.76	29.59
	**2 weeks**	**5**	29.91	29.68
	**4 weeks**	**5**	29.75	29.79
	**12 weeks**	**6**	29.49	29.43
**mmu-miR-106a-4395589**	**2 days**	**4**	24.60	24.69
	**2 weeks**	**5**	26.53	25.89
	**4 weeks**	**5**	26.31	26.32
	**12 weeks**	**6**	26.35	26.37
**mmu-miR-106b-4373155**	**2 days**	**4**	27.95	29.12
	**2 weeks**	**5**	30.00	29.38
	**4 weeks**	**5**	29.32	29.34
	**12 weeks**	**6**	29.25	29.38
**mmu-miR-10b-4395329**	**2 days**	**4**	28.57	28.51
	**2 weeks**	**5**	28.45	27.88
	**4 weeks**	**5**	28.24	28.34
	**12 weeks**	**6**	27.09	27.15
**mmu-miR-1193-002794**	**2 days**	**4**	27.86	27.63
	**2 weeks**	**5**	29.53	29.04
	**4 weeks**	**5**	30.83	30.73
	**12 weeks**	**6**	33.52	33.31
**mmu-miR-1198-002780**	**2 days**	**4**	30.93	30.88
	**2 weeks**	**5**	32.84	32.81
	**4 weeks**	**5**	31.57	31.59
	**12 weeks**	**6**	31.45	31.20
**mmu-miR-122-4395356**	**2 days**	**4**	29.32	29.32
	**2 weeks**	**5**	.	.
	**4 weeks**	**5**	.	.
	**12 weeks**	**6**	35.59	35.59
**mmu-miR-125a-5p-4395309**	**2 days**	**4**	26.66	26.96
	**2 weeks**	**5**	28.23	27.57
	**4 weeks**	**5**	28.19	28.21
	**12 weeks**	**6**	28.24	28.16
**mmu-miR-125b-5p-4373148**	**2 days**	**4**	24.94	25.07
	**2 weeks**	**5**	25.63	25.39
	**4 weeks**	**5**	25.10	25.18
	**12 weeks**	**6**	25.04	25.22
**mmu-miR-126-3p-4395339**	**2 days**	**4**	22.71	22.57
	**2 weeks**	**5**	22.39	22.01

	4 weeks	5	22.16	22.22
	12 weeks	6	21.63	21.61
mmu-miR-126-5p-4373269	2 days	4	27.23	26.92
	2 weeks	5	27.20	26.67
	4 weeks	5	26.66	26.51
	12 weeks	6	26.01	26.04
mmu-miR-127-4373147	2 days	4	20.96	20.97
	2 weeks	5	23.45	23.03
	4 weeks	5	25.07	25.06
	12 weeks	6	28.01	28.00
mmu-miR-1274a-121150_mat	2 days	4	22.89	22.90
	2 weeks	5	25.65	25.28
	4 weeks	5	24.75	24.57
	12 weeks	6	24.59	24.66
mmu-miR-128a-4395327	2 days	4	28.99	29.03
	2 weeks	5	30.32	30.01
	4 weeks	5	30.43	30.41
	12 weeks	6	29.84	29.85
mmu-miR-129-5p-4373171	2 days	4	23.33	23.33
	2 weeks	5	.	.
	4 weeks	5	.	.
	12 weeks	6	.	.
mmu-miR-130a-4373145	2 days	4	28.20	28.28
	2 weeks	5	29.46	28.95
	4 weeks	5	30.13	30.08
	12 weeks	6	30.81	30.81
mmu-miR-130b-4373144	2 days	4	28.78	28.62
	2 weeks	5	32.43	31.26
	4 weeks	5	33.24	32.87
	12 weeks	6	34.81	34.39
mmu-miR-132-4373143	2 days	4	28.78	28.73
	2 weeks	5	29.74	29.09
	4 weeks	5	28.48	29.21
	12 weeks	6	28.77	28.78
mmu-miR-133a-4395357	2 days	4	19.24	19.22
	2 weeks	5	19.30	18.82
	4 weeks	5	18.67	18.68
	12 weeks	6	17.50	17.63
mmu-miR-133b-4395358	2 days	4	19.35	19.75
	2 weeks	5	21.13	20.94
	4 weeks	5	19.47	19.98
	12 weeks	6	19.26	19.18
mmu-miR-134-4373299	2 days	4	25.40	24.29
	2 weeks	5	28.41	27.76
	4 weeks	5	29.23	29.16
	12 weeks	6	31.42	31.33
mmu-miR-136-4395641	2 days	4	24.96	24.86
	2 weeks	5	26.08	25.65

	**4 weeks**	**5**	26.40	26.29
	**12 weeks**	**6**	28.99	29.14
**mmu-miR-138-4395395**	**2 days**	**4**	29.66	29.54
	**2 weeks**	**5**	29.90	29.51
	**4 weeks**	**5**	30.13	30.14
	**12 weeks**	**6**	30.28	30.31
**mmu-miR-139-5p-4395400**	**2 days**	**4**	25.86	25.23
	**2 weeks**	**5**	26.15	25.66
	**4 weeks**	**5**	25.97	26.05
	**12 weeks**	**6**	25.43	25.42
**mmu-miR-140-4373374**	**2 days**	**4**	25.96	25.32
	**2 weeks**	**5**	26.86	26.03
	**4 weeks**	**5**	26.81	26.77
	**12 weeks**	**6**	27.02	27.09
**mmu-miR-141#-002513**	**2 days**	**4**	32.48	32.86
	**2 weeks**	**5**	31.29	31.11
	**4 weeks**	**5**	31.37	31.41
	**12 weeks**	**6**	31.92	31.90
**mmu-miR-142-3p-4373136**	**2 days**	**4**	29.74	29.83
	**2 weeks**	**5**	30.60	30.00
	**4 weeks**	**5**	29.43	29.46
	**12 weeks**	**6**	28.94	28.84
**mmu-miR-143-4395360**	**2 days**	**4**	27.55	27.37
	**2 weeks**	**5**	27.81	27.15
	**4 weeks**	**5**	26.86	26.71
	**12 weeks**	**6**	26.12	26.07
**mmu-miR-145-4395389**	**2 days**	**4**	24.37	24.35
	**2 weeks**	**5**	24.54	24.08
	**4 weeks**	**5**	23.92	23.94
	**12 weeks**	**6**	22.92	23.01
**mmu-miR-146a-4373132**	**2 days**	**4**	30.50	27.99
	**2 weeks**	**5**	27.69	27.10
	**4 weeks**	**5**	27.11	27.22
	**12 weeks**	**6**	26.08	26.14
**mmu-miR-146b-4373178**	**2 days**	**4**	30.85	31.07
	**2 weeks**	**5**	32.04	32.18
	**4 weeks**	**5**	31.40	31.29
	**12 weeks**	**6**	30.04	29.69
**mmu-miR-147-4395373**	**2 days**	**4**	30.51	30.51
	**2 weeks**	**5**	.	.
	**4 weeks**	**5**	.	.
	**12 weeks**	**6**	.	.
**mmu-miR-148a-4373130**	**2 days**	**4**	28.67	27.87
	**2 weeks**	**5**	29.10	28.62
	**4 weeks**	**5**	29.03	29.13
	**12 weeks**	**6**	28.96	29.04
**mmu-miR-150-4373127**	**2 days**	**4**	29.28	28.41
	**2 weeks**	**5**	27.49	26.94

	**4 weeks**	**5**	26.44	26.41
	**12 weeks**	**6**	25.50	25.48
**mmu-miR-151-3p-4373304**	**2 days**	**4**	29.26	29.08
	**2 weeks**	**5**	30.66	30.57
	**4 weeks**	**5**	32.46	31.96
	**12 weeks**	**6**	29.99	29.80
**mmu-miR-152-4395170**	**2 days**	**4**	27.27	26.42
	**2 weeks**	**5**	28.14	27.65
	**4 weeks**	**5**	27.88	28.00
	**12 weeks**	**6**	27.83	28.02
**mmu-miR-153-4373305**	**2 days**	**4**	31.81	31.81
	**2 weeks**	**5**	.	.
	**4 weeks**	**5**	.	.
	**12 weeks**	**6**	.	.
**mmu-miR-155-4395701**	**2 days**	**4**	31.28	31.61
	**2 weeks**	**5**	32.29	31.12
	**4 weeks**	**5**	30.37	30.40
	**12 weeks**	**6**	29.35	29.29
**mmu-miR-15a#-002488**	**2 days**	**4**	30.39	30.46
	**2 weeks**	**5**	31.33	31.23
	**4 weeks**	**5**	31.57	31.56
	**12 weeks**	**6**	32.11	31.99
**mmu-miR-15a-4373123**	**2 days**	**4**	28.96	30.63
	**2 weeks**	**5**	29.92	29.77
	**4 weeks**	**5**	27.93	27.33
	**12 weeks**	**6**	28.88	29.11
**mmu-miR-15b-4373122**	**2 days**	**4**	29.89	29.60
	**2 weeks**	**5**	30.14	29.65
	**4 weeks**	**5**	29.69	29.83
	**12 weeks**	**6**	30.24	30.21
**mmu-miR-16-4373121**	**2 days**	**4**	25.57	24.35
	**2 weeks**	**5**	24.73	24.47
	**4 weeks**	**5**	24.51	24.57
	**12 weeks**	**6**	23.48	23.64
**mmu-miR-17-4395419**	**2 days**	**4**	27.26	24.55
	**2 weeks**	**5**	26.29	25.62
	**4 weeks**	**5**	25.87	25.89
	**12 weeks**	**6**	26.05	26.07
**mmu-miR-181a-4373117**	**2 days**	**4**	28.27	27.93
	**2 weeks**	**5**	29.33	29.19
	**4 weeks**	**5**	28.78	28.67
	**12 weeks**	**6**	28.91	29.02
**mmu-miR-1839-3p-121203_mat**	**2 days**	**4**	29.64	29.61
	**2 weeks**	**5**	30.63	30.62
	**4 weeks**	**5**	31.15	30.99
	**12 weeks**	**6**	31.25	31.22
**mmu-miR-1839-5p-121135_mat**	**2 days**	**4**	30.83	30.56
	**2 weeks**	**5**	31.11	30.75

	4 weeks	5	31.22	31.27
	12 weeks	6	31.18	31.08
mmu-miR-185-4395382	2 days	4	31.65	31.48
	2 weeks	5	33.22	33.46
	4 weeks	5	31.99	31.81
	12 weeks	6	30.77	30.88
mmu-miR-186-4395396	2 days	4	30.06	28.17
	2 weeks	5	30.57	29.81
	4 weeks	5	29.40	29.47
	12 weeks	6	28.43	28.30
mmu-miR-188-3p-4395217	2 days	4	24.88	24.88
	2 weeks	5	.	.
	4 weeks	5	34.44	34.44
	12 weeks	6	39.08	39.08
mmu-miR-188-5p-4395431	2 days	4	28.90	29.46
	2 weeks	5	31.28	31.25
	4 weeks	5	30.97	30.76
	12 weeks	6	32.25	32.21
mmu-miR-1896-121128_mat	2 days	4	31.88	31.91
	2 weeks	5	30.97	30.80
	4 weeks	5	31.05	30.98
	12 weeks	6	32.26	32.25
mmu-miR-1897-5p-121199_mat	2 days	4	31.30	31.29
	2 weeks	5	31.20	31.13
	4 weeks	5	31.10	31.26
	12 weeks	6	31.12	30.84
mmu-miR-18a-4395533	2 days	4	29.70	29.87
	2 weeks	5	31.92	31.44
	4 weeks	5	31.59	31.60
	12 weeks	6	33.15	33.21
mmu-miR-18b-4395596	2 days	4	30.32	30.32
	2 weeks	5	.	.
	4 weeks	5	.	.
	12 weeks	6	.	.
mmu-miR-1904-121162_mat	2 days	4	27.28	27.23
	2 weeks	5	28.04	28.17
	4 weeks	5	27.16	27.60
	12 weeks	6	27.46	27.33
mmu-miR-1905-121196_mat	2 days	4	30.85	31.00
	2 weeks	5	30.46	30.54
	4 weeks	5	29.60	29.42
	12 weeks	6	31.87	31.53
mmu-miR-191-4395410	2 days	4	24.23	23.87
	2 weeks	5	25.35	24.66
	4 weeks	5	24.63	24.65
	12 weeks	6	24.09	23.97
mmu-miR-192-4373108	2 days	4	31.48	31.56
	2 weeks	5	31.82	31.76

	4 weeks	5	31.19	31.24
	12 weeks	6	31.09	31.13
mmu-miR-193#-002577	2 days	4	31.42	31.53
	2 weeks	5	31.26	30.78
	4 weeks	5	30.74	30.81
	12 weeks	6	30.13	30.13
mmu-miR-1937b-241023_mat	2 days	4	18.68	18.46
	2 weeks	5	21.77	21.08
	4 weeks	5	21.55	21.50
	12 weeks	6	20.82	20.71
mmu-miR-1937c-241011_mat	2 days	4	20.94	20.92
	2 weeks	5	23.98	23.61
	4 weeks	5	23.83	23.89
	12 weeks	6	23.10	23.05
mmu-miR-1939-121180_mat	2 days	4	29.66	29.67
	2 weeks	5	32.05	32.26
	4 weeks	5	32.61	32.22
	12 weeks	6	32.71	32.81
mmu-miR-193b-4395597	2 days	4	29.32	27.46
	2 weeks	5	27.11	26.70
	4 weeks	5	26.92	26.94
	12 weeks	6	25.63	25.65
mmu-miR-194-4373106	2 days	4	28.70	29.79
	2 weeks	5	31.59	31.78
	4 weeks	5	30.95	31.09
	12 weeks	6	29.96	30.20
mmu-miR-1941-3p-121130_mat	2 days	4	36.93	36.93
	2 weeks	5	26.66	26.66
	4 weeks	5	.	.
	12 weeks	6	33.81	34.00
mmu-miR-1944-121189_mat	2 days	4	30.29	30.35
	2 weeks	5	31.90	31.39
	4 weeks	5	30.21	30.28
	12 weeks	6	31.68	31.66
mmu-miR-195-4373105	2 days	4	29.73	29.77
	2 weeks	5	27.80	27.50
	4 weeks	5	26.95	26.87
	12 weeks	6	26.18	26.34
mmu-miR-1951-121165_mat	2 days	4	22.81	22.47
	2 weeks	5	22.50	22.30
	4 weeks	5	22.30	22.94
	12 weeks	6	21.91	22.01
mmu-miR-1952-121167_mat	2 days	4	13.45	13.45
	2 weeks	5	.	.
	4 weeks	5	.	.
	12 weeks	6	.	.
mmu-miR-1961-197391_mat	2 days	4	29.40	29.24
	2 weeks	5	29.87	29.55

	4 weeks	5	29.08	29.33
	12 weeks	6	29.62	29.51
mmu-miR-196b-4395326	2 days	4	28.72	27.79
	2 weeks	5	29.66	29.00
	4 weeks	5	29.97	30.02
	12 weeks	6	29.56	29.56
mmu-miR-1971-121161_mat	2 days	4	28.83	28.69
	2 weeks	5	30.34	29.99
	4 weeks	5	29.65	29.98
	12 weeks	6	30.38	30.44
mmu-miR-1981-121200_mat	2 days	4	29.69	29.51
	2 weeks	5	32.56	32.73
	4 weeks	5	33.35	33.44
	12 weeks	6	32.00	32.01
mmu-miR-199a-3p-4395415	2 days	4	24.89	23.54
	2 weeks	5	25.58	25.13
	4 weeks	5	25.39	25.37
	12 weeks	6	25.61	25.71
mmu-miR-199a-5p-4373272	2 days	4	28.17	30.19
	2 weeks	5	33.84	33.84
	4 weeks	5	34.20	32.76
	12 weeks	6	33.40	33.58
mmu-miR-19a-4373099	2 days	4	25.51	25.67
	2 weeks	5	28.16	27.69
	4 weeks	5	27.65	27.62
	12 weeks	6	28.03	28.13
mmu-miR-19b-4373098	2 days	4	26.01	22.93
	2 weeks	5	24.40	23.89
	4 weeks	5	24.11	24.13
	12 weeks	6	24.03	24.27
mmu-miR-200a-4378069	2 days	4	29.77	32.01
	2 weeks	5	.	.
	4 weeks	5	.	.
	12 weeks	6	33.60	33.60
mmu-miR-200b-4395362	2 days	4	29.17	30.54
	2 weeks	5	32.36	32.36
	4 weeks	5	33.98	33.84
	12 weeks	6	32.90	32.58
mmu-miR-200c-4395411	2 days	4	28.97	29.54
	2 weeks	5	34.19	34.19
	4 weeks	5	34.47	34.36
	12 weeks	6	32.33	32.49
mmu-miR-203-4373095	2 days	4	28.28	28.18
	2 weeks	5	31.68	31.84
	4 weeks	5	30.55	30.44
	12 weeks	6	30.26	30.30
mmu-miR-204-4373094	2 days	4	29.43	29.21
	2 weeks	5	29.20	28.95

	**4 weeks**	**5**	29.06	28.93
	**12 weeks**	**6**	29.07	29.14
**mmu-miR-205-4373093**	**2 days**	**4**	30.80	31.25
	**2 weeks**	**5**	.	.
	**4 weeks**	**5**	.	.
	**12 weeks**	**6**	.	.
**mmu-miR-207-4373314**	**2 days**	**4**	27.86	27.86
	**2 weeks**	**5**	.	.
	**4 weeks**	**5**	.	.
	**12 weeks**	**6**	.	.
**mmu-miR-208-4373091**	**2 days**	**4**	32.56	32.56
	**2 weeks**	**5**	.	.
	**4 weeks**	**5**	.	.
	**12 weeks**	**6**	.	.
**mmu-miR-20a-4373286**	**2 days**	**4**	26.58	26.56
	**2 weeks**	**5**	27.46	26.98
	**4 weeks**	**5**	26.89	26.98
	**12 weeks**	**6**	27.13	27.05
**mmu-miR-21-4373090**	**2 days**	**4**	29.35	27.96
	**2 weeks**	**5**	28.79	28.56
	**4 weeks**	**5**	27.90	27.74
	**12 weeks**	**6**	26.25	26.29
**mmu-miR-210-4373089**	**2 days**	**4**	25.69	27.08
	**2 weeks**	**5**	30.16	30.05
	**4 weeks**	**5**	30.39	30.24
	**12 weeks**	**6**	30.74	30.94
**mmu-miR-211-4373315**	**2 days**	**4**	25.14	25.14
	**2 weeks**	**5**	.	.
	**4 weeks**	**5**	32.35	32.35
	**12 weeks**	**6**	33.89	33.35
**mmu-miR-212-002551**	**2 days**	**4**	30.56	29.87
	**2 weeks**	**5**	30.69	30.71
	**4 weeks**	**5**	31.58	31.22
	**12 weeks**	**6**	30.49	30.31
**mmu-miR-2134-241120_mat**	**2 days**	**4**	17.84	17.98
	**2 weeks**	**5**	17.62	17.13
	**4 weeks**	**5**	17.23	17.08
	**12 weeks**	**6**	17.78	17.81
**mmu-miR-2135-241140_mat**	**2 days**	**4**	26.64	26.78
	**2 weeks**	**5**	26.82	26.29
	**4 weeks**	**5**	26.00	26.08
	**12 weeks**	**6**	27.35	27.32
**mmu-miR-2138-241080_mat**	**2 days**	**4**	24.26	24.46
	**2 weeks**	**5**	22.90	22.36
	**4 weeks**	**5**	22.60	22.63
	**12 weeks**	**6**	23.77	23.85
**mmu-miR-214-4395417**	**2 days**	**4**	26.26	25.24
	**2 weeks**	**5**	28.85	28.26

	4 weeks	5	28.75	28.58
	12 weeks	6	29.04	29.02
mmu-miR-2146-241082_mat	2 days	4	22.70	22.53
	2 weeks	5	22.69	22.02
	4 weeks	5	21.79	21.75
	12 weeks	6	22.35	22.33
mmu-miR-216a-4395331	2 days	4	26.15	26.15
	2 weeks	5	.	.
	4 weeks	5	.	.
	12 weeks	6	.	.
mmu-miR-216b-4395437	2 days	4	29.79	29.79
	2 weeks	5	34.65	34.65
	4 weeks	5	.	.
	12 weeks	6	.	.
mmu-miR-217-001133	2 days	4	27.03	27.03
	2 weeks	5	.	.
	4 weeks	5	34.52	34.52
	12 weeks	6	30.19	30.19
mmu-miR-217-4395686	2 days	4	24.34	24.34
	2 weeks	5	.	.
	4 weeks	5	.	.
	12 weeks	6	.	.
mmu-miR-218-4373081	2 days	4	29.36	29.31
	2 weeks	5	31.09	30.42
	4 weeks	5	31.47	31.39
	12 weeks	6	30.84	30.88
mmu-miR-2182-241119_mat	2 days	4	28.46	28.46
	2 weeks	5	27.69	27.32
	4 weeks	5	27.05	26.97
	12 weeks	6	28.00	28.06
mmu-miR-221-4373077	2 days	4	31.82	31.47
	2 weeks	5	31.20	31.05
	4 weeks	5	29.92	29.92
	12 weeks	6	29.37	29.46
mmu-miR-222-4395387	2 days	4	30.14	29.93
	2 weeks	5	29.45	28.84
	4 weeks	5	28.63	28.64
	12 weeks	6	27.46	27.51
mmu-miR-223-4395406	2 days	4	27.11	27.13
	2 weeks	5	27.73	27.21
	4 weeks	5	26.87	26.87
	12 weeks	6	25.69	25.67
mmu-miR-224-4395683	2 days	4	29.55	29.61
	2 weeks	5	32.07	32.02
	4 weeks	5	33.47	32.98
	12 weeks	6	32.09	31.76
mmu-miR-23b-4373073	2 days	4	30.12	29.97
	2 weeks	5	30.61	30.25

	4 weeks	5	29.97	30.17
	12 weeks	6	30.00	30.03
mmu-miR-24-2#-002494	2 days	4	28.52	28.55
	2 weeks	5	29.91	29.00
	4 weeks	5	29.10	29.23
	12 weeks	6	28.74	28.70
mmu-miR-24-4373072	2 days	4	21.65	21.50
	2 weeks	5	23.03	22.45
	4 weeks	5	22.56	22.50
	12 weeks	6	22.46	22.57
mmu-miR-25-4373071	2 days	4	29.33	29.87
	2 weeks	5	31.01	30.17
	4 weeks	5	31.01	30.93
	12 weeks	6	30.79	30.72
mmu-miR-26a-4395166	2 days	4	24.80	24.68
	2 weeks	5	25.04	24.57
	4 weeks	5	24.30	24.22
	12 weeks	6	23.64	23.66
mmu-miR-26b-4395167	2 days	4	27.77	26.88
	2 weeks	5	27.10	26.52
	4 weeks	5	26.54	26.50
	12 weeks	6	26.03	26.00
mmu-miR-27a-4373287	2 days	4	27.96	27.71
	2 weeks	5	28.83	28.29
	4 weeks	5	28.21	28.22
	12 weeks	6	28.22	28.19
mmu-miR-27b-4373068	2 days	4	28.79	28.69
	2 weeks	5	28.87	28.18
	4 weeks	5	28.45	28.34
	12 weeks	6	28.48	28.37
mmu-miR-28#-002545	2 days	4	28.66	28.55
	2 weeks	5	29.99	29.73
	4 weeks	5	29.69	29.60
	12 weeks	6	28.91	28.95
mmu-miR-28-4373067	2 days	4	31.87	29.48
	2 weeks	5	30.10	29.51
	4 weeks	5	29.64	29.55
	12 weeks	6	29.81	29.74
mmu-miR-293-4386754	2 days	4	27.83	27.83
	2 weeks	5	34.63	34.63
	4 weeks	5	14.62	14.62
	12 weeks	6	35.69	35.69
mmu-miR-296-5p-4373066	2 days	4	26.32	25.78
	2 weeks	5	28.13	27.57
	4 weeks	5	29.03	28.97
	12 weeks	6	31.06	30.80
mmu-miR-297a#-002454	2 days	4	30.44	30.55
	2 weeks	5	33.87	33.92

	4 weeks	5	34.15	34.37
	12 weeks	6	33.48	32.96
mmu-miR-298-4395728	2 days	4	27.19	27.21
	2 weeks	5	31.06	30.34
	4 weeks	5	32.87	32.91
	12 weeks	6	33.52	33.52
mmu-miR-299-002612	2 days	4	28.65	28.50
	2 weeks	5	30.26	29.26
	4 weeks	5	31.01	31.17
	12 weeks	6	33.52	33.46
mmu-miR-29a-4395223	2 days	4	27.63	27.58
	2 weeks	5	26.98	26.25
	4 weeks	5	25.30	25.21
	12 weeks	6	23.08	22.96
mmu-miR-29c-4395171	2 days	4	32.87	32.90
	2 weeks	5	31.53	30.69
	4 weeks	5	29.74	29.87
	12 weeks	6	27.96	27.93
mmu-miR-300-000191	2 days	4	27.82	28.16
	2 weeks	5	31.57	31.98
	4 weeks	5	33.13	33.38
	12 weeks	6	33.42	33.61
mmu-miR-301a-4373064	2 days	4	27.01	27.37
	2 weeks	5	29.23	28.72
	4 weeks	5	29.55	29.40
	12 weeks	6	29.85	29.84
mmu-miR-301b-4395730	2 days	4	27.15	28.70
	2 weeks	5	30.70	30.30
	4 weeks	5	31.12	31.14
	12 weeks	6	32.05	31.96
mmu-miR-302a-4378070	2 days	4	26.53	26.53
	2 weeks	5	.	.
	4 weeks	5	.	.
	12 weeks	6	.	.
mmu-miR-302b-4378071	2 days	4	27.08	27.08
	2 weeks	5	.	.
	4 weeks	5	.	.
	12 weeks	6	.	.
mmu-miR-302c-4395688	2 days	4	30.72	30.72
	2 weeks	5	.	.
	4 weeks	5	.	.
	12 weeks	6	.	.
mmu-miR-302d-4373063	2 days	4	29.23	29.23
	2 weeks	5	.	.
	4 weeks	5	37.49	37.49
	12 weeks	6	.	.
mmu-miR-30a-4373061	2 days	4	27.90	26.35
	2 weeks	5	26.36	25.87

	**4 weeks**	**5**	26.06	26.07
	**12 weeks**	**6**	25.09	25.12
**mmu-miR-30b-4373290**	**2 days**	**4**	26.47	25.12
	**2 weeks**	**5**	25.21	24.85
	**4 weeks**	**5**	24.44	24.32
	**12 weeks**	**6**	23.69	23.68
**mmu-miR-30c-4373060**	**2 days**	**4**	27.44	24.64
	**2 weeks**	**5**	24.42	24.01
	**4 weeks**	**5**	23.61	23.71
	**12 weeks**	**6**	22.14	22.12
**mmu-miR-30d-4373059**	**2 days**	**4**	26.97	27.21
	**2 weeks**	**5**	27.46	26.75
	**4 weeks**	**5**	26.81	26.79
	**12 weeks**	**6**	26.01	26.17
**mmu-miR-30e-4395334**	**2 days**	**4**	24.57	26.02
	**2 weeks**	**5**	26.57	25.98
	**4 weeks**	**5**	25.74	25.85
	**12 weeks**	**6**	24.62	24.66
**mmu-miR-31-4373331**	**2 days**	**4**	27.25	29.92
	**2 weeks**	**5**	32.90	32.95
	**4 weeks**	**5**	33.02	33.36
	**12 weeks**	**6**	33.22	33.02
**mmu-miR-320-4395388**	**2 days**	**4**	27.61	27.68
	**2 weeks**	**5**	29.20	28.93
	**4 weeks**	**5**	29.75	29.83
	**12 weeks**	**6**	28.66	28.74
**mmu-miR-322#-002506**	**2 days**	**4**	25.87	25.82
	**2 weeks**	**5**	29.09	28.36
	**4 weeks**	**5**	29.67	29.79
	**12 weeks**	**6**	31.89	31.89
**mmu-miR-322-001059**	**2 days**	**4**	24.90	24.93
	**2 weeks**	**5**	28.21	27.49
	**4 weeks**	**5**	28.52	28.63
	**12 weeks**	**6**	29.39	29.43
**mmu-miR-322-4378107**	**2 days**	**4**	28.13	27.90
	**2 weeks**	**5**	29.16	28.49
	**4 weeks**	**5**	28.95	28.92
	**12 weeks**	**6**	28.89	28.96
**mmu-miR-323-3p-4395338**	**2 days**	**4**	26.56	25.50
	**2 weeks**	**5**	28.53	27.94
	**4 weeks**	**5**	29.57	29.57
	**12 weeks**	**6**	32.47	31.93
**mmu-miR-324-3p-4395639**	**2 days**	**4**	28.45	29.23
	**2 weeks**	**5**	31.45	31.31
	**4 weeks**	**5**	31.70	31.76
	**12 weeks**	**6**	31.11	31.10
**mmu-miR-325-001060**	**2 days**	**4**	.	.
	**2 weeks**	**5**	.	.

	4 weeks	5	.	.
	12 weeks	6	26.96	26.96
mmu-miR-328-4373049	2 days	4	25.86	26.11
	2 weeks	5	28.47	28.52
	4 weeks	5	28.70	28.75
	12 weeks	6	27.63	27.75
mmu-miR-329-4373336	2 days	4	29.45	29.50
	2 weeks	5	30.77	30.59
	4 weeks	5	32.53	32.73
	12 weeks	6	33.59	33.95
mmu-miR-330-4395341	2 days	4	28.25	28.25
	2 weeks	5	34.69	34.69
	4 weeks	5	.	.
	12 weeks	6	.	.
mmu-miR-331-3p-4373046	2 days	4	28.27	27.31
	2 weeks	5	28.45	27.81
	4 weeks	5	29.39	29.39
	12 weeks	6	27.07	26.97
mmu-miR-335-3p-4395296	2 days	4	26.03	26.65
	2 weeks	5	30.02	29.28
	4 weeks	5	29.91	29.86
	12 weeks	6	31.93	31.82
mmu-miR-335-5p-4373045	2 days	4	26.56	25.53
	2 weeks	5	27.40	27.07
	4 weeks	5	28.26	28.11
	12 weeks	6	30.38	30.38
mmu-miR-337-000193	2 days	4	25.84	25.80
	2 weeks	5	26.69	26.02
	4 weeks	5	27.21	27.31
	12 weeks	6	29.76	29.75
mmu-miR-337-3p-4395662	2 days	4	28.95	28.04
	2 weeks	5	28.95	28.46
	4 weeks	5	29.45	29.49
	12 weeks	6	31.32	31.18
mmu-miR-337-5p-4395645	2 days	4	25.86	26.44
	2 weeks	5	28.19	27.70
	4 weeks	5	28.53	28.47
	12 weeks	6	30.35	30.36
mmu-miR-339-3p-4395663	2 days	4	29.89	29.78
	2 weeks	5	30.94	30.73
	4 weeks	5	31.58	31.45
	12 weeks	6	30.68	30.45
mmu-miR-339-5p-4395368	2 days	4	29.62	30.70
	2 weeks	5	31.76	31.08
	4 weeks	5	31.94	31.68
	12 weeks	6	30.65	30.63
mmu-miR-340-3p-4395370	2 days	4	33.28	31.62
	2 weeks	5	31.92	31.65

	**4 weeks**	**5**	31.96	32.05
	**12 weeks**	**6**	31.17	30.98
**mmu-miR-340-5p-4395369**	**2 days**	**4**	29.19	29.85
	**2 weeks**	**5**	31.06	30.81
	**4 weeks**	**5**	31.11	31.08
	**12 weeks**	**6**	30.23	30.21
**mmu-miR-342-3p-4395371**	**2 days**	**4**	26.23	26.07
	**2 weeks**	**5**	27.66	27.09
	**4 weeks**	**5**	27.70	27.82
	**12 weeks**	**6**	27.73	27.81
**mmu-miR-34a-4395168**	**2 days**	**4**	30.81	30.75
	**2 weeks**	**5**	31.40	30.83
	**4 weeks**	**5**	30.82	30.84
	**12 weeks**	**6**	29.14	29.83
**mmu-miR-34b-3p-4395748**	**2 days**	**4**	31.50	30.45
	**2 weeks**	**5**	31.30	30.82
	**4 weeks**	**5**	31.44	31.39
	**12 weeks**	**6**	31.82	32.29
**mmu-miR-34c#-002584**	**2 days**	**4**	30.59	30.74
	**2 weeks**	**5**	31.89	31.57
	**4 weeks**	**5**	31.31	31.15
	**12 weeks**	**6**	31.81	31.70
**mmu-miR-34c-4373036**	**2 days**	**4**	31.15	31.15
	**2 weeks**	**5**	34.92	34.92
	**4 weeks**	**5**	.	.
	**12 weeks**	**6**	27.95	27.95
**mmu-miR-351-4373345**	**2 days**	**4**	26.98	26.63
	**2 weeks**	**5**	30.42	30.02
	**4 weeks**	**5**	31.14	31.32
	**12 weeks**	**6**	33.86	34.21
**mmu-miR-362-3p-4395746**	**2 days**	**4**	31.08	31.15
	**2 weeks**	**5**	31.92	31.94
	**4 weeks**	**5**	32.06	31.81
	**12 weeks**	**6**	33.36	33.07
**mmu-miR-362-5p-002614**	**2 days**	**4**	30.25	30.24
	**2 weeks**	**5**	31.66	31.23
	**4 weeks**	**5**	31.90	32.00
	**12 weeks**	**6**	32.66	32.82
**mmu-miR-365-4373194**	**2 days**	**4**	28.95	28.20
	**2 weeks**	**5**	27.31	27.17
	**4 weeks**	**5**	27.21	27.17
	**12 weeks**	**6**	25.62	25.69
**mmu-miR-369-5p-4373195**	**2 days**	**4**	28.08	28.61
	**2 weeks**	**5**	30.79	30.51
	**4 weeks**	**5**	31.50	31.34
	**12 weeks**	**6**	33.82	34.05
**mmu-miR-370-001068**	**2 days**	**4**	29.84	29.53
	**2 weeks**	**5**	.	.

	**4 weeks**	**5**	.	.
	**12 weeks**	**6**	.	.
**mmu-miR-370-4395386**	**2 days**	**4**	25.07	25.08
	**2 weeks**	**5**	29.84	29.67
	**4 weeks**	**5**	32.70	32.44
	**12 weeks**	**6**	32.58	32.99
**mmu-miR-374-5p-001319**	**2 days**	**4**	26.10	26.09
	**2 weeks**	**5**	27.12	26.79
	**4 weeks**	**5**	26.58	26.60
	**12 weeks**	**6**	26.50	26.48
**mmu-miR-376a#-002482**	**2 days**	**4**	27.80	27.73
	**2 weeks**	**5**	29.54	29.06
	**4 weeks**	**5**	30.40	30.43
	**12 weeks**	**6**	34.20	33.78
**mmu-miR-376a-4373347**	**2 days**	**4**	26.83	26.00
	**2 weeks**	**5**	29.83	29.13
	**4 weeks**	**5**	31.25	31.20
	**12 weeks**	**6**	33.06	32.97
**mmu-miR-376b#-002451**	**2 days**	**4**	25.38	25.38
	**2 weeks**	**5**	26.65	25.97
	**4 weeks**	**5**	27.44	27.59
	**12 weeks**	**6**	30.20	30.16
**mmu-miR-376b-4395582**	**2 days**	**4**	27.32	26.65
	**2 weeks**	**5**	31.25	30.52
	**4 weeks**	**5**	32.05	31.70
	**12 weeks**	**6**	34.38	34.40
**mmu-miR-376c-4395580**	**2 days**	**4**	27.39	24.61
	**2 weeks**	**5**	26.23	25.75
	**4 weeks**	**5**	26.94	27.02
	**12 weeks**	**6**	28.81	28.90
**mmu-miR-379-4373349**	**2 days**	**4**	24.93	24.30
	**2 weeks**	**5**	26.10	25.74
	**4 weeks**	**5**	27.01	26.98
	**12 weeks**	**6**	29.59	29.53
**mmu-miR-380-5p-4395731**	**2 days**	**4**	25.29	25.35
	**2 weeks**	**5**	28.02	27.46
	**4 weeks**	**5**	29.67	29.72
	**12 weeks**	**6**	32.69	32.59
**mmu-miR-381-4373020**	**2 days**	**4**	28.27	29.52
	**2 weeks**	**5**	32.24	32.36
	**4 weeks**	**5**	33.56	34.41
	**12 weeks**	**6**	34.52	34.57
**mmu-miR-382-4373019**	**2 days**	**4**	25.43	25.03
	**2 weeks**	**5**	26.03	25.85
	**4 weeks**	**5**	27.26	27.40
	**12 weeks**	**6**	29.65	29.33
**mmu-miR-409-3p-4395443**	**2 days**	**4**	23.62	23.75
	**2 weeks**	**5**	28.82	28.17

	**4 weeks**	**5**	29.19	29.17
	**12 weeks**	**6**	31.43	31.41
**mmu-miR-409-5p-4395442**	**2 days**	**4**	28.15	28.12
	**2 weeks**	**5**	29.58	29.46
	**4 weeks**	**5**	30.61	30.48
	**12 weeks**	**6**	32.98	33.18
**mmu-miR-410-4378093**	**2 days**	**4**	24.82	24.46
	**2 weeks**	**5**	27.74	27.07
	**4 weeks**	**5**	29.39	29.35
	**12 weeks**	**6**	32.59	32.63
**mmu-miR-411-4381013**	**2 days**	**4**	25.58	22.46
	**2 weeks**	**5**	24.47	24.10
	**4 weeks**	**5**	25.70	25.73
	**12 weeks**	**6**	27.97	28.01
**mmu-miR-412-002575**	**2 days**	**4**	27.96	27.97
	**2 weeks**	**5**	31.29	31.42
	**4 weeks**	**5**	33.64	33.87
	**12 weeks**	**6**	33.06	33.06
**mmu-miR-425-4380926**	**2 days**	**4**	28.47	29.19
	**2 weeks**	**5**	30.79	30.89
	**4 weeks**	**5**	30.63	29.63
	**12 weeks**	**6**	29.57	29.68
**mmu-miR-431-4395173**	**2 days**	**4**	20.45	20.61
	**2 weeks**	**5**	24.05	23.60
	**4 weeks**	**5**	25.85	25.65
	**12 weeks**	**6**	30.41	30.27
**mmu-miR-433-4373205**	**2 days**	**4**	25.07	24.76
	**2 weeks**	**5**	27.95	28.69
	**4 weeks**	**5**	31.74	30.86
	**12 weeks**	**6**	34.40	34.45
**mmu-miR-434-3p-4395734**	**2 days**	**4**	23.38	22.16
	**2 weeks**	**5**	23.83	23.27
	**4 weeks**	**5**	24.99	25.07
	**12 weeks**	**6**	27.28	27.27
**mmu-miR-434-5p-4395711**	**2 days**	**4**	25.85	26.40
	**2 weeks**	**5**	27.62	27.56
	**4 weeks**	**5**	29.71	29.66
	**12 weeks**	**6**	31.78	31.81
**mmu-miR-448-4373206**	**2 days**	**4**	28.89	28.89
	**2 weeks**	**5**	.	.
	**4 weeks**	**5**	.	.
	**12 weeks**	**6**	23.42	23.42
**mmu-miR-450B-3P-002632**	**2 days**	**4**	29.84	29.97
	**2 weeks**	**5**	31.31	30.85
	**4 weeks**	**5**	32.42	32.40
	**12 weeks**	**6**	34.84	34.38
**mmu-miR-450a-5p-4395414**	**2 days**	**4**	29.48	29.96
	**2 weeks**	**5**	31.65	31.71

	4 weeks	5	33.28	33.25
	12 weeks	6	34.48	34.89
mmu-miR-451-4373360	2 days	4	30.61	30.63
	2 weeks	5	31.59	31.25
	4 weeks	5	30.74	30.76
	12 weeks	6	30.73	30.65
mmu-miR-455-4395585	2 days	4	29.21	28.59
	2 weeks	5	33.42	33.11
	4 weeks	5	32.34	32.33
	12 weeks	6	36.10	36.29
mmu-miR-464-4373362	2 days	4	28.58	28.58
	2 weeks	5	.	.
	4 weeks	5	.	.
	12 weeks	6	.	.
mmu-miR-466h-4395646	2 days	4	27.83	27.83
	2 weeks	5	.	.
	4 weeks	5	.	.
	12 weeks	6	.	.
mmu-miR-467F-002886	2 days	4	26.02	26.06
	2 weeks	5	27.01	26.80
	4 weeks	5	27.01	26.90
	12 weeks	6	27.04	27.02
mmu-miR-467a-001826	2 days	4	29.71	29.69
	2 weeks	5	32.55	31.81
	4 weeks	5	33.87	32.68
	12 weeks	6	33.89	33.57
mmu-miR-467a-4395717	2 days	4	29.35	29.36
	2 weeks	5	31.92	31.94
	4 weeks	5	32.36	31.34
	12 weeks	6	33.73	33.72
mmu-miR-470-4395718	2 days	4	29.39	29.39
	2 weeks	5	.	.
	4 weeks	5	.	.
	12 weeks	6	.	.
mmu-miR-471-002605	2 days	4	.	.
	2 weeks	5	.	.
	4 weeks	5	.	.
	12 weeks	6	14.99	14.99
mmu-miR-483#-002560	2 days	4	23.15	23.08
	2 weeks	5	26.43	26.00
	4 weeks	5	28.70	28.38
	12 weeks	6	.	.
mmu-miR-483-001291	2 days	4	25.59	25.60
	2 weeks	5	27.68	27.19
	4 weeks	5	29.19	28.98
	12 weeks	6	.	.
mmu-miR-484-4381032	2 days	4	23.87	23.80
	2 weeks	5	25.68	25.18

	4 weeks	5	25.70	25.63
	12 weeks	6	25.24	25.24
mmu-miR-485-3p-001943	2 days	4	23.42	23.45
	2 weeks	5	29.20	28.97
	4 weeks	5	29.78	29.57
	12 weeks	6	31.53	31.44
mmu-miR-486-4378096	2 days	4	24.90	24.79
	2 weeks	5	25.32	24.73
	4 weeks	5	24.33	24.27
	12 weeks	6	23.90	23.94
mmu-miR-487b-001306	2 days	4	25.16	25.21
	2 weeks	5	28.40	27.77
	4 weeks	5	30.02	29.95
	12 weeks	6	33.64	33.37
mmu-miR-487b-4378102	2 days	4	24.99	24.97
	2 weeks	5	28.79	28.23
	4 weeks	5	30.27	30.31
	12 weeks	6	34.00	33.39
mmu-miR-488#-002014	2 days	4	.	.
	2 weeks	5	.	.
	4 weeks	5	37.10	37.10
	12 weeks	6	24.85	24.85
mmu-miR-488-4381074	2 days	4	28.08	28.08
	2 weeks	5	39.95	39.95
	4 weeks	5	.	.
	12 weeks	6	.	.
mmu-miR-489-4378114	2 days	4	28.70	30.59
	2 weeks	5	30.61	30.70
	4 weeks	5	31.93	31.73
	12 weeks	6	32.70	32.56
mmu-miR-490-4373215	2 days	4	25.24	25.24
	2 weeks	5	.	.
	4 weeks	5	31.22	31.22
	12 weeks	6	38.32	38.32
mmu-miR-491-4381053	2 days	4	30.95	30.97
	2 weeks	5	32.16	32.21
	4 weeks	5	31.71	31.40
	12 weeks	6	30.13	30.24
mmu-miR-493-4395649	2 days	4	26.57	27.59
	2 weeks	5	31.39	30.69
	4 weeks	5	33.41	32.87
	12 weeks	6	31.98	31.98
mmu-miR-494-4395476	2 days	4	27.77	27.68
	2 weeks	5	28.85	28.51
	4 weeks	5	28.86	28.85
	12 weeks	6	29.87	29.85
mmu-miR-495-4381078	2 days	4	23.84	22.41
	2 weeks	5	27.17	26.69

	4 weeks	5	27.92	27.96
	12 weeks	6	29.87	29.99
mmu-miR-496-4386771	2 days	4	27.90	29.44
	2 weeks	5	29.29	31.36
	4 weeks	5	32.04	33.59
	12 weeks	6	34.16	34.23
mmu-miR-497-4381046	2 days	4	30.38	30.32
	2 weeks	5	29.82	29.39
	4 weeks	5	28.95	28.84
	12 weeks	6	29.07	29.20
mmu-miR-500-4395736	2 days	4	30.29	29.72
	2 weeks	5	31.58	31.46
	4 weeks	5	31.28	31.32
	12 weeks	6	32.87	32.52
mmu-miR-501-3p-4381069	2 days	4	28.06	27.45
	2 weeks	5	28.85	29.14
	4 weeks	5	29.84	29.77
	12 weeks	6	31.90	31.16
mmu-miR-503#-002536	2 days	4	27.10	26.96
	2 weeks	5	30.44	29.98
	4 weeks	5	30.90	30.78
	12 weeks	6	32.36	32.35
mmu-miR-503-4395586	2 days	4	27.94	26.26
	2 weeks	5	29.53	28.84
	4 weeks	5	29.56	29.80
	12 weeks	6	31.13	31.10
mmu-miR-505-4381071	2 days	4	30.18	30.18
	2 weeks	5	.	.
	4 weeks	5	.	.
	12 weeks	6	.	.
mmu-miR-532-3p-4395466	2 days	4	27.74	26.12
	2 weeks	5	28.09	27.37
	4 weeks	5	28.40	28.36
	12 weeks	6	29.12	29.26
mmu-miR-532-5p-4380928	2 days	4	26.29	24.28
	2 weeks	5	26.49	25.81
	4 weeks	5	26.44	26.42
	12 weeks	6	27.53	27.44
mmu-miR-539-4378103	2 days	4	25.57	22.93
	2 weeks	5	24.83	24.31
	4 weeks	5	25.35	25.40
	12 weeks	6	27.48	27.47
mmu-miR-540-3p-4378119	2 days	4	25.55	27.52
	2 weeks	5	29.11	28.49
	4 weeks	5	30.75	30.48
	12 weeks	6	34.43	34.43
mmu-miR-541-002562	2 days	4	27.35	27.25
	2 weeks	5	30.31	29.97

	4 weeks	5	31.49	31.58
	12 weeks	6	35.01	34.15
mmu-miR-542-3p-4378101	2 days	4	30.73	31.67
	2 weeks	5	33.29	33.31
	4 weeks	5	35.95	34.79
	12 weeks	6	.	.
mmu-miR-542-5p-4395693	2 days	4	28.36	28.36
	2 weeks	5	30.93	29.87
	4 weeks	5	31.39	31.24
	12 weeks	6	33.90	34.13
mmu-miR-543-001298	2 days	4	23.94	24.05
	2 weeks	5	30.62	29.98
	4 weeks	5	32.42	30.98
	12 weeks	6	37.42	38.40
mmu-miR-543-4395487	2 days	4	26.06	24.45
	2 weeks	5	30.58	30.58
	4 weeks	5	32.58	32.57
	12 weeks	6	33.23	33.32
mmu-miR-544-4395680	2 days	4	29.74	28.55
	2 weeks	5	30.30	29.84
	4 weeks	5	30.93	30.68
	12 weeks	6	33.70	33.26
mmu-miR-546-4381044	2 days	4	30.21	31.02
	2 weeks	5	32.60	32.69
	4 weeks	5	31.14	31.53
	12 weeks	6	32.86	32.71
mmu-miR-574-3p-4395460	2 days	4	25.32	25.52
	2 weeks	5	28.95	28.78
	4 weeks	5	29.76	29.69
	12 weeks	6	28.12	28.01
mmu-miR-615-3p-4386777	2 days	4	30.16	30.42
	2 weeks	5	33.36	32.60
	4 weeks	5	32.56	32.56
	12 weeks	6	34.68	35.65
mmu-miR-652-4395463	2 days	4	25.70	27.39
	2 weeks	5	30.11	29.22
	4 weeks	5	30.31	30.22
	12 weeks	6	30.40	30.39
mmu-miR-654-3p-4395350	2 days	4	28.37	28.37
	2 weeks	5	.	.
	4 weeks	5	.	.
	12 weeks	6	.	.
mmu-miR-665-4395737	2 days	4	28.12	27.92
	2 weeks	5	31.04	31.01
	4 weeks	5	34.48	35.39
	12 weeks	6	35.11	35.11
mmu-miR-666-5p-4386770	2 days	4	27.87	28.59
	2 weeks	5	33.67	32.81

	4 weeks	5	35.74	35.16
	12 weeks	6	35.83	35.83
mmu-miR-667-4386769	2 days	4	22.17	23.57
	2 weeks	5	28.79	28.36
	4 weeks	5	30.11	30.16
	12 weeks	6	31.17	31.34
mmu-miR-668-4386767	2 days	4	27.75	27.92
	2 weeks	5	32.18	32.25
	4 weeks	5	29.06	29.06
	12 weeks	6	.	.
mmu-miR-670-4395561	2 days	4	28.45	28.45
	2 weeks	5	.	.
	4 weeks	5	30.73	30.73
	12 weeks	6	.	.
mmu-miR-672-4395438	2 days	4	29.34	28.53
	2 weeks	5	31.92	31.67
	4 weeks	5	32.07	32.03
	12 weeks	6	33.45	33.19
mmu-miR-673-001954	2 days	4	25.42	25.38
	2 weeks	5	31.72	31.79
	4 weeks	5	32.55	32.34
	12 weeks	6	32.93	32.92
mmu-miR-673-3p-002449	2 days	4	25.49	25.24
	2 weeks	5	27.51	27.14
	4 weeks	5	28.91	28.85
	12 weeks	6	31.96	31.83
mmu-miR-674#-001956	2 days	4	29.29	29.22
	2 weeks	5	30.82	30.34
	4 weeks	5	30.81	31.00
	12 weeks	6	31.05	30.85
mmu-miR-674-4395193	2 days	4	29.49	29.66
	2 weeks	5	31.26	31.19
	4 weeks	5	31.69	32.00
	12 weeks	6	31.42	30.70
mmu-miR-675-3p-4386762	2 days	4	28.53	29.63
	2 weeks	5	30.99	30.83
	4 weeks	5	30.44	30.82
	12 weeks	6	32.64	32.60
mmu-miR-675-5p-4386761	2 days	4	25.24	25.24
	2 weeks	5	.	.
	4 weeks	5	.	.
	12 weeks	6	.	.
mmu-miR-676-4386776	2 days	4	27.50	27.14
	2 weeks	5	24.34	27.62
	4 weeks	5	28.35	28.36
	12 weeks	6	28.74	28.77
mmu-miR-677-4381075	2 days	4	22.69	22.69
	2 weeks	5	39.69	39.69

	4 weeks	5	37.24	37.24
	12 weeks	6	35.08	35.08
mmu-miR-679-4381077	2 days	4	33.53	34.74
	2 weeks	5	33.72	33.72
	4 weeks	5	26.11	26.11
	12 weeks	6	.	.
mmu-miR-684-4381083	2 days	4	24.02	24.02
	2 weeks	5	35.61	35.61
	4 weeks	5	.	.
	12 weeks	6	38.97	38.97
mmu-miR-685-4386748	2 days	4	28.22	30.80
	2 weeks	5	31.84	31.84
	4 weeks	5	31.25	30.86
	12 weeks	6	31.39	31.17
mmu-miR-686-4381085	2 days	4	26.96	26.96
	2 weeks	5	.	.
	4 weeks	5	.	.
	12 weeks	6	.	.
mmu-miR-687-4386750	2 days	4	28.89	29.16
	2 weeks	5	30.31	29.83
	4 weeks	5	29.49	29.64
	12 weeks	6	29.58	29.45
mmu-miR-690-001677	2 days	4	26.58	26.38
	2 weeks	5	28.29	27.42
	4 weeks	5	26.74	27.24
	12 weeks	6	28.96	28.84
mmu-miR-696-001628	2 days	4	29.41	28.89
	2 weeks	5	30.13	30.60
	4 weeks	5	30.39	30.35
	12 weeks	6	28.87	29.25
mmu-miR-706-001641	2 days	4	28.02	28.02
	2 weeks	5	30.45	29.53
	4 weeks	5	29.79	29.89
	12 weeks	6	30.32	29.92
mmu-miR-708-4395452	2 days	4	30.41	30.05
	2 weeks	5	31.08	30.98
	4 weeks	5	31.04	31.10
	12 weeks	6	31.69	31.79
mmu-miR-720-001629	2 days	4	21.85	21.49
	2 weeks	5	24.49	23.84
	4 weeks	5	24.10	24.23
	12 weeks	6	23.50	23.46
mmu-miR-742-4395573	2 days	4	25.24	25.24
	2 weeks	5	.	.
	4 weeks	5	.	.
	12 weeks	6	.	.
mmu-miR-743b-5p-4395600	2 days	4	29.76	29.76
	2 weeks	5	.	.

	**4 weeks**	**5**	.	.
	**12 weeks**	**6**	.	.
**mmu-miR-744-4395435**	**2 days**	**4**	27.72	28.40
	**2 weeks**	**5**	31.38	31.48
	**4 weeks**	**5**	31.74	31.66
	**12 weeks**	**6**	30.80	30.84
**mmu-miR-770-3p-4395564**	**2 days**	**4**	29.81	29.56
	**2 weeks**	**5**	8.92	8.92
	**4 weeks**	**5**	21.05	21.05
	**12 weeks**	**6**	28.36	28.36
**mmu-miR-7a-4378130**	**2 days**	**4**	25.39	25.39
	**2 weeks**	**5**	34.28	34.28
	**4 weeks**	**5**	35.29	34.81
	**12 weeks**	**6**	35.24	35.72
**mmu-miR-802-4395566**	**2 days**	**4**	26.14	26.14
	**2 weeks**	**5**	.	.
	**4 weeks**	**5**	.	.
	**12 weeks**	**6**	.	.
**mmu-miR-805-002045**	**2 days**	**4**	25.87	25.87
	**2 weeks**	**5**	26.65	25.95
	**4 weeks**	**5**	25.22	25.32
	**12 weeks**	**6**	24.82	24.80
**mmu-miR-872#-002542**	**2 days**	**4**	27.15	27.01
	**2 weeks**	**5**	30.30	29.10
	**4 weeks**	**5**	29.28	29.41
	**12 weeks**	**6**	28.65	28.57
**mmu-miR-872-4395375**	**2 days**	**4**	28.21	29.18
	**2 weeks**	**5**	30.76	30.60
	**4 weeks**	**5**	30.65	30.80
	**12 weeks**	**6**	30.21	30.04
**mmu-miR-873-4395467**	**2 days**	**4**	20.05	20.05
	**2 weeks**	**5**	32.76	32.76
	**4 weeks**	**5**	.	.
	**12 weeks**	**6**	.	.
**mmu-miR-874-4395379**	**2 days**	**4**	20.17	20.17
	**2 weeks**	**5**	.	.
	**4 weeks**	**5**	.	.
	**12 weeks**	**6**	.	.
**mmu-miR-875-3p-4395677**	**2 days**	**4**	30.33	30.33
	**2 weeks**	**5**	.	.
	**4 weeks**	**5**	.	.
	**12 weeks**	**6**	.	.
**mmu-miR-876-3p-4395594**	**2 days**	**4**	30.10	30.10
	**2 weeks**	**5**	.	.
	**4 weeks**	**5**	.	.
	**12 weeks**	**6**	.	.
**mmu-miR-876-5p-4395593**	**2 days**	**4**	28.31	28.31
	**2 weeks**	**5**	.	.

	4 weeks	5	.	.
	12 weeks	6	.	.
mmu-miR-877#-002548	2 days	4	28.45	28.52
	2 weeks	5	30.41	30.33
	4 weeks	5	31.14	31.16
	12 weeks	6	30.19	30.19
mmu-miR-878-5p-4395670	2 days	4	29.32	29.32
	2 weeks	5	.	.
	4 weeks	5	.	.
	12 weeks	6	.	.
mmu-miR-879-4395602	2 days	4	22.24	22.24
	2 weeks	5	.	.
	4 weeks	5	.	.
	12 weeks	6	.	.
mmu-miR-881-4395739	2 days	4	27.80	27.80
	2 weeks	5	.	.
	4 weeks	5	.	.
	12 weeks	6	.	.
mmu-miR-883a-3p-4395591	2 days	4	25.46	25.46
	2 weeks	5	.	.
	4 weeks	5	.	.
	12 weeks	6	.	.
mmu-miR-883b-3p-4395695	2 days	4	27.38	27.38
	2 weeks	5	.	.
	4 weeks	5	.	.
	12 weeks	6	.	.
mmu-miR-92a-4373013	2 days	4	26.88	26.85
	2 weeks	5	28.51	28.28
	4 weeks	5	28.44	28.48
	12 weeks	6	28.07	28.13
mmu-miR-93-4373302	2 days	4	28.06	28.09
	2 weeks	5	29.46	28.77
	4 weeks	5	28.67	28.72
	12 weeks	6	28.23	28.31
mmu-miR-96-4373372	2 days	4	.	.
	2 weeks	5	.	.
	4 weeks	5	39.13	39.13
	12 weeks	6	24.41	24.41
mmu-miR-99a-4373008	2 days	4	32.60	32.77
	2 weeks	5	31.00	30.83
	4 weeks	5	30.63	30.97
	12 weeks	6	30.94	30.49
mmu-miR-99b-4373007	2 days	4	26.97	26.92
	2 weeks	5	28.90	28.53
	4 weeks	5	28.24	28.05
	12 weeks	6	28.41	28.40
rno-miR-1-4395765	2 days	4	25.22	22.32
	2 weeks	5	21.91	21.67

	4 weeks	5	20.97	20.85
	12 weeks	6	20.21	20.23
rno-miR-146B-002755	2 days	4	28.59	28.52
	2 weeks	5	29.94	29.12
	4 weeks	5	28.79	28.66
	12 weeks	6	27.32	27.33
rno-miR-17-3p-4395779	2 days	4	31.13	31.13
	2 weeks	5	31.53	31.53
	4 weeks	5	.	.
	12 weeks	6	.	.
rno-miR-196c-4395750	2 days	4	30.12	30.00
	2 weeks	5	31.93	31.39
	4 weeks	5	31.35	31.36
	12 weeks	6	30.74	30.76
rno-miR-20b-001326	2 days	4	30.70	30.61
	2 weeks	5	31.86	30.97
	4 weeks	5	31.26	31.25
	12 weeks	6	31.36	31.50
rno-miR-29c#-001818	2 days	4	34.97	34.97
	2 weeks	5	33.96	33.83
	4 weeks	5	32.66	32.57
	12 weeks	6	30.62	30.32
rno-miR-327-4381108	2 days	4	27.68	27.68
	2 weeks	5	37.16	37.16
	4 weeks	5	37.24	37.24
	12 weeks	6	36.48	36.48
rno-miR-336-4381111	2 days	4	25.81	25.81
	2 weeks	5	.	.
	4 weeks	5	.	.
	12 weeks	6	.	.
rno-miR-339-3p-4395760	2 days	4	29.29	31.18
	2 weeks	5	35.18	35.39
	4 weeks	5	34.16	33.56
	12 weeks	6	33.93	34.25
rno-miR-345-3p-4395762	2 days	4	29.91	30.56
	2 weeks	5	32.59	32.01
	4 weeks	5	32.06	32.04
	12 weeks	6	31.74	31.32
rno-miR-350-001337	2 days	4	30.31	30.10
	2 weeks	5	32.11	32.18
	4 weeks	5	30.96	30.66
	12 weeks	6	30.13	29.86
rno-miR-351-4395764	2 days	4	26.37	26.14
	2 weeks	5	30.16	29.62
	4 weeks	5	30.60	30.74
	12 weeks	6	33.33	33.08
rno-miR-352-001339	2 days	4	29.51	29.42
	2 weeks	5	30.12	29.52

	4 weeks	5	29.74	29.59
	12 weeks	6	29.89	30.09
rno-miR-379#-002081	2 days	4	28.08	28.15
	2 weeks	5	31.18	30.82
	4 weeks	5	33.26	32.75
	12 weeks	6	35.78	35.29
rno-miR-381-4381102	2 days	4	27.48	27.47
	2 weeks	5	29.23	29.03
	4 weeks	5	30.59	30.91
	12 weeks	6	33.18	32.89
rno-miR-382#-001354	2 days	4	29.35	29.23
	2 weeks	5	31.43	31.25
	4 weeks	5	31.69	31.87
	12 weeks	6	34.97	34.87
rno-miR-409-3P-002679	2 days	4	23.36	23.25
	2 weeks	5	28.48	27.94
	4 weeks	5	29.20	29.29
	12 weeks	6	31.59	31.48
rno-miR-466c-4395768	2 days	4	30.34	30.34
	2 weeks	5	.	.
	4 weeks	5	.	.
	12 weeks	6	.	.
rno-miR-489-001353	2 days	4	31.07	30.78
	2 weeks	5	31.09	30.46
	4 weeks	5	31.93	32.08
	12 weeks	6	33.12	32.97
rno-miR-504-007334	2 days	4	.	.
	2 weeks	5	37.83	37.83
	4 weeks	5	14.04	14.04
	12 weeks	6	.	.
rno-miR-513-241072_mat	2 days	4	19.35	19.35
	2 weeks	5	.	.
	4 weeks	5	.	.
	12 weeks	6	.	.
rno-miR-532-5p-4395752	2 days	4	27.24	27.35
	2 weeks	5	29.17	28.38
	4 weeks	5	28.99	28.93
	12 weeks	6	30.62	30.73
rno-miR-632-241110_mat	2 days	4	29.98	29.82
	2 weeks	5	30.24	29.27
	4 weeks	5	29.64	29.90
	12 weeks	6	29.72	29.75
rno-miR-653-241125_mat	2 days	4	10.83	10.83
	2 weeks	5	14.37	14.37
	4 weeks	5	.	.
	12 weeks	6	.	.
rno-miR-664-001323	2 days	4	30.65	30.72
	2 weeks	5	31.14	30.54

	4 weeks	5	29.67	29.74
	12 weeks	6	27.84	27.91
rno-miR-673-4395755	2 days	4	29.29	30.19
	2 weeks	5	36.47	36.73
	4 weeks	5	37.68	38.14
	12 weeks	6	37.09	37.49
rno-miR-7#-001338	2 days	4	30.10	29.98
	2 weeks	5	30.76	30.02
	4 weeks	5	29.25	29.28
	12 weeks	6	27.51	27.52
rno-miR-743b-4395769	2 days	4	30.58	30.58
	2 weeks	5	37.58	37.64
	4 weeks	5	37.89	39.07
	12 weeks	6	37.27	37.68
rno-miR-758-4395180	2 days	4	29.53	29.29
	2 weeks	5	32.62	32.60
	4 weeks	5	32.25	32.25
	12 weeks	6	33.44	33.44
rno-miR-760-5p-4395758	2 days	4	30.43	30.43
	2 weeks	5	.	.
	4 weeks	5	.	.
	12 weeks	6	37.86	37.86
rno-miR-7a#-002062	2 days	4	28.31	28.20
	2 weeks	5	29.03	28.53
	4 weeks	5	27.61	27.64
	12 weeks	6	26.34	26.37
rno-miR-881-4395773	2 days	4	23.54	23.54
	2 weeks	5	.	.
	4 weeks	5	.	.
	12 weeks	6	.	.
snoRNA135-001230	2 days	4	25.76	25.67
	2 weeks	5	27.18	26.55
	4 weeks	5	26.88	26.85
	12 weeks	6	27.35	27.29
snoRNA135-4380912	2 days	4	25.36	25.33
	2 weeks	5	26.81	26.29
	4 weeks	5	26.80	26.60
	12 weeks	6	26.83	26.91
snoRNA202-001232	2 days	4	22.44	22.27
	2 weeks	5	23.81	23.21
	4 weeks	5	23.50	23.15
	12 weeks	6	23.85	23.77
snoRNA202-4380914	2 days	4	24.63	22.60
	2 weeks	5	23.81	23.30
	4 weeks	5	23.49	23.22
	12 weeks	6	23.70	23.66
