# Supplementary material for: MicroRNA expression patterns in post-natal mouse skeletal muscle development
Source: BMC Genomics. 2017 Jan 7;18:52. doi: 10.1186/s12864-016-3399-2 (PMC5219731; doi:10.1186/s12864-016-3399-2)
Supplement: Additional file 2: — List of the 205 miRNAs presenting a significant linear trend in their Ct values over time. 150 miRNAs had their Ct values increasing over time, while 55 had their Ct values decreasing over time. (PDF 102 kb) [file 12864_2016_3399_MOESM2_ESM.pdf]

Robust regression - time trends

rna	cp_slope	ProbChiSq
mmu-miR-29a-4395223	-0.0482	<.0001
rno-miR-29c#-001818	-0.0447	<.0001
mmu-miR-29c-4395171	-0.0403	<.0001
rno-miR-664-001323	-0.0359	<.0001
rno-miR-7#-001338	-0.0315	<.0001
hsa-miR-22-000398	-0.0273	<.0001
mmu-miR-195-4373105	-0.0273	<.0001
mmu-miR-30c-4373060	-0.0271	<.0001
rno-miR-7a#-002062	-0.0258	<.0001
hsa-miR-223-000526	-0.0252	<.0001
mmu-miR-365-4373194	-0.025	<.0001
mmu-miR-155-4395701	-0.0249	<.0001
mmu-miR-21-4373090	-0.0243	<.0001
mmu-miR-150-4373127	-0.0232	<.0001
rno-miR-146B-002755	-0.0223	<.0001
mmu-miR-222-4395387	-0.0216	<.0001
rno-miR-1-4395765	-0.021	<.0001
mmu-miR-223-4395406	-0.0206	<.0001
hsa-miR-30a-3p-00041	-0.0199	<.0001
mmu-miR-101a-4395364	-0.0194	<.0001
hsa-miR-30e-3p-00042	-0.0193	<.0001
mmu-miR-30e-4395334	-0.0192	<.0001
mmu-miR-193b-4395597	-0.0191	<.0001
hsa-miR-22#-002301	-0.0185	<.0001
mmu-miR-186-4395396	-0.0185	<.0001
mmu-miR-146a-4373132	-0.0183	<.0001
mmu-miR-133a-4395357	-0.0182	<.0001
mmu-miR-145-4395389	-0.0169	<.0001
mmu-miR-1-4395333	-0.0153	<.0001
mmu-miR-101b-002531	-0.0153	<.0001
mmu-miR-143-4395360	-0.0143	<.0001
mmu-miR-30b-4373290	-0.0143	<.0001
mmu-miR-193#-002577	-0.0135	<.0001
mmu-miR-805-002045	-0.0135	<.0001
mmu-miR-30a-4373061	-0.0132	<.0001
mmu-miR-142-3p-43731	-0.0131	<.0001
mmu-miR-26a-4395166	-0.013	<.0001
hsa-miR-213-000516	-0.0122	<.0001
mmu-miR-30d-4373059	-0.0111	<.0001
mmu-miR-486-4378096	-0.0103	<.0001
mmu-miR-15b-4373122	0.0077	<.0001
mmu-miR-214-4395417	0.0105	<.0001
snoRNA202-4380914	0.0115	<.0001
snoRNA135-4380912	0.013	<.0001

snoRNA202-001232	0.0144	<.0001
mmu-miR-2135-241140_	0.0155	<.0001
snoRNA135-001230	0.0157	<.0001
mmu-miR-676-4386776	0.0162	<.0001
mmu-miR-301a-4373064	0.0196	<.0001
mmu-miR-489-4378114	0.0211	<.0001
mmu-miR-494-4395476	0.023	<.0001
mmu-miR-362-3p-43957	0.0236	<.0001
mmu-miR-188-5p-43954	0.0244	<.0001
mmu-miR-362-5p-00261	0.0256	<.0001
mmu-miR-34b-3p-43957	0.0265	<.0001
mmu-miR-130a-4373145	0.0273	<.0001
mmu-miR-690-001677	0.0279	<.0001
mmu-miR-532-3p-43954	0.0282	<.0001
rno-miR-489-001353	0.0283	<.0001
mmu-miR-532-5p-43809	0.0302	<.0001
mmu-miR-301b-4395730	0.0307	<.0001
mmu-miR-675-3p-43867	0.0328	<.0001
hsa-miR-455-001280	0.0337	<.0001
hsa-miR-214#-002293	0.0343	<.0001
mmu-miR-18a-4395533	0.0345	<.0001
hsa-miR-421-002700	0.0347	<.0001
rno-miR-532-5p-43957	0.037	<.0001
mmu-miR-485-3p-00194	0.038	<.0001
mmu-miR-495-4381078	0.0381	<.0001
mmu-miR-503-4395586	0.0384	<.0001
mmu-miR-337-3p-43956	0.0403	<.0001
mmu-miR-667-4386769	0.0418	<.0001
mmu-miR-322-001059	0.0423	<.0001
mmu-miR-337-5p-43956	0.0423	<.0001
mmu-miR-467a-4395717	0.0423	<.0001
hsa-miR-106b#-002380	0.0448	<.0001
mmu-miR-409-3p-43954	0.0454	<.0001
mmu-miR-134-4373299	0.0459	<.0001
mmu-miR-376c-4395580	0.0466	<.0001
mmu-miR-376a-4373347	0.0485	<.0001
rno-miR-382#-001354	0.0492	<.0001
mmu-miR-503#-002536	0.0493	<.0001
mmu-miR-136-4395641	0.0496	<.0001
mmu-miR-539-4378103	0.0496	<.0001
mmu-miR-337-000193	0.0498	<.0001
mmu-miR-335-3p-43952	0.0505	<.0001
mmu-miR-450a-5p-4395	0.0511	<.0001
mmu-miR-335-5p-43730	0.0515	<.0001
mmu-miR-382-4373019	0.0521	<.0001
mmu-miR-450B-3P-0026	0.0522	<.0001
hsa-miR-299-5p-00060	0.0525	<.0001
mmu-miR-296-5p-43730	0.0534	<.0001

hsa-miR-136#-002100	0.0535	<.0001
mmu-miR-329-4373336	0.0549	<.0001
hsa-miR-206-000510	0.0558	<.0001
mmu-miR-544-4395680	0.0559	<.0001
mmu-miR-369-5p-43731	0.0568	<.0001
mmu-miR-379-4373349	0.0571	<.0001
mmu-miR-434-5p-43957	0.0575	<.0001
mmu-miR-434-3p-43957	0.0583	<.0001
mmu-miR-376b#-002451	0.0586	<.0001
mmu-miR-299-002612	0.0587	<.0001
hsa-miR-411#-002238	0.0598	<.0001
mmu-miR-411-4381013	0.0603	<.0001
mmu-miR-409-5p-43954	0.0618	<.0001
hsa-miR-376a#-001287	0.0619	<.0001
mmu-miR-322#-002506	0.0623	<.0001
mmu-miR-1193-002794	0.0645	<.0001
mmu-miR-376b-4395582	0.0646	<.0001
mmu-miR-542-5p-43956	0.0655	<.0001
rno-miR-381-4381102	0.0661	<.0001
mmu-miR-323-3p-43953	0.0664	<.0001
rno-miR-351-4395764	0.0672	<.0001
mmu-miR-351-4373345	0.0675	<.0001
mmu-miR-455-4395585	0.0731	<.0001
mmu-miR-376a#-002482	0.0735	<.0001
mmu-miR-673-3p-00244	0.0738	<.0001
mmu-miR-127-4373147	0.0744	<.0001
rno-miR-409-3P-00267	0.0762	<.0001
mmu-miR-380-5p-43957	0.0786	<.0001
mmu-miR-541-002562	0.0818	<.0001
rno-miR-379#-002081	0.083	<.0001
mmu-miR-410-4378093	0.0841	<.0001
mmu-miR-487b-4378102	0.0904	<.0001
mmu-miR-540-3p-43781	0.0908	<.0001
mmu-miR-487b-001306	0.0917	<.0001
mmu-miR-433-4373205	0.099	<.0001
hsa-miR-493-3p-00128	0.1038	<.0001
mmu-miR-431-4395173	0.1059	<.0001
mmu-miR-483-001291	0.1362	<.0001
mmu-miR-543-001298	0.1483	<.0001
mmu-miR-542-3p-43781	0.1487	<.0001
mmu-miR-483#-002560	0.1972	<.0001
mmu-miR-668-4386767	0.3205	<.0001
mmu-miR-2182-241119_	0.0107	0.0001
mmu-miR-126-5p-43732	-0.0084	0.0002
hsa-miR-378-000567	-0.0056	0.0002
mmu-miR-674#-001956	0.016	0.0002
mmu-miR-652-4395463	0.0231	0.0003

mmu-miR-706-001641	0.0201	0.0004
hsa-miR-412-001023	0.0931	0.0004
mmu-miR-185-4395382	-0.0143	0.0005
mmu-miR-130b-4373144	0.0548	0.0005
hsa-miR-149-002255	-0.0079	0.0007
mmu-miR-133b-4395358	-0.0168	0.0008
mmu-miR-16-4373121	-0.0099	0.0008
mmu-miR-15a#-002488	0.0161	0.0008
mmu-miR-496-4386771	0.043	0.0008
hsa-miR-143-000466	-0.016	0.0009
mmu-miR-17-4395419	0.0049	0.0009
mmu-let-7a-4373169	0.0053	0.0009
mmu-miR-10b-4395329	-0.0157	0.001
mmu-miR-19b-4373098	0.0126	0.001
mmu-miR-106a-4395589	0.0133	0.0012
mmu-miR-100-4373160	-0.0077	0.0014
mmu-miR-125a-5p-4395	0.0122	0.0019
mmu-miR-26b-4395167	-0.008	0.0023
mmu-miR-543-4395487	0.0627	0.0029
mmu-miR-615-3p-43867	0.0502	0.0032
hsa-miR-214-000517	0.0264	0.0033
mmu-miR-1896-121128_	0.0131	0.0035
mmu-miR-221-4373077	-0.0209	0.0037
mmu-miR-138-4395395	0.0088	0.004
mmu-miR-370-4395386	0.0733	0.0041
mmu-miR-1839-3p-1212	0.0128	0.0042
mmu-miR-210-4373089	0.0268	0.0043
mmu-miR-199a-3p-4395	0.0147	0.0045
mmu-miR-381-4373020	0.045	0.0049
mmu-miR-1944-121189_	0.0145	0.0053
mmu-miR-298-4395728	0.0645	0.006
mmu-miR-342-3p-43953	0.0113	0.0061
mmu-miR-491-4381053	-0.019	0.0069
hsa-miR-15b#-002173	0.0375	0.0069
mmu-miR-500-4395736	0.027	0.0074
mmu-miR-2146-241082_	0.0066	0.0082
mmu-miR-467a-001826	0.0404	0.0082
mmu-miR-19a-4373099	0.0117	0.0085
mmu-miR-146b-4373178	-0.0181	0.0091
mmu-miR-672-4395438	0.0381	0.0092
mmu-miR-20a-4373286	0.0047	0.0097
mmu-miR-412-002575	0.0579	0.01
mmu-miR-300-000191	0.0539	0.0107
mmu-miR-666-5p-43867	0.0677	0.0107
mmu-miR-34c#-002584	0.0124	0.0134
mmu-miR-1971-121161_	0.0135	0.014
mmu-miR-708-4395452	0.0132	0.0143
mmu-miR-1939-121180_	0.0193	0.0151

mmu-miR-126-3p-43953	-0.0085	0.0156
hsa-miR-485-5p-00103	0.3061	0.017
mmu-miR-665-4395737	0.0876	0.0197
mmu-miR-322-4378107	0.0075	0.0211
mmu-miR-1905-121196_	0.0207	0.0212
hsa-miR-30d#-002305	0.0114	0.0229
mmu-miR-140-4373374	0.012	0.0231
mmu-miR-218-4373081	0.0106	0.0233
mmu-miR-673-001954	0.0144	0.0249
rno-miR-673-4395755	0.0521	0.0263
mmu-miR-99b-4373007	0.0103	0.0295
mmu-miR-501-3p-43810	0.0234	0.0301
mmu-miR-148a-4373130	0.0099	0.0303
mmu-miR-467F-002886	0.0078	0.0321
mmu-miR-31-4373331	0.0205	0.0353
mmu-let-7i-4395332	0.0047	0.037
hsa-miR-338-5P-00265	0.0088	0.0374
hsa-miR-324-3p-00057	0.012	0.041
hsa-miR-140-3p-00223	0.0143	0.043
mmu-miR-192-4373108	-0.0063	0.045
rno-miR-758-4395180	0.0443	0.0477
mmu-miR-152-4395170	0.0104	0.0498
mmu-miR-493-4395649	0.0728	0.0522
mmu-miR-28-4373067	0.0045	0.0528
mmu-miR-199a-5p-4373	0.0228	0.053
mmu-miR-25-4373071	0.0075	0.0548
hsa-miR-27a#-002445	0.0131	0.0559
mmu-miR-2138-241080_	0.0118	0.0562
mmu-let-7c-4373167	0.006	0.0681
mmu-miR-34a-4395168	-0.0082	0.088
mmu-miR-497-4381046	-0.0075	0.0901
mmu-miR-331-3p-43730	-0.0146	0.103
mmu-miR-196b-4395326	0.01	0.106
mmu-miR-340-3p-43953	-0.0072	0.1101
mmu-miR-679-4381077	-0.2661	0.128
mmu-miR-191-4395410	-0.0054	0.1296
hsa-miR-151-5P-00264	-0.0053	0.1384
mmu-miR-93-4373302	-0.0042	0.1483
hsa-let-7f-1#-002417	-0.0303	0.149
mmu-let-7f-4373164	-0.0059	0.1551
mmu-miR-339-5p-43953	-0.0082	0.1557
mmu-miR-27a-4373287	-0.0017	0.1572
mmu-miR-203-4373095	-0.0079	0.1609
mmu-miR-2134-241120_	0.0045	0.1764
mmu-miR-15a-4373123	-0.0136	0.2
mmu-miR-340-5p-43953	-0.0047	0.2062
mmu-miR-696-001628	-0.0105	0.2239
rno-miR-20b-001326	0.0074	0.2269

mmu-miR-324-3p-43956	0.0088	0.228
mmu-miR-24-4373072	0.0021	0.2335
mmu-miR-1961-197391_	0.0057	0.2362
mmu-miR-7a-4378130	0.0491	0.2367
mmu-let-7g-4395393	-0.0038	0.2382
hsa-miR-30c-1#-00210	0.026	0.2391
mmu-miR-877#-002548	0.0097	0.2428
mmu-miR-546-4381044	0.0163	0.2486
mmu-let-7e-4395517	0.01	0.2519
mmu-miR-297a#-002454	0.0159	0.2524
mmu-miR-1951-121165_	-0.0111	0.2572
mmu-miR-574-3p-43954	0.0133	0.2596
mmu-miR-1839-5p-1211	0.0051	0.2729
mmu-miR-224-4395683	0.0127	0.2739
mmu-miR-1981-121200_	0.0116	0.2797
rno-miR-352-001339	0.0046	0.2867
mmu-miR-674-4395193	0.0112	0.2932
mmu-miR-484-4381032	0.0048	0.3107
mmu-miR-425-4380926	-0.0076	0.3126
mmu-miR-194-4373106	-0.0073	0.3302
mmu-miR-99a-4373008	-0.0106	0.3363
mmu-miR-293-4386754	0.066	0.339
mmu-miR-216b-4395437	0.4049	0.3543
hsa-miR-423-3P-00262	0.0034	0.3578
rno-miR-339-3p-43957	0.015	0.36
mmu-miR-24-2#-002494	-0.002	0.3634
mmu-miR-374-5p-00131	0.0025	0.3714
mmu-miR-1937c-241011	0.0071	0.3735
rno-miR-350-001337	-0.0072	0.374
mmu-miR-92a-4373013	-0.0021	0.3828
mmu-miR-181a-4373117	0.0048	0.3884
mmu-miR-28#-002545	-0.0043	0.3991
mmu-miR-872-4395375	0.0043	0.4148
mmu-miR-200b-4395362	0.0117	0.4212
mmu-miR-132-4373143	-0.0029	0.4301
mmu-miR-872#-002542	0.0051	0.431
mmu-miR-330-4395341	0.5373	0.523
mmu-miR-204-4373094	0.0026	0.5366
mmu-miR-1937b-241023	0.0047	0.5536
mmu-miR-328-4373049	0.0049	0.5604
mmu-miR-200c-4395411	0.0152	0.5608
mmu-miR-744-4395435	0.0052	0.602
mmu-miR-677-4381075	0.0653	0.6667
rno-miR-345-3p-43957	0.0027	0.6897
mmu-miR-128a-4395327	0.0021	0.7086
rno-miR-743b-4395769	-0.0064	0.711
mmu-let-7b-4373168	0.0018	0.7304
mmu-miR-1198-002780	-0.0026	0.7367

mmu-miR-720-001629	0.0021	0.739
mmu-miR-23b-4373073	-0.0014	0.7548
mmu-miR-687-4386750	0.002	0.7557
mmu-let-7a#-002478	-0.0013	0.7737
rno-miR-632-241110_m	0.0015	0.78
mmu-miR-106b-4373155	-0.0005	0.7845
mmu-miR-139-5p-43954	-0.0009	0.785
mmu-miR-27b-4373068	-0.0006	0.7989
mmu-miR-1897-5p-1211	-0.0013	0.8075
mmu-miR-320-4395388	0.0015	0.8105
mmu-miR-103-4373158	-0.0011	0.8171
hsa-miR-93#-002139	0.0011	0.8297
mmu-miR-451-4373360	-0.0005	0.8753
mmu-miR-141#-002513	-0.0009	0.8879
mmu-miR-339-3p-43956	0.0011	0.8917
rno-miR-196c-4395750	-0.0005	0.9066
mmu-miR-212-002551	-0.0008	0.9088
rno-miR-327-4381108	0.0025	0.9297
mmu-miR-685-4386748	-0.0007	0.9346
mmu-miR-1274a-121150	0.0003	0.938
mmu-miR-151-3p-43733	-0.0007	0.9423
hsa-miR-196a-241070_	-0.0003	0.9553
mmu-miR-1904-121162_	0.0004	0.9614
mmu-let-7d-4395394	-0.0001	0.971
mmu-miR-770-3p-43955	0.0039	0.9755
mmu-miR-125b-5p-4373	0.0001	0.9791
mmu-miR-217-001133	-0.0017	0.9813
	.	.
