# Supplementary material for: MicroRNA expression patterns in post-natal mouse skeletal muscle development
Source: BMC Genomics. 2017 Jan 7;18:52. doi: 10.1186/s12864-016-3399-2 (PMC5219731; doi:10.1186/s12864-016-3399-2)
Supplement: Additional file 3: — Kruskal-Wallis’ tests were used to compare time Ct values for miRNAs with no significant trend. Data are presented as median and 25th and 75th percentile. (PDF 45 kb) [file 12864_2016_3399_MOESM3_ESM.pdf]

mrna	time	n	median	p25	p75	p
hsa-miR-151-5P-002642	2 days	4	30.24	29.60	30.79	0.015
	2 weeks	4	28.95	28.87	29.08	
	4 weeks	5	29.66	29.22	29.67	
	12 weeks	6	29.05	28.86	29.42	
hsa-miR-196a-241070_mat	2 days	4	24.02	23.93	24.35	0.003
	2 weeks	4	25.50	25.27	25.83	
	4 weeks	5	25.75	25.40	25.80	
	12 weeks	6	24.95	24.65	25.11	
hsa-miR-27a#-002445	2 days	4	30.80	30.40	31.54	0.029
	2 weeks	4	32.85	32.74	32.92	
	4 weeks	5	32.99	32.21	33.10	
	12 weeks	6	32.67	32.27	33.47	
hsa-miR-93#-002139	2 days	4	27.18	27.10	27.81	0.019
	2 weeks	4	28.37	27.92	28.67	
	4 weeks	5	28.96	28.60	29.11	
	12 weeks	6	28.61	28.38	28.65	
mmu-miR-128a-4395327	2 days	4	29.03	28.69	29.24	0.033
	2 weeks	4	29.99	29.96	30.05	
	4 weeks	5	30.41	30.08	30.84	
	12 weeks	6	29.85	29.54	30.04	
mmu-miR-130a-4373145	2 days	4	28.28	27.99	28.32	0.001
	2 weeks	4	28.92	28.61	29.07	
	4 weeks	5	30.08	29.97	30.31	
	12 weeks	6	30.81	30.69	30.95	
mmu-miR-151-3p-4373304	2 days	4	29.08	28.77	29.76	0.005
	2 weeks	4	30.34	30.11	30.86	
	4 weeks	5	31.96	31.77	32.48	
	12 weeks	6	29.80	29.65	30.45	
mmu-miR-1937b-241023_mat	2 days	4	18.46	17.98	19.39	0.003
	2 weeks	4	21.04	20.91	21.35	
	4 weeks	5	21.50	21.47	21.59	
	12 weeks	6	20.71	20.57	21.23	
mmu-miR-1937c-241011_mat	2 days	4	20.92	20.11	21.78	0.007
	2 weeks	4	23.39	22.99	23.68	
	4 weeks	5	23.89	23.62	23.91	
	12 weeks	6	23.05	22.83	23.66	
mmu-miR-196b-4395326	2 days	4	27.79	27.57	29.86	0.042
	2 weeks	4	29.00	29.00	29.20	
	4 weeks	5	30.02	29.76	30.11	
	12 weeks	6	29.56	29.26	29.85	
mmu-miR-1981-121200_mat	2 days	4	29.51	29.38	30.00	0.010
	2 weeks	4	32.20	31.62	32.93	
	4 weeks	5	33.44	32.42	34.29	
	12 weeks	6	32.01	31.49	32.40	
mmu-miR-199a-5p-4373272	2 days	4	30.19	25.34	31.01	0.034
	2 weeks	4	33.84	33.19	34.49	
	4 weeks	5	32.76	32.19	36.22	

	12 weeks	6	33.58	32.61	34.18	
mmu-miR-200c-4395411	2 days	4	29.54	27.07	30.87	0.048
	2 weeks	4	34.19	34.06	34.32	
	4 weeks	5	34.36	32.05	36.89	
	12 weeks	6	32.49	31.85	32.82	
mmu-miR-203-4373095	2 days	4	28.18	26.45	30.20	0.022
	2 weeks	4	31.62	30.96	31.89	
	4 weeks	5	30.44	30.22	30.87	
	12 weeks	6	30.30	29.96	30.55	
mmu-miR-2135-241140_mat	2 days	4	26.78	26.07	27.21	0.010
	2 weeks	4	26.28	26.04	26.31	
	4 weeks	5	26.08	25.66	26.12	
	12 weeks	6	27.32	27.10	27.53	
mmu-miR-21-4373090	2 days	4	27.96	26.81	31.88	0.015
	2 weeks	4	28.37	27.99	28.77	
	4 weeks	5	27.74	27.63	28.02	
	12 weeks	6	26.29	26.16	26.51	
mmu-miR-224-4395683	2 days	4	29.61	29.37	29.68	0.030
	2 weeks	4	32.01	31.50	32.30	
	4 weeks	5	32.98	32.75	34.49	
	12 weeks	6	31.76	31.54	32.88	
mmu-miR-25-4373071	2 days	4	29.87	28.73	29.93	0.014
	2 weeks	4	30.08	29.94	30.67	
	4 weeks	5	30.93	30.61	31.35	
	12 weeks	6	30.72	30.56	30.88	
mmu-miR-297a#-002454	2 days	4	30.55	29.95	30.92	0.013
	2 weeks	4	33.65	32.98	34.08	
	4 weeks	5	34.37	33.62	34.90	
	12 weeks	6	32.96	31.76	33.53	
mmu-miR-320-4395388	2 days	4	27.69	27.46	27.69	0.003
	2 weeks	4	28.75	28.55	28.97	
	4 weeks	5	29.83	29.59	29.85	
	12 weeks	6	28.74	28.52	28.84	
mmu-miR-324-3p-4395639	2 days	4	29.23	27.28	29.62	0.023
	2 weeks	4	30.92	30.43	31.49	
	4 weeks	5	31.76	31.20	32.19	
	12 weeks	6	31.10	30.96	31.21	
mmu-miR-328-4373049	2 days	4	26.11	25.26	26.46	0.004
	2 weeks	4	28.30	27.88	28.54	
	4 weeks	5	28.75	28.49	28.78	
	12 weeks	6	27.75	27.16	28.08	
mmu-miR-331-3p-4373046	2 days	4	27.31	27.09	29.45	0.016
	2 weeks	4	27.71	27.60	28.10	
	4 weeks	5	29.39	29.13	29.54	
	12 weeks	6	26.97	26.66	27.48	
mmu-miR-339-5p-4395368	2 days	4	30.70	28.05	31.18	0.027
	2 weeks	4	31.04	30.82	32.10	
	4 weeks	5	31.68	31.63	32.54	
	12 weeks	6	30.63	30.28	31.07	

mmu-miR-340-5p-4395369	2 days	4	29.86	28.25	30.12	0.002
	2 weeks	4	30.81	30.63	30.92	
	4 weeks	5	31.08	31.04	31.17	
	12 weeks	6	30.21	30.00	30.38	
mmu-miR-484-4381032	2 days	4	23.80	23.63	24.18	0.004
	2 weeks	4	25.17	25.07	25.25	
	4 weeks	5	25.63	25.55	25.85	
	12 weeks	6	25.24	25.05	25.50	
mmu-miR-493-4395649	2 days	4	27.59	25.38	27.75	0.012
	2 weeks	4	30.67	30.44	31.02	
	4 weeks	5	32.87	32.22	34.60	
	12 weeks	6	31.98	31.98	31.98	
mmu-miR-574-3p-4395460	2 days	4	25.52	24.86	25.79	0.002
	2 weeks	4	28.23	27.58	28.95	
	4 weeks	5	29.69	29.45	30.14	
	12 weeks	6	28.01	27.98	28.15	
mmu-miR-720-001629	2 days	4	21.49	21.21	22.48	0.021
	2 weeks	4	23.71	23.42	24.08	
	4 weeks	5	24.23	23.91	24.27	
	12 weeks	6	23.46	23.10	23.84	
mmu-miR-744-4395435	2 days	4	28.40	26.71	28.74	0.010
	2 weeks	4	31.48	30.73	32.03	
	4 weeks	5	31.66	31.12	32.46	
	12 weeks	6	30.84	30.66	31.28	
mmu-miR-872#-002542	2 days	4	27.01	26.89	27.42	0.004
	2 weeks	4	28.98	28.80	29.33	
	4 weeks	5	29.41	28.97	29.41	
	12 weeks	6	28.57	28.51	28.68	
mmu-miR-872-4395375	2 days	4	29.19	27.05	29.38	0.016
	2 weeks	4	30.34	29.74	30.60	
	4 weeks	5	30.80	30.29	30.84	
	12 weeks	6	30.04	29.75	30.50	
mmu-miR-877#-002548	2 days	4	28.52	28.33	28.58	0.004
	2 weeks	4	29.89	29.39	30.89	
	4 weeks	5	31.16	31.14	31.33	
	12 weeks	6	30.19	29.95	30.48	
mmu-miR-92a-4373013	2 days	4	26.85	26.83	26.96	0.009
	2 weeks	4	28.16	28.02	28.30	
	4 weeks	5	28.48	28.33	28.55	
	12 weeks	6	28.13	27.95	28.17	
mmu-miR-93-4373302	2 days	4	28.09	27.65	28.45	0.022
	2 weeks	4	28.64	28.50	28.94	
	4 weeks	5	28.72	28.57	28.94	
	12 weeks	6	28.31	28.19	28.33	
rno-miR-196c-4395750	2 days	4	30.00	29.96	30.40	0.010
	2 weeks	4	31.18	30.73	31.53	
	4 weeks	5	31.36	31.02	31.46	
	12 weeks	6	30.76	30.49	30.86	
	2 days	4	30.56	29.11	30.72	

rno-miR-345-3p-4395762	2 weeks	4	31.99	31.95	32.03	0.011
	4 weeks	5	32.04	31.91	32.28	
	12 weeks	6	31.32	30.89	31.91	
