# Supplementary material for: MicroRNA expression patterns in post-natal mouse skeletal muscle development
Source: BMC Genomics. 2017 Jan 7;18:52. doi: 10.1186/s12864-016-3399-2 (PMC5219731; doi:10.1186/s12864-016-3399-2)
Supplement: Additional file 4: — MiRNAs expression profiles were classified in 10 clusters using the predicted Ct values from a robust quadratic model. (PDF 112 kb) [file 12864_2016_3399_MOESM4_ESM.pdf]

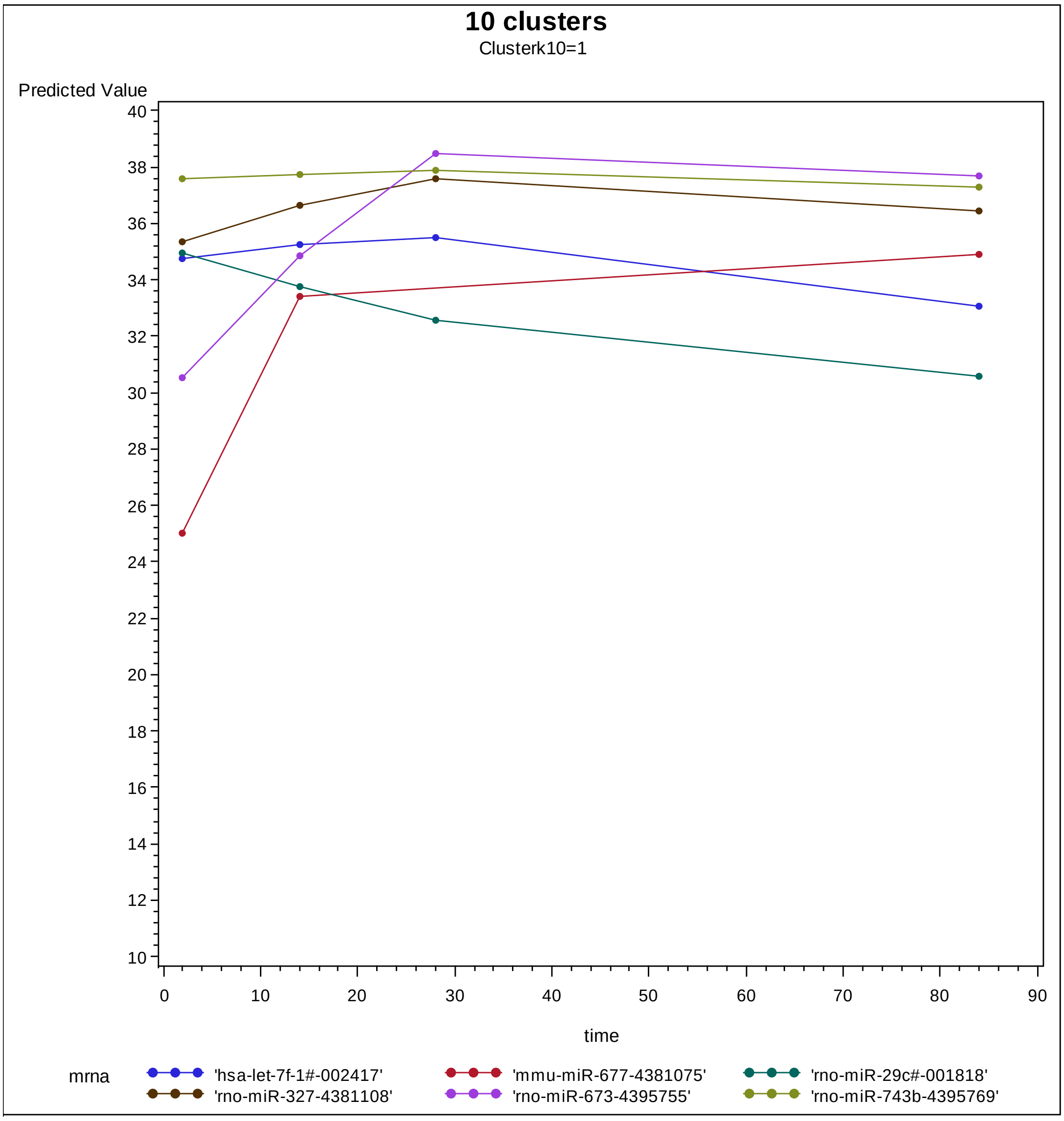

10 clusters
Clusterk10=1
Predicted Value
40
38
36
34
32
30
28
26
24
22
20
18
16
14
12
10
0
10
20
30
40
50
60
70
80
90
time
mrna
'hsa-let-7f-1#-002417'
'mmu-miR-677-4381075'
'rno-miR-29c#-001818'
'rno-miR-327-4381108'
'rno-miR-673-4395755'
'rno-miR-743b-4395769'

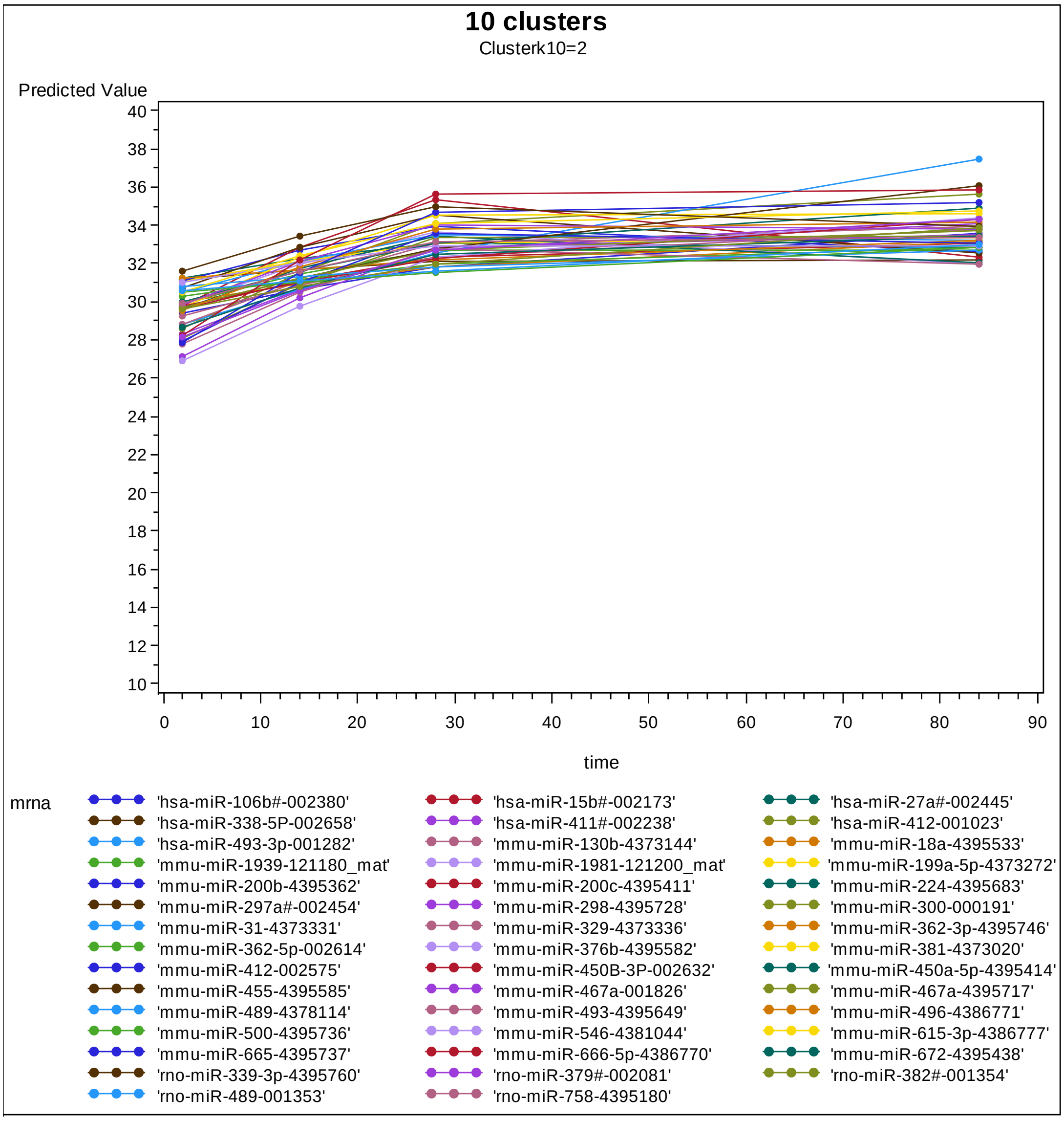
10 clusters
Clusterk10=2
Predicted Value
40
38
36
34
32
30
28
26
24
22
20
18
16
14
12
10
0
10
20
30
40
50
60
70
80
90
time
mrna
'hsa-miR-106b#-002380'
'hsa-miR-338-5P-002658'
'hsa-miR-493-3p-001282'
'mmu-miR-1939-121180_mat'
'mmu-miR-200b-4395362'
'mmu-miR-297a#-002454'
'mmu-miR-31-4373331'
'mmu-miR-362-5p-002614'
'mmu-miR-412-002575'
'mmu-miR-455-4395585'
'mmu-miR-489-4378114'
'mmu-miR-500-4395736'
'mmu-miR-665-4395737'
'rno-miR-339-3p-4395760'
'rno-miR-489-001353'
'hsa-miR-15b#-002173'
'hsa-miR-411#-002238'
'mmu-miR-130b-4373144'
'mmu-miR-1981-121200_mat'
'mmu-miR-200c-4395411'
'mmu-miR-298-4395728'
'mmu-miR-329-4373336'
'mmu-miR-376b-4395582'
'mmu-miR-450B-3P-002632'
'mmu-miR-467a-001826'
'mmu-miR-493-4395649'
'mmu-miR-546-4381044'
'mmu-miR-666-5p-4386770'
'rno-miR-379#-002081'
'rno-miR-758-4395180'
'hsa-miR-27a#-002445'
'hsa-miR-412-001023'
'mmu-miR-18a-4395533'
'mmu-miR-199a-5p-4373272'
'mmu-miR-224-4395683'
'mmu-miR-300-000191'
'mmu-miR-362-3p-4395746'
'mmu-miR-381-4373020'
'mmu-miR-450a-5p-4395414'
'mmu-miR-467a-4395717'
'mmu-miR-496-4386771'
'mmu-miR-615-3p-4386777'
'mmu-miR-672-4395438'
'rno-miR-382#-001354'

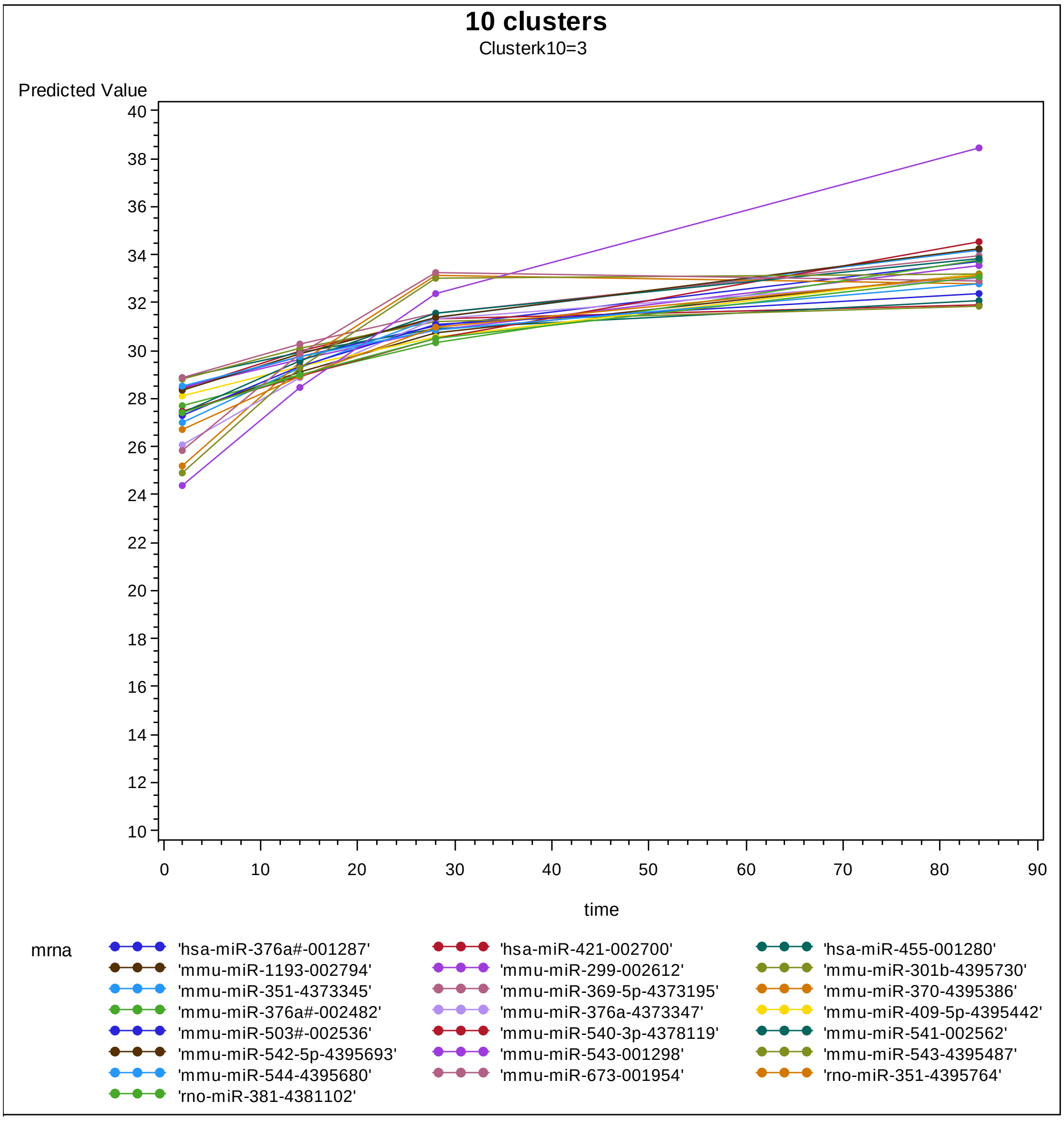
10 clusters
Clusterk10=3
Predicted Value
40
38
36
34
32
30
28
26
24
22
20
18
16
14
12
10
0
10
20
30
40
50
60
70
80
90
time
mrna
'hsa-miR-376a#-001287'
'mmu-miR-1193-002794'
'mmu-miR-351-4373345'
'mmu-miR-376a#-002482'
'mmu-miR-503#-002536'
'mmu-miR-542-5p-4395693'
'mmu-miR-544-4395680'
'rno-miR-381-4381102'
'hsa-miR-421-002700'
'mmu-miR-299-002612'
'mmu-miR-369-5p-4373195'
'mmu-miR-376a-4373347'
'mmu-miR-540-3p-4378119'
'mmu-miR-543-001298'
'mmu-miR-673-001954'
'hsa-miR-455-001280'
'mmu-miR-301b-4395730'
'mmu-miR-370-4395386'
'mmu-miR-409-5p-4395442'
'mmu-miR-541-002562'
'mmu-miR-543-4395487'
'rno-miR-351-4395764'

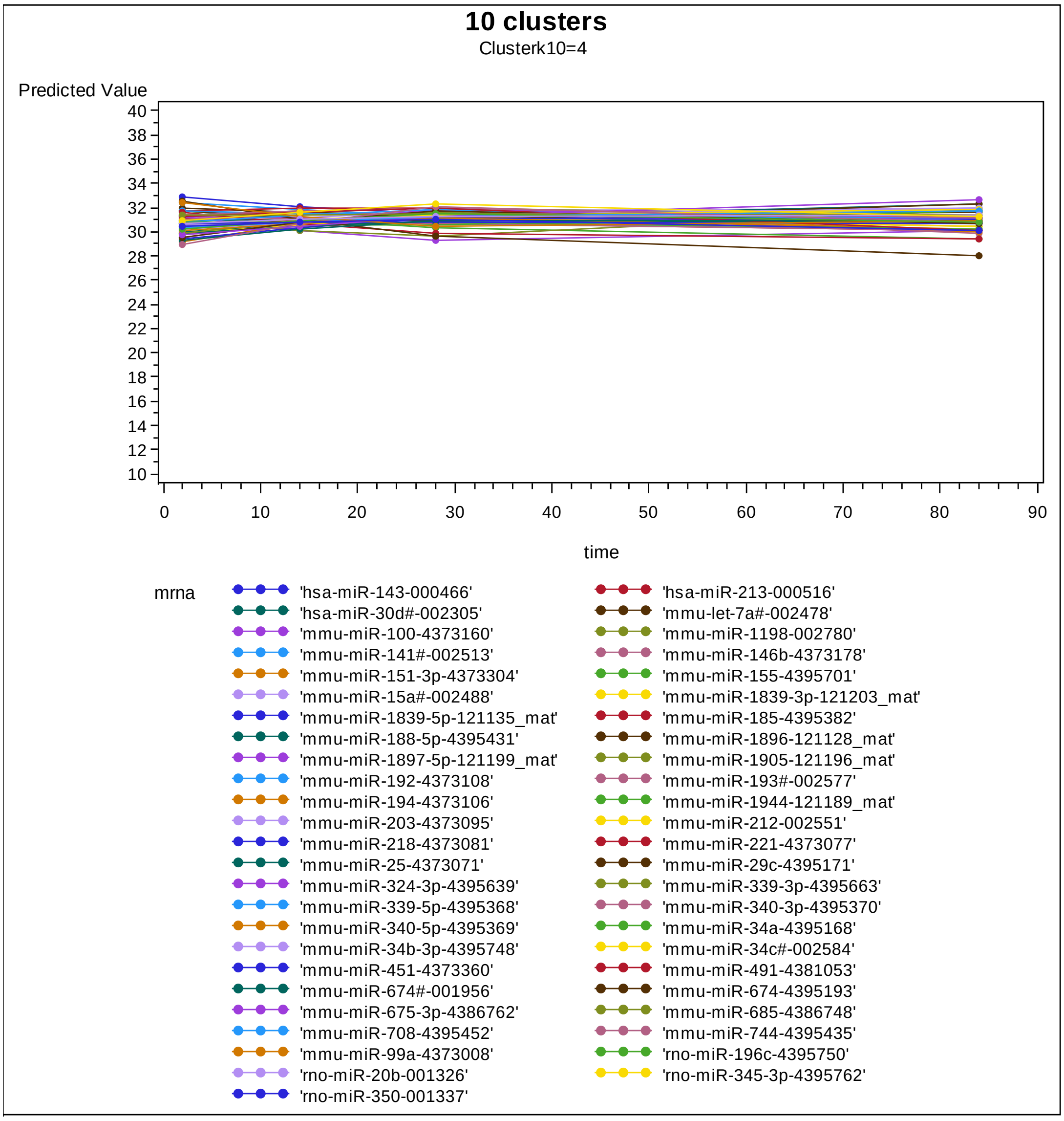
10 clusters
Clusterk10=4
Predicted Value
40
38
36
34
32
30
28
26
24
22
20
18
16
14
12
10
0
10
20
30
40
50
60
70
80
90
time
mrna
'hsa-miR-143-000466'
'hsa-miR-30d#-002305'
'mmu-miR-100-4373160'
'mmu-miR-141#-002513'
'mmu-miR-151-3p-4373304'
'mmu-miR-15a#-002488'
'mmu-miR-1839-5p-121135_mat'
'mmu-miR-188-5p-4395431'
'mmu-miR-1897-5p-121199_mat'
'mmu-miR-192-4373108'
'mmu-miR-194-4373106'
'mmu-miR-203-4373095'
'mmu-miR-218-4373081'
'mmu-miR-25-4373071'
'mmu-miR-324-3p-4395639'
'mmu-miR-339-5p-4395368'
'mmu-miR-340-5p-4395369'
'mmu-miR-34b-3p-4395748'
'mmu-miR-451-4373360'
'mmu-miR-674#-001956'
'mmu-miR-675-3p-4386762'
'mmu-miR-708-4395452'
'mmu-miR-99a-4373008'
'rno-miR-20b-001326'
'rno-miR-350-001337'
'hsa-miR-213-000516'
'mmu-let-7a#-002478'
'mmu-miR-1198-002780'
'mmu-miR-146b-4373178'
'mmu-miR-155-4395701'
'mmu-miR-1839-3p-121203_mat'
'mmu-miR-185-4395382'
'mmu-miR-1896-121128_mat'
'mmu-miR-1905-121196_mat'
'mmu-miR-193#-002577'
'mmu-miR-1944-121189_mat'
'mmu-miR-212-002551'
'mmu-miR-221-4373077'
'mmu-miR-29c-4395171'
'mmu-miR-339-3p-4395663'
'mmu-miR-340-3p-4395370'
'mmu-miR-34a-4395168'
'mmu-miR-34c#-002584'
'mmu-miR-491-4381053'
'mmu-miR-674-4395193'
'mmu-miR-685-4386748'
'mmu-miR-744-4395435'
'rno-miR-196c-4395750'
'rno-miR-345-3p-4395762'

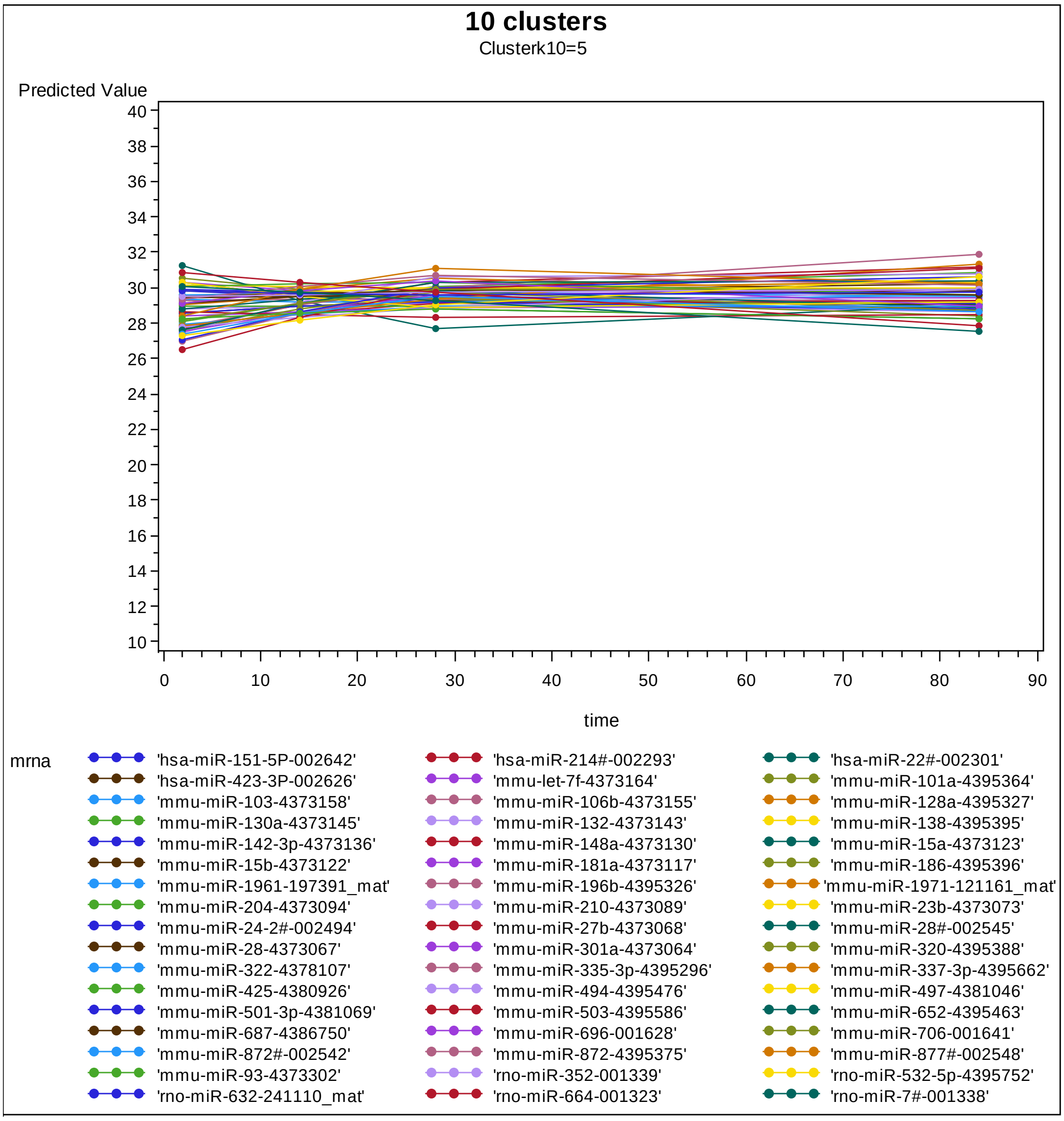
10 clusters
Clusterk10=5
Predicted Value
40
38
36
34
32
30
28
26
24
22
20
18
16
14
12
10
0
10
20
30
40
50
60
70
80
90
time
mrna
'hsa-miR-151-5P-002642'
'hsa-miR-214#-002293'
'hsa-miR-22#-002301'
'hsa-miR-423-3P-002626'
'mmu-let-7f-4373164'
'mmu-miR-101a-4395364'
'mmu-miR-103-4373158'
'mmu-miR-106b-4373155'
'mmu-miR-128a-4395327'
'mmu-miR-130a-4373145'
'mmu-miR-132-4373143'
'mmu-miR-138-4395395'
'mmu-miR-142-3p-4373136'
'mmu-miR-148a-4373130'
'mmu-miR-15a-4373123'
'mmu-miR-15b-4373122'
'mmu-miR-181a-4373117'
'mmu-miR-186-4395396'
'mmu-miR-1961-197391_mat'
'mmu-miR-196b-4395326'
'mmu-miR-1971-121161_mat'
'mmu-miR-204-4373094'
'mmu-miR-210-4373089'
'mmu-miR-23b-4373073'
'mmu-miR-24-2#-002494'
'mmu-miR-27b-4373068'
'mmu-miR-28#-002545'
'mmu-miR-28-4373067'
'mmu-miR-301a-4373064'
'mmu-miR-320-4395388'
'mmu-miR-322-4378107'
'mmu-miR-335-3p-4395296'
'mmu-miR-337-3p-4395662'
'mmu-miR-425-4380926'
'mmu-miR-494-4395476'
'mmu-miR-497-4381046'
'mmu-miR-501-3p-4381069'
'mmu-miR-503-4395586'
'mmu-miR-652-4395463'
'mmu-miR-687-4386750'
'mmu-miR-696-001628'
'mmu-miR-706-001641'
'mmu-miR-872#-002542'
'mmu-miR-872-4395375'
'mmu-miR-877#-002548'
'mmu-miR-93-4373302'
'rno-miR-352-001339'
'rno-miR-532-5p-4395752'
'rno-miR-632-241110_mat'
'rno-miR-664-001323'
'rno-miR-7#-001338'

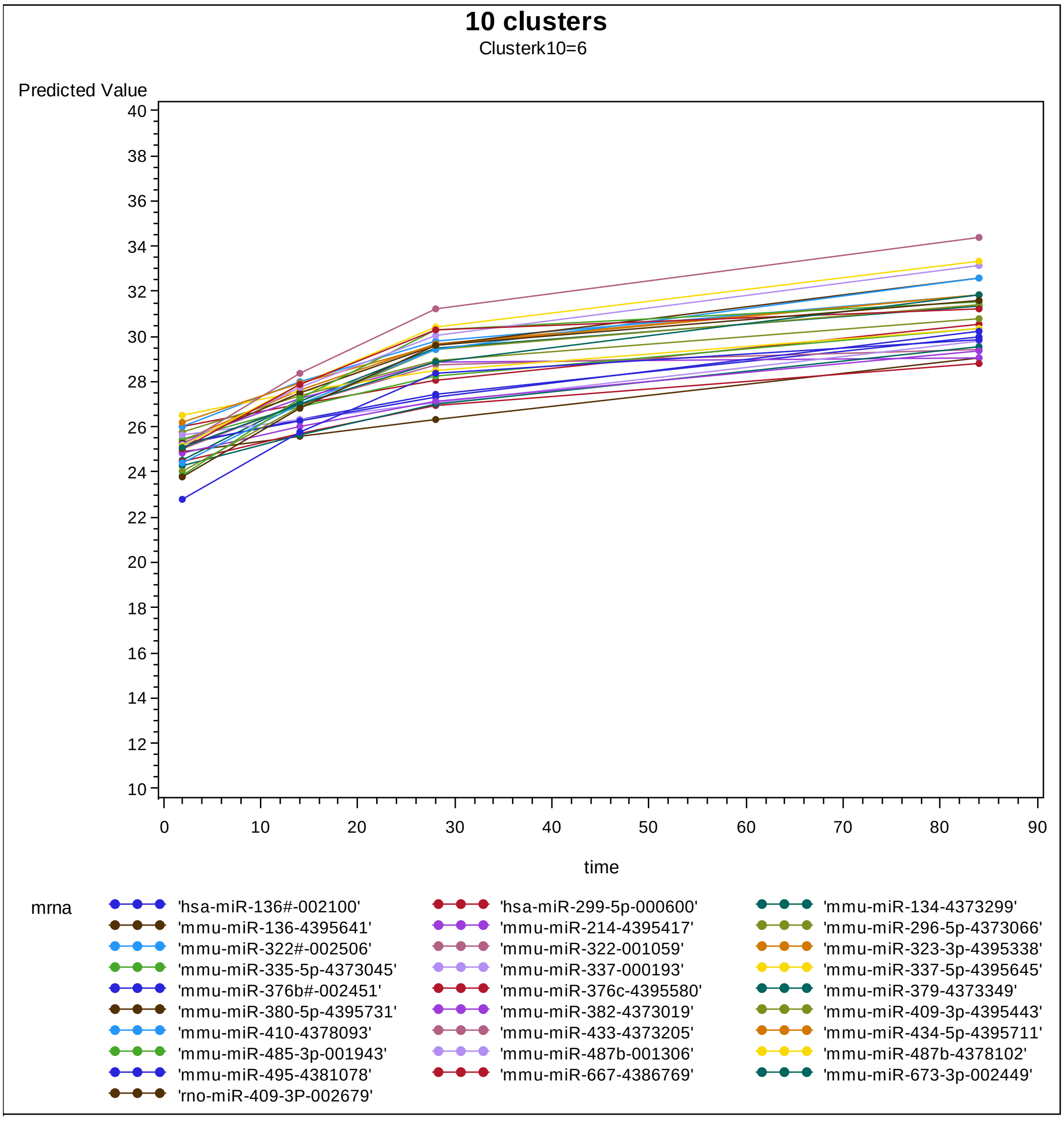

10 clusters
Clusterk10=6
Predicted Value
40
38
36
34
32
30
28
26
24
22
20
18
16
14
12
10
0
10
20
30
40
50
60
70
80
90
time
mrna
'hsa-miR-136#-002100'
'mmu-miR-136-4395641'
'mmu-miR-322#-002506'
'mmu-miR-335-5p-4373045'
'mmu-miR-376b#-002451'
'mmu-miR-380-5p-4395731'
'mmu-miR-410-4378093'
'mmu-miR-485-3p-001943'
'mmu-miR-495-4381078'
'rno-miR-409-3P-002679'
'hsa-miR-299-5p-000600'
'mmu-miR-214-4395417'
'mmu-miR-322-001059'
'mmu-miR-337-000193'
'mmu-miR-376c-4395580'
'mmu-miR-382-4373019'
'mmu-miR-433-4373205'
'mmu-miR-487b-001306'
'mmu-miR-667-4386769'
'mmu-miR-134-4373299'
'mmu-miR-296-5p-4373066'
'mmu-miR-323-3p-4395338'
'mmu-miR-337-5p-4395645'
'mmu-miR-379-4373349'
'mmu-miR-409-3p-4395443'
'mmu-miR-434-5p-4395711'
'mmu-miR-487b-4378102'
'mmu-miR-673-3p-002449'

# 10 clusters

Clusterk10=7

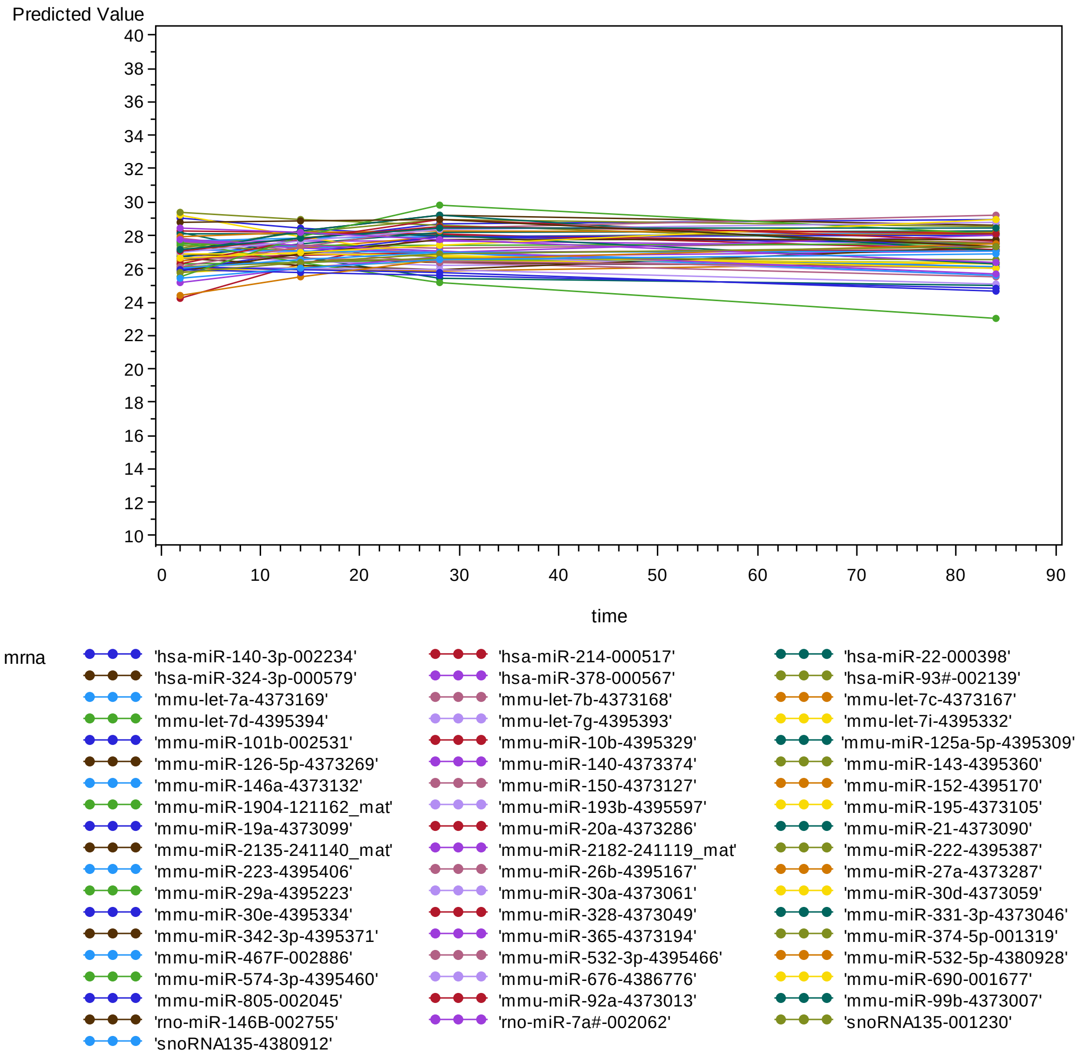

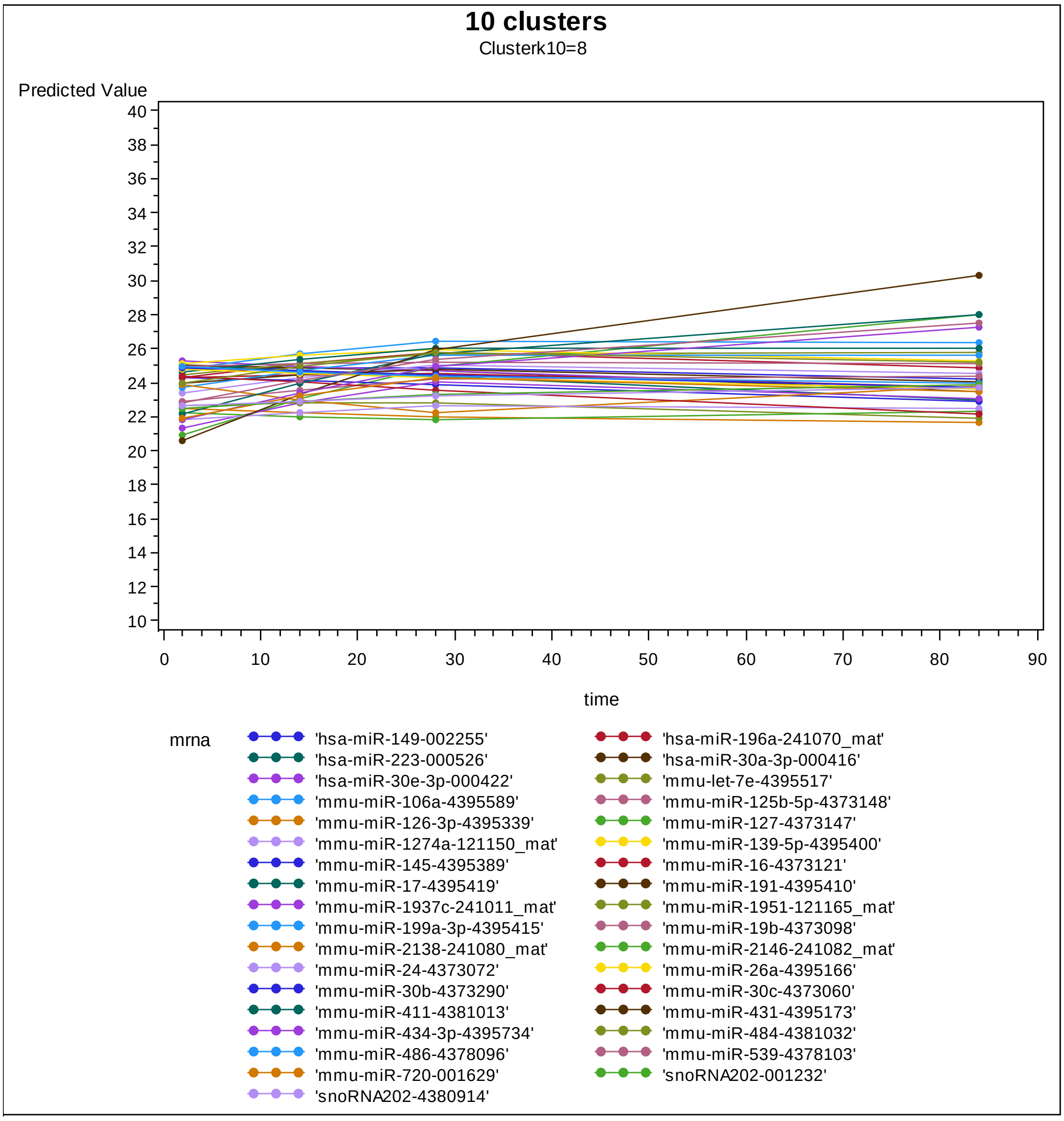
10 clusters
Clusterk10=8
Predicted Value
40
38
36
34
32
30
28
26
24
22
20
18
16
14
12
10
0
10
20
30
40
50
60
70
80
90
time
mrna
'hsa-miR-149-002255'
'hsa-miR-223-000526'
'hsa-miR-30e-3p-000422'
'mmu-miR-106a-4395589'
'mmu-miR-126-3p-4395339'
'mmu-miR-1274a-121150_mat'
'mmu-miR-145-4395389'
'mmu-miR-17-4395419'
'mmu-miR-1937c-241011_mat'
'mmu-miR-199a-3p-4395415'
'mmu-miR-2138-241080_mat'
'mmu-miR-24-4373072'
'mmu-miR-30b-4373290'
'mmu-miR-411-4381013'
'mmu-miR-434-3p-4395734'
'mmu-miR-486-4378096'
'mmu-miR-720-001629'
'snoRNA202-4380914'
'hsa-miR-196a-241070_mat'
'hsa-miR-30a-3p-000416'
'mmu-let-7e-4395517'
'mmu-miR-125b-5p-4373148'
'mmu-miR-127-4373147'
'mmu-miR-139-5p-4395400'
'mmu-miR-16-4373121'
'mmu-miR-191-4395410'
'mmu-miR-1951-121165_mat'
'mmu-miR-19b-4373098'
'mmu-miR-2146-241082_mat'
'mmu-miR-26a-4395166'
'mmu-miR-30c-4373060'
'mmu-miR-431-4395173'
'mmu-miR-484-4381032'
'mmu-miR-539-4378103'
'snoRNA202-001232'

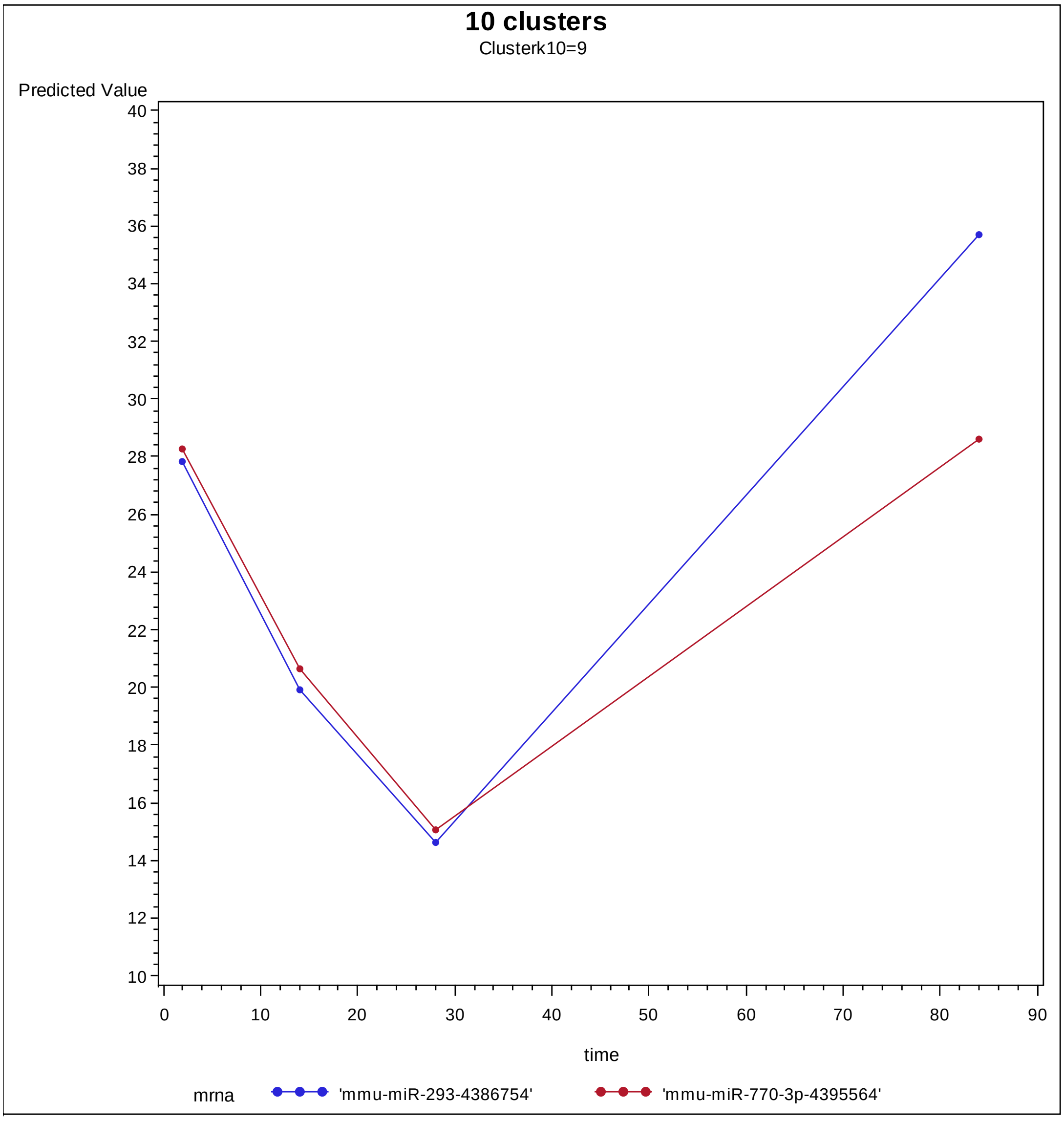
10 clusters
Clusterk10=9
Predicted Value
40
38
36
34
32
30
28
26
24
22
20
18
16
14
12
10
0
10
20
30
40
50
60
70
80
90
time
mrna
'mmu-miR-293-4386754'
'mmu-miR-770-3p-4395564'

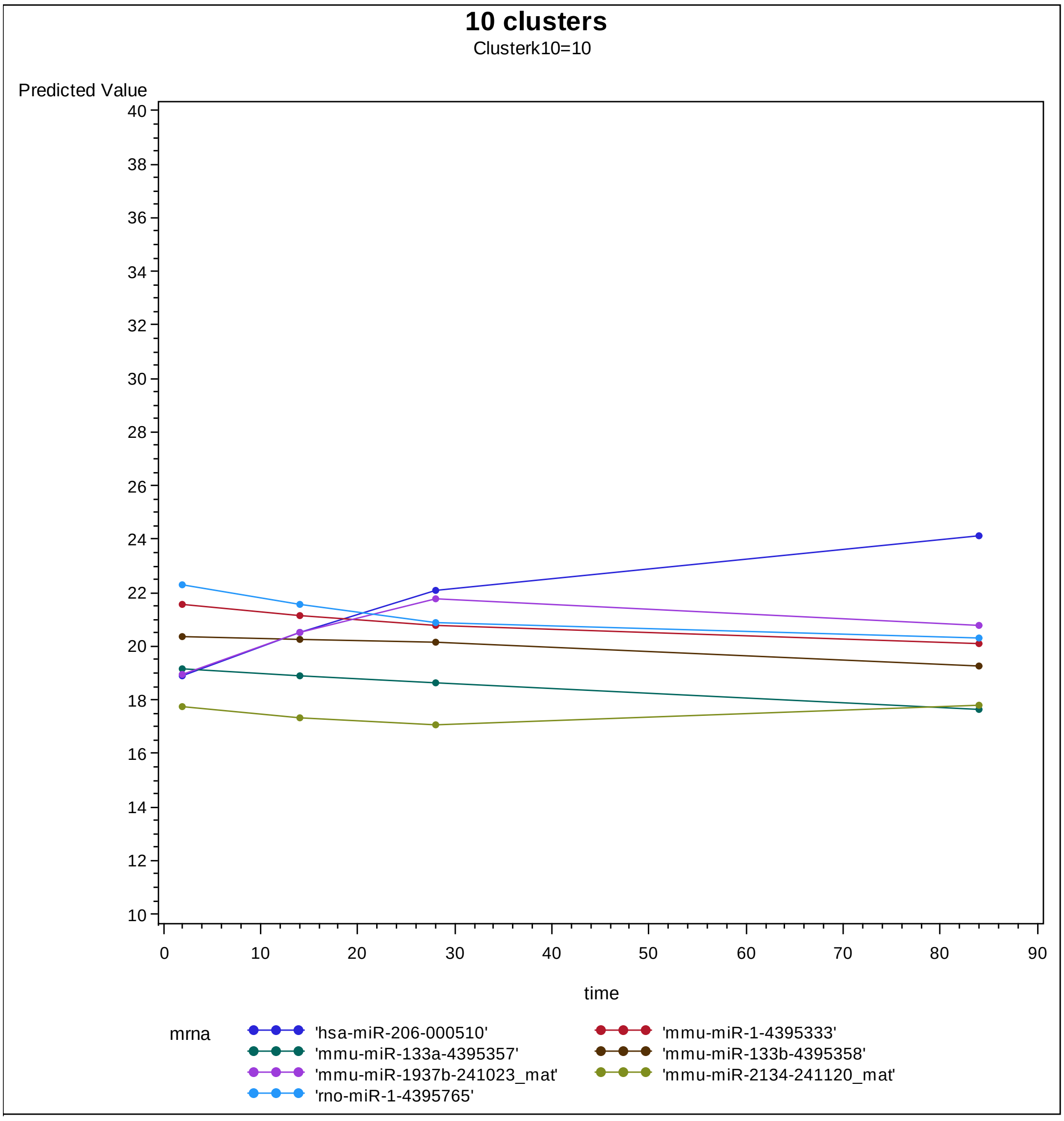

10 clusters
Clusterk10=10
Predicted Value
40
38
36
34
32
30
28
26
24
22
20
18
16
14
12
10
0
10
20
30
40
50
60
70
80
90
time
mrna
'hsa-miR-206-000510'
'mmu-miR-1-4395333'
'mmu-miR-133a-4395357'
'mmu-miR-133b-4395358'
'mmu-miR-1937b-241023_mat'
'mmu-miR-2134-241120_mat'
'rno-miR-1-4395765'
