# Supplementary material for: MicroRNA expression patterns in post-natal mouse skeletal muscle development
Source: BMC Genomics. 2017 Jan 7;18:52. doi: 10.1186/s12864-016-3399-2 (PMC5219731; doi:10.1186/s12864-016-3399-2)
Supplement: Additional file 5: — Predicted Ct values for all miRNAs from cluster A and B. (PDF 103 kb) [file 12864_2016_3399_MOESM5_ESM.pdf]

mrna	time	Clusterk10	pred_ct
'hsa-miR-106b#-002380'	2	A	29.3625
'hsa-miR-106b#-002380'	14	A	30.6202
'hsa-miR-106b#-002380'	28	A	31.809
'hsa-miR-106b#-002380'	84	A	33.5642
'hsa-miR-136#-002100'	2	B	25.2129
'hsa-miR-136#-002100'	14	B	26.3313
'hsa-miR-136#-002100'	28	B	27.4557
'hsa-miR-136#-002100'	84	B	30.0112
'hsa-miR-15b#-002173'	2	A	29.6066
'hsa-miR-15b#-002173'	14	A	31.057
'hsa-miR-15b#-002173'	28	A	32.3383
'hsa-miR-15b#-002173'	84	A	33.0383
'hsa-miR-27a#-002445'	2	A	31.2467
'hsa-miR-27a#-002445'	14	A	32.2535
'hsa-miR-27a#-002445'	28	A	33.0947
'hsa-miR-27a#-002445'	84	A	32.8688
'hsa-miR-299-5p-000600'	2	B	25.9967
'hsa-miR-299-5p-000600'	14	B	27.0031
'hsa-miR-299-5p-000600'	28	B	28.0293
'hsa-miR-299-5p-000600'	84	B	30.5398
'hsa-miR-338-5P-002658'	2	A	31.188
'hsa-miR-338-5P-002658'	14	A	31.6679
'hsa-miR-338-5P-002658'	28	A	32.0815
'hsa-miR-338-5P-002658'	84	A	32.1607
'hsa-miR-411#-002238'	2	A	28.3117
'hsa-miR-411#-002238'	14	A	30.6464
'hsa-miR-411#-002238'	28	A	32.7244
'hsa-miR-411#-002238'	84	A	34.0801
'hsa-miR-412-001023'	2	A	28.6048
'hsa-miR-412-001023'	14	A	31.5199
'hsa-miR-412-001023'	28	A	34.1013
'hsa-miR-412-001023'	84	A	35.5988
'hsa-miR-493-3p-001282'	2	A	28.8017
'hsa-miR-493-3p-001282'	14	A	30.6408
'hsa-miR-493-3p-001282'	28	A	32.5377
'hsa-miR-493-3p-001282'	84	A	37.446
'mmu-miR-130b-4373144'	2	A	28.7895
'mmu-miR-130b-4373144'	14	A	31.0694
'mmu-miR-130b-4373144'	28	A	33.0675
'mmu-miR-130b-4373144'	84	A	33.9334
'mmu-miR-134-4373299'	2	B	24.5081
'mmu-miR-134-4373299'	14	B	27.1319
'mmu-miR-134-4373299'	28	B	29.4887
'mmu-miR-134-4373299'	84	B	31.3308

'mmu-miR-136-4395641'	2	B	24.8941
'mmu-miR-136-4395641'	14	B	25.5804
'mmu-miR-136-4395641'	28	B	26.3458
'mmu-miR-136-4395641'	84	B	29.0265
'mmu-miR-18a-4395533'	2	A	29.973
'mmu-miR-18a-4395533'	14	A	30.9114
'mmu-miR-18a-4395533'	28	A	31.8001
'mmu-miR-18a-4395533'	84	A	33.1346
'mmu-miR-1939-121180_mat'	2	A	29.9126
'mmu-miR-1939-121180_mat'	14	A	31.4108
'mmu-miR-1939-121180_mat'	28	A	32.6868
'mmu-miR-1939-121180_mat'	84	A	32.7097
'mmu-miR-1981-121200_mat'	2	A	29.809
'mmu-miR-1981-121200_mat'	14	A	31.9318
'mmu-miR-1981-121200_mat'	28	A	33.6266
'mmu-miR-1981-121200_mat'	84	A	31.9879
'mmu-miR-199a-5p-4373272'	2	A	31.083
'mmu-miR-199a-5p-4373272'	14	A	32.1334
'mmu-miR-199a-5p-4373272'	28	A	33.0492
'mmu-miR-199a-5p-4373272'	84	A	33.3784
'mmu-miR-200b-4395362'	2	A	31.1011
'mmu-miR-200b-4395362'	14	A	32.6631
'mmu-miR-200b-4395362'	28	A	33.9068
'mmu-miR-200b-4395362'	84	A	32.6501
'mmu-miR-200c-4395411'	2	A	29.7455
'mmu-miR-200c-4395411'	14	A	32.8731
'mmu-miR-200c-4395411'	28	A	35.3295
'mmu-miR-200c-4395411'	84	A	32.3139
'mmu-miR-214-4395417'	2	B	25.3767
'mmu-miR-214-4395417'	14	B	27.2523
'mmu-miR-214-4395417'	28	B	28.8595
'mmu-miR-214-4395417'	84	B	29.0305
'mmu-miR-224-4395683'	2	A	29.6384
'mmu-miR-224-4395683'	14	A	31.7484
'mmu-miR-224-4395683'	28	A	33.4493
'mmu-miR-224-4395683'	84	A	32.0607
'mmu-miR-296-5p-4373066'	2	B	25.7363
'mmu-miR-296-5p-4373066'	14	B	27.3966
'mmu-miR-296-5p-4373066'	28	B	28.9344
'mmu-miR-296-5p-4373066'	84	B	30.7854
'mmu-miR-297a#-002454'	2	A	30.7456
'mmu-miR-297a#-002454'	14	A	32.8455
'mmu-miR-297a#-002454'	28	A	34.4987
'mmu-miR-297a#-002454'	84	A	32.5317
'mmu-miR-298-4395728'	2	A	27.1363
'mmu-miR-298-4395728'	14	A	30.2056
'mmu-miR-298-4395728'	28	A	32.8622
'mmu-miR-298-4395728'	84	A	33.534

'mmu-miR-300-000191'	2	A	28.0742
'mmu-miR-300-000191'	14	A	30.9214
'mmu-miR-300-000191'	28	A	33.3474
'mmu-miR-300-000191'	84	A	33.4057
'mmu-miR-31-4373331'	2	A	30.4776
'mmu-miR-31-4373331'	14	A	32.123
'mmu-miR-31-4373331'	28	A	33.5015
'mmu-miR-31-4373331'	84	A	33.1891
'mmu-miR-322#-002506'	2	B	26.0013
'mmu-miR-322#-002506'	14	B	27.9807
'mmu-miR-322#-002506'	28	B	29.8045
'mmu-miR-322#-002506'	84	B	31.8716
'mmu-miR-322-001059'	2	B	25.0135
'mmu-miR-322-001059'	14	B	27.01
'mmu-miR-322-001059'	28	B	28.753
'mmu-miR-322-001059'	84	B	29.4109
'mmu-miR-323-3p-4395338'	2	B	25.3659
'mmu-miR-323-3p-4395338'	14	B	27.6149
'mmu-miR-323-3p-4395338'	28	B	29.6737
'mmu-miR-323-3p-4395338'	84	B	31.8249
'mmu-miR-329-4373336'	2	A	29.2156
'mmu-miR-329-4373336'	14	A	30.8309
'mmu-miR-329-4373336'	28	A	32.3079
'mmu-miR-329-4373336'	84	A	33.8276
'mmu-miR-335-5p-4373045'	2	B	25.4545
'mmu-miR-335-5p-4373045'	14	B	26.8816
'mmu-miR-335-5p-4373045'	28	B	28.2401
'mmu-miR-335-5p-4373045'	84	B	30.3744
'mmu-miR-337-000193'	2	B	25.6202
'mmu-miR-337-000193'	14	B	26.2997
'mmu-miR-337-000193'	28	B	27.0613
'mmu-miR-337-000193'	84	B	29.7708
'mmu-miR-337-5p-4395645'	2	B	26.5219
'mmu-miR-337-5p-4395645'	14	B	27.5362
'mmu-miR-337-5p-4395645'	28	B	28.5238
'mmu-miR-337-5p-4395645'	84	B	30.3668
'mmu-miR-362-3p-4395746'	2	A	31.2146
'mmu-miR-362-3p-4395746'	14	A	31.6875
'mmu-miR-362-3p-4395746'	28	A	32.1625
'mmu-miR-362-3p-4395746'	84	A	33.2373
'mmu-miR-362-5p-002614'	2	A	30.2584
'mmu-miR-362-5p-002614'	14	A	31.1319
'mmu-miR-362-5p-002614'	28	A	31.9358
'mmu-miR-362-5p-002614'	84	A	32.8361
'mmu-miR-376b#-002451'	2	B	25.2473
'mmu-miR-376b#-002451'	14	B	26.242
'mmu-miR-376b#-002451'	28	B	27.2857
'mmu-miR-376b#-002451'	84	B	30.2026

'mmu-miR-376b-4395582'	2	A	26.8995
'mmu-miR-376b-4395582'	14	A	29.7569
'mmu-miR-376b-4395582'	28	A	32.3263
'mmu-miR-376b-4395582'	84	A	34.3726
'mmu-miR-376c-4395580'	2	B	24.4384
'mmu-miR-376c-4395580'	14	B	25.7202
'mmu-miR-376c-4395580'	28	B	26.9378
'mmu-miR-376c-4395580'	84	B	28.8171
'mmu-miR-379-4373349'	2	B	24.2481
'mmu-miR-379-4373349'	14	B	25.633
'mmu-miR-379-4373349'	28	B	26.9858
'mmu-miR-379-4373349'	84	B	29.5663
'mmu-miR-380-5p-4395731'	2	B	25.2486
'mmu-miR-380-5p-4395731'	14	B	27.504
'mmu-miR-380-5p-4395731'	28	B	29.6244
'mmu-miR-380-5p-4395731'	84	B	32.6044
'mmu-miR-381-4373020'	2	A	29.7112
'mmu-miR-381-4373020'	14	A	32.3057
'mmu-miR-381-4373020'	28	A	34.5183
'mmu-miR-381-4373020'	84	A	34.6009
'mmu-miR-382-4373019'	2	B	24.8067
'mmu-miR-382-4373019'	14	B	25.9939
'mmu-miR-382-4373019'	28	B	27.1525
'mmu-miR-382-4373019'	84	B	29.3485
'mmu-miR-409-3p-4395443'	2	B	24.0214
'mmu-miR-409-3p-4395443'	14	B	26.8815
'mmu-miR-409-3p-4395443'	28	B	29.4477
'mmu-miR-409-3p-4395443'	84	B	31.4146
'mmu-miR-410-4378093'	2	B	24.4265
'mmu-miR-410-4378093'	14	B	26.9961
'mmu-miR-410-4378093'	28	B	29.3998
'mmu-miR-410-4378093'	84	B	32.6157
'mmu-miR-412-002575'	2	A	27.9405
'mmu-miR-412-002575'	14	A	31.0022
'mmu-miR-412-002575'	28	A	33.572
'mmu-miR-412-002575'	84	A	33.059
'mmu-miR-433-4373205'	2	B	25.2024
'mmu-miR-433-4373205'	14	B	28.3475
'mmu-miR-433-4373205'	28	B	31.237
'mmu-miR-433-4373205'	84	B	34.3972
'mmu-miR-434-5p-4395711'	2	B	26.2049
'mmu-miR-434-5p-4395711'	14	B	27.9595
'mmu-miR-434-5p-4395711'	28	B	29.6019
'mmu-miR-434-5p-4395711'	84	B	31.8139
'mmu-miR-450B-3P-002632'	2	A	29.7367
'mmu-miR-450B-3P-002632'	14	A	31.03
'mmu-miR-450B-3P-002632'	28	A	32.2686
'mmu-miR-450B-3P-002632'	84	A	34.3119

'mmu-miR-450a-5p-4395414'	2	A	29.961
'mmu-miR-450a-5p-4395414'	14	A	31.564
'mmu-miR-450a-5p-4395414'	28	A	33.0511
'mmu-miR-450a-5p-4395414'	84	A	34.8728
'mmu-miR-455-4395585'	2	A	29.6953
'mmu-miR-455-4395585'	14	A	31.2508
'mmu-miR-455-4395585'	28	A	32.7965
'mmu-miR-455-4395585'	84	A	36.0822
'mmu-miR-467a-001826'	2	A	29.8434
'mmu-miR-467a-001826'	14	A	32.0951
'mmu-miR-467a-001826'	28	A	33.9987
'mmu-miR-467a-001826'	84	A	33.8242
'mmu-miR-467a-4395717'	2	A	29.6544
'mmu-miR-467a-4395717'	14	A	31.2386
'mmu-miR-467a-4395717'	28	A	32.6604
'mmu-miR-467a-4395717'	84	A	33.753
'mmu-miR-485-3p-001943'	2	B	23.8482
'mmu-miR-485-3p-001943'	14	B	27.2768
'mmu-miR-485-3p-001943'	28	B	30.2768
'mmu-miR-485-3p-001943'	84	B	31.5072
'mmu-miR-487b-001306'	2	B	25.2137
'mmu-miR-487b-001306'	14	B	27.7257
'mmu-miR-487b-001306'	28	B	30.0703
'mmu-miR-487b-001306'	84	B	33.1364
'mmu-miR-487b-4378102'	2	B	25.1609
'mmu-miR-487b-4378102'	14	B	27.8902
'mmu-miR-487b-4378102'	28	B	30.4107
'mmu-miR-487b-4378102'	84	B	33.3464
'mmu-miR-489-4378114'	2	A	30.7602
'mmu-miR-489-4378114'	14	A	31.2826
'mmu-miR-489-4378114'	28	A	31.788
'mmu-miR-489-4378114'	84	A	32.6881
'mmu-miR-493-4395649'	2	A	27.7721
'mmu-miR-493-4395649'	14	A	30.4918
'mmu-miR-493-4395649'	28	A	32.7518
'mmu-miR-493-4395649'	84	A	31.958
'mmu-miR-495-4381078'	2	B	22.7943
'mmu-miR-495-4381078'	14	B	25.7498
'mmu-miR-495-4381078'	28	B	28.3652
'mmu-miR-495-4381078'	84	B	29.8585
'mmu-miR-496-4386771'	2	A	29.5289
'mmu-miR-496-4386771'	14	A	31.833
'mmu-miR-496-4386771'	28	A	33.8177
'mmu-miR-496-4386771'	84	A	34.1807
'mmu-miR-500-4395736'	2	A	30.4811
'mmu-miR-500-4395736'	14	A	30.9986
'mmu-miR-500-4395736'	28	A	31.5266
'mmu-miR-500-4395736'	84	A	32.8238

'mmu-miR-546-4381044'	2	A	30.9896
'mmu-miR-546-4381044'	14	A	32.0148
'mmu-miR-546-4381044'	28	A	32.8879
'mmu-miR-546-4381044'	84	A	32.9015
'mmu-miR-615-3p-4386777'	2	A	30.4598
'mmu-miR-615-3p-4386777'	14	A	32.3879
'mmu-miR-615-3p-4386777'	28	A	34.072
'mmu-miR-615-3p-4386777'	84	A	34.7204
'mmu-miR-665-4395737'	2	A	27.8387
'mmu-miR-665-4395737'	14	A	31.509
'mmu-miR-665-4395737'	28	A	34.6661
'mmu-miR-665-4395737'	84	A	35.1789
'mmu-miR-666-5p-4386770'	2	A	28.2145
'mmu-miR-666-5p-4386770'	14	A	32.1928
'mmu-miR-666-5p-4386770'	28	A	35.5929
'mmu-miR-666-5p-4386770'	84	A	35.8278
'mmu-miR-667-4386769'	2	B	25.0352
'mmu-miR-667-4386769'	14	B	27.8518
'mmu-miR-667-4386769'	28	B	30.3092
'mmu-miR-667-4386769'	84	B	31.2149
'mmu-miR-672-4395438'	2	A	28.6821
'mmu-miR-672-4395438'	14	A	30.6885
'mmu-miR-672-4395438'	28	A	32.459
'mmu-miR-672-4395438'	84	A	33.3996
'mmu-miR-673-3p-002449'	2	B	25.102
'mmu-miR-673-3p-002449'	14	B	27.0266
'mmu-miR-673-3p-002449'	28	B	28.8649
'mmu-miR-673-3p-002449'	84	B	31.8342
'rno-miR-339-3p-4395760'	2	A	31.5599
'rno-miR-339-3p-4395760'	14	A	33.4439
'rno-miR-339-3p-4395760'	28	A	34.9751
'rno-miR-339-3p-4395760'	84	A	33.9184
'rno-miR-379#-002081'	2	A	28.1819
'rno-miR-379#-002081'	14	A	30.5634
'rno-miR-379#-002081'	28	A	32.6969
'rno-miR-379#-002081'	84	A	34.2852
'rno-miR-382#-001354'	2	A	29.6138
'rno-miR-382#-001354'	14	A	30.8194
'rno-miR-382#-001354'	28	A	31.9729
'rno-miR-382#-001354'	84	A	33.8614
'rno-miR-409-3P-002679'	2	B	23.7666
'rno-miR-409-3P-002679'	14	B	26.8467
'rno-miR-409-3P-002679'	28	B	29.5999
'rno-miR-409-3P-002679'	84	B	31.5632
'rno-miR-489-001353'	2	A	30.5477
'rno-miR-489-001353'	14	A	31.0693
'rno-miR-489-001353'	28	A	31.6067
'rno-miR-489-001353'	84	A	32.9917

'rno-miR-758-4395180'	2	A	29.8765
'rno-miR-758-4395180'	14	A	31.6164
'rno-miR-758-4395180'	28	A	33.1114
'rno-miR-758-4395180'	84	A	33.3311
